# Incidence, co-occurrence, and evolution of long-COVID features: A 6-month retrospective cohort study of 273,618 survivors of COVID-19

**DOI:** 10.1371/journal.pmed.1003773

**Published:** 2021-09-28

**Authors:** Maxime Taquet, Quentin Dercon, Sierra Luciano, John R. Geddes, Masud Husain, Paul J. Harrison

**Affiliations:** 1 Department of Psychiatry, University of Oxford, United Kingdom; 2 Oxford Health NHS Foundation Trust, Oxford, United Kingdom; 3 TriNetX Inc., Cambridge, Massachusetts, United States of America; 4 Nuffield Department of Clinical Neurosciences, University of Oxford, United Kingdom; 5 Oxford University Hospitals NHS Foundation Trust, Oxford, United Kingdom; Universitair Medisch Centrum Utrecht, NETHERLANDS

## Abstract

**Background:**

Long-COVID refers to a variety of symptoms affecting different organs reported by people following Coronavirus Disease 2019 (COVID-19) infection. To date, there have been no robust estimates of the incidence and co-occurrence of long-COVID features, their relationship to age, sex, or severity of infection, and the extent to which they are specific to COVID-19. The aim of this study is to address these issues.

**Methods and findings:**

We conducted a retrospective cohort study based on linked electronic health records (EHRs) data from 81 million patients including 273,618 COVID-19 survivors. The incidence and co-occurrence within 6 months and in the 3 to 6 months after COVID-19 diagnosis were calculated for 9 core features of long-COVID (breathing difficulties/breathlessness, fatigue/malaise, chest/throat pain, headache, abdominal symptoms, myalgia, other pain, cognitive symptoms, and anxiety/depression). Their co-occurrence network was also analyzed. Comparison with a propensity score–matched cohort of patients diagnosed with influenza during the same time period was achieved using Kaplan–Meier analysis and the Cox proportional hazard model. The incidence of atopic dermatitis was used as a negative control.

Among COVID-19 survivors (mean [SD] age: 46.3 [19.8], 55.6% female), 57.00% had one or more long-COVID feature recorded during the whole 6-month period (i.e., including the acute phase), and 36.55% between 3 and 6 months. The incidence of each feature was: abnormal breathing (18.71% in the 1- to 180-day period; 7.94% in the 90- to180-day period), fatigue/malaise (12.82%; 5.87%), chest/throat pain (12.60%; 5.71%), headache (8.67%; 4.63%), other pain (11.60%; 7.19%), abdominal symptoms (15.58%; 8.29%), myalgia (3.24%; 1.54%), cognitive symptoms (7.88%; 3.95%), and anxiety/depression (22.82%; 15.49%). All 9 features were more frequently reported after COVID-19 than after influenza (with an overall excess incidence of 16.60% and hazard ratios between 1.44 and 2.04, all *p* < 0.001), co-occurred more commonly, and formed a more interconnected network. Significant differences in incidence and co-occurrence were associated with sex, age, and illness severity. Besides the limitations inherent to EHR data, limitations of this study include that (i) the findings do not generalize to patients who have had COVID-19 but were not diagnosed, nor to patients who do not seek or receive medical attention when experiencing symptoms of long-COVID; (ii) the findings say nothing about the persistence of the clinical features; and (iii) the difference between cohorts might be affected by one cohort seeking or receiving more medical attention for their symptoms.

**Conclusions:**

Long-COVID clinical features occurred and co-occurred frequently and showed some specificity to COVID-19, though they were also observed after influenza. Different long-COVID clinical profiles were observed based on demographics and illness severity.

## Abstract 2

Maxime Taquet and colleagues investigate the incidence, co-occurrence and evolution of long-COVID features in more than a quarter of a million people.

## Author summary

**Why was this study done?:**

- Long-COVID has been described in recent studies. But we do not know the risk of developing features of this condition and how it is affected by factors such as age, sex, or severity of infection.
- We do not know if the risk of having features of long-COVID is more likely after Coronavirus Disease 2019 (COVID-19) than after influenza.
- We do not know about the extent to which different features of long-COVID co-occur.

**What did the researchers do and find?:**

- This research used data from electronic health records of 273,618 patients diagnosed with COVID-19 and estimated the risk of having long-COVID features in the 6 months after a diagnosis of COVID-19. It compared the risk of long-COVID features in different groups within the population and also compared the risk to that after influenza.
- The research found that over 1 in 3 patients had one or more features of long-COVID recorded between 3 and 6 months after a diagnosis of COVID-19. This was significantly higher than after influenza.
- For 2 in 5 of the patients who had long-COVID features in the 3- to 6-month period, they had no record of any such feature in the previous 3 months.
- The risk of long-COVID features was higher in patients who had more severe COVID-19 illness, and slightly higher among females and young adults. White and non-white patients were equally affected.

**What do these findings mean?:**

- Knowing the risk of long-COVID features helps in planning the relevant healthcare service provision.
- The fact that the risk is higher after COVID-19 than after influenza suggests that their origin might, in part, directly involve infection with SARS-CoV-2 and is not just a general consequence of viral infection. This might help in developing effective treatments against long-COVID.
- The findings in the subgroups, and the fact that the majority of patients who have features of long-COVID in the 3- to 6-month period already had symptoms in the first 3 months, may help in identifying those at greatest risk.

## Introduction

There is increasing concern, and emerging evidence, that some people who contract Coronavirus Disease 2019 (COVID-19) do not make a rapid or full recovery—so-called “long-COVID” or “long-haulers” [1–3]. The term, and the related “post-acute COVID-19 syndrome” [4] encompasses a range of features indicative of involvement of many organs affecting people weeks and months after infection. Although a long-COVID syndrome remains to be defined, prominent reported features include breathlessness, headache, chest pain, abdominal symptoms, myalgia, fatigue, cognitive difficulties, as well as anxiety and depression [5–9]. We use the term “features,” since as currently conceptualized, they are a mixture of symptoms and diagnoses.

Although several studies have addressed the issue of long-term sequelae of COVID-19, they all have some important limitations. For example, the results of a telephone survey in France (with a 57% response rate reaching 478 patients) showed that at 4 months after hospitalization for COVID-19, about half the patients had at least one feature of long-COVID [10]. In an app-based cohort study with 4,182 cases of COVID-19, 13% of respondents self-reported long-COVID features, with some evidence for higher rates in women and older people [11]. Another investigation followed 1,733 patients hospitalized for COVID-19 for 6 months and found fatigue or muscle weakness in 63%, sleep difficulties in 26%, anxiety or depression in 23%, and lower rates of myalgia and headache [12]. These studies lack a control group and have limited generalizability, focusing either on hospitalized patients or individuals who voluntarily responded to a telephone survey or used an app.

Some investigators have compared long-term sequelae of COVID-19 to those occurring after influenza. A 6-month retrospective cohort study based on electronic health records (EHRs) of 236,000 COVID-19 patients found higher rates of anxiety and mood disorders, insomnia, and dementia post-COVID-19 than after influenza [13]. Another report, based on EHRs from American veterans (88% male) has also identified increased rates of sequelae in multiple body systems after COVID-19 compared to influenza [14]. To our knowledge, though there have been no robust estimates, in a general population, of the incidence and co-occurrence of long-COVID features, their relationship to age, sex, or severity of infection, and the relative risk after COVID-19 compared to influenza. Using EHRs, we sought to address these questions.

## Methods

### Analysis plan

The study protocol (not included in the supporting information), including cohorts definition, covariates, and outcomes, was planned before it was executed and almost exactly followed the protocol used in previous studies based on the same dataset (except for the outcomes that are specific to this study) [13,15]. The differences with the protocols of the previous studies were defined before the analysis was executed and were motivated by accrued knowledge about COVID-19 and its risk factors (e.g., psychiatric illness was added as a covariate given its association with risk of COVID-19). No change to the protocol for the primary analysis and the analysis of subgroups was brought after the analysis started. The other secondary analyses were motivated by the results of the primary analysis and by interactions with the reviewers of this article.

### Data

The study used TriNetX Analytics, a federated network of linked EHRs recording anonymized data from 59 healthcare organizations (HCOs), primarily in the United States of America, totaling 81 million patients. Available data include demographics, diagnoses (using ICD-10 codes), and measurements (e.g., leukocytes count). The HCOs in the network are a mixture of hospitals, primary care, and specialist providers, and they contribute data from uninsured and insured patients. The process by which the data is deidentified is attested to through a formal determination by a qualified expert as defined in Section §164.514(b)(1) of the HIPAA Privacy Rule. This formal determination by a qualified expert, refreshed in December 2020, supersedes TriNetX’s waiver from the Western Institutional Review Board (IRB). For further details about TriNetX, its data, provenance, and functionalities, see Supporting Methods A in S1 Text.

### Cohorts

The primary cohort was defined as all patients who had a confirmed diagnosis of COVID-19 (ICD-10 code U07.1). It was compared to a matched cohort of patients diagnosed with influenza (ICD-10 codes J09-J11) who did not have a diagnosis of COVID-19 or a positive test for COVID-19. Cohorts included all patients over the age of 10 who had the index event (COVID-19 or influenza) on or after January 20, 2020 (the date of the first recorded COVID-19 case in the USA) and who were still alive at the end of follow-up (December 16, 2020). We note that patients in the influenza cohort must have presented in order to have been diagnosed, and, hence, the cohort is likely enriched for those who had a more severe form of the illness. Further details on cohorts are provided in Supporting Methods B in S1 Text.

### Covariates

A set of established and suspected risk factors for COVID-19 and for more severe COVID-19 illness was used [15–17]: age, sex, race, ethnicity, obesity, hypertension, diabetes, chronic kidney disease, asthma, chronic lower respiratory diseases, nicotine dependence, substance misuse, previous psychiatric illness, ischemic heart disease, and other forms of heart disease, socioeconomic deprivation, cancer (and hematological cancer in particular), chronic liver disease, stroke, dementia, organ transplant, rheumatoid arthritis, lupus, psoriasis, and disorders involving an immune mechanism. To capture these risk factors in patients’ health records, 58 variables were used. More details including ICD-10 codes are presented in Supporting Methods C in S1 Text. Cohorts were matched for all these variables, as described below.

### Outcomes

In the absence of consensus as to what constitutes long-COVID, we adopted a pragmatic approach that attempted to capture features common to most descriptions, and which accounted for the fact that some features are diagnoses whereas others are symptoms. The 9 clinical features were as follows:

- chest/throat pain;
- abnormal breathing;
- abdominal symptoms;
- fatigue/malaise;
- anxiety/depression;
- pain;
- headache;
- cognitive dysfunction; and
- myalgia.

We defined each clinical feature using all ICD-10 codes we considered to be encompassed by the term. For example, to capture “abdominal symptoms,” we included abdominal pain, change of bowel habit, and diarrhea. We included atopic dermatitis as a negative control [18]. See Supporting Methods D in S1 Text for ICD-10 codes used for each feature.

The main outcomes for the analysis were the 9 long-COVID features. Their incidence and co-occurrence were analyzed both over the whole period from 1 to 180 days after the index event and specifically over the “long” phase, i.e., 90 to 180 days post-diagnosis (see below). In Kaplan–Meier analysis, outcomes are only counted the first time they occur in the follow-up period. Thus, if a patient has a feature recorded once in the acute phase of the illness and again a few months later, the latter occurrence would not count toward the incidence at later stages of follow-up. This implies that, in the 1- to 180-day follow-up, ongoing incidence after 3 months represents emergence of new long-COVID features in an individual with no record of these features before. Results from the “long” phase, on the other hand, assess the overall incidence of long-COVID features in the 90 to 180 days post-diagnosis (i.e., including patients who already experienced these features in the first 3 months and are experiencing them again). Jointly analyzing the incidence in these 2 time windows allowed us to separately estimate the rate of first occurrences and the rate of recurrences of long-COVID features (where recurrences are defined as a feature, which is recorded in the 90- to 180-day window but which was also recorded in the 1- to 90-day window).

The choice to start the “long” phase at 90 days was motivated by an emerging consensus that this corresponds to the longer phase of the illness [11]. The choice to end the follow-up at 6 months was a pragmatic one, aiming to have a long-enough follow-up while making sure that enough individuals contribute data at the end of the follow-up (taking a much longer follow-up would mean that only patients diagnosed early in the pandemic would be contributing data). This is also consistent with our prior study on the long-term sequelae of COVID-19 wherein a 6-month follow-up was used [13].

### Statistical analyses

Propensity score 1:1 matching [19] (with greedy nearest neighbor matching, and a caliper distance of 0.1 pooled standard deviations of the logit of the propensity score) was used to create cohorts with matched baseline characteristics and carried out within the TriNetX network. Characteristics with a standardized mean difference (SMD) between cohorts ≤0.1 was considered well matched [20]. Because we used EHR with coded health events, if an event was not present, it was considered absent. Missing data for race and ethnicity were assigned their own category and that category was included in the propensity score matching, so that the 2 matched cohorts had approximately equal numbers of patients with unknown race/ethnicity.

We first investigated the incidence and co-occurrence of long-COVID features over the 180 days following a COVID-19 diagnosis and specifically over the 90- to 180-day period using Kaplan–Meier analysis. Co-occurrence consists in the occurrence in the same patient of a pair of features. We also separately calculated the incidence of each feature occurring in the 1- to 90-day period only, in the 90- to 180-day period only, and the rate of recurrences (i.e., the incidence of features, which occurred in the 90- to 180-day period and which had already occurred in the 1- to 90-day period). This was achieved using simple algebraic relations between these and the recorded incidences over the whole 1 to 180 days and the “long” 90- to 180-day period (see Supporting Methods E in S1 Text for details).

Incidences and co-occurrences were compared with those occurring in the matched control cohort using the Cox proportional hazard model. The proportional hazard assumption was tested using the generalized Schoenfeld approach. When violated, time-varying hazard ratios (HRs) were assessed using natural cubic splines fitted to the log-cumulative hazard [21]. The absolute risk increase was also calculated between the 2 matched cohorts for each clinical feature by subtracting the cumulative incidence in the influenza cohort from that in the COVID-19 cohort at the end of the time windows (for both the 1- to 180-day and the 90- to 180-day windows).

To assess whether long-COVID features co-occur more often after COVID-19 than after influenza, above and beyond what their individual incidences would predict, we calculated Dice’s coefficient (*D*), which is the probability of co-occurrence divided by the mean of individual incidences [22]. It varies between 0 and 1. For a pair of features, *D* = 1 means that one occurs if and only if the other one occurs, and *D* = 0 means that the two never co-occur.

Clinical features can be represented as the nodes of a network whose connections are weighted by Dice’s coefficient for the pair of features they connect. A strongly interconnected network indicates that when a clinical feature occurs, it tends to co-occur with other features, whereas a weakly interconnected network indicates that the features tend to occur in isolation. The average degree of a network (the sum of the connections of each node with other nodes, averaged over all nodes) is a standard measure of how interconnected the network is [23]. We calculated the average degree of the clinical feature network for both the COVID-19 and influenza cohorts, calculated the effect size of the difference, and tested whether there was a significant difference between the two using permutation tests with 1,000 permutations.

All confidence intervals (CIs) were calculated using nonparametric bootstrap with 1,000 replicates. Statistical analyses were conducted in R version 3.6.3. Statistical significance was set at two-sided *p*-values < 0.05. Details of the statistical analyses can be found in Supporting Methods E in S1 Text.

### Secondary analyses

We assessed whether the incidence and co-occurrence of long-COVID features vary with differences in demographics or with the severity of the COVID-19 illness by conducting 8 further cohort studies, comparing subgroups of COVID-19 patients: (1) female versus male; (2) non-white versus white; (3) age 45 and over versus age 10 to 44; (4) age 65 and over versus age 45 to 64; (5) age 22 to 44 versus age 10 to 21; (6) patients requiring versus not requiring hospitalization; (7) patients requiring versus not requiring intensive treatment unit (ITU) care; and (8) patients with versus without leukocytosis (defined as a leukocyte count over 11,000 per microliter) [24]. Subgroups (6) to (8) were measured between 4 days before and 14 days after the date of diagnosis of COVID-19 or influenza. Leukocytosis was used as leukocyte count was one of the most commonly reported inflammatory markers in the sample. Each pair of cohorts were matched for the 58 covariates and compared using the same approach as described for the primary analysis.

As an approach to assess differences in the burden of each clinical feature among patients who have them, we counted the number of occurrences of each clinical feature in the 6 months after the index event (this part of the analysis was conducted on July 18, 2021, so that all patients diagnosed on or before December 16, 2020 had over 6 months of follow-up available) and compared this between the COVID-19 and influenza cohorts. Restricting the cohorts to those with at least one record of the feature guarantees that we are not merely replicating the main analysis in which the cohorts are compared based on the presence of the feature. We view the number of occurrences as being a composite of persistence of the original diagnosis, and a true recurrence. The mean and bootstrap-derived 95% CIs for the count of occurrences are reported, and the null hypothesis that there is no difference in the number of occurrences between the 2 cohorts was tested using a Poisson regression.

Details of the secondary analyses are presented in Supporting Methods F in S1 Text. This report complies with the STROBE reporting guideline (see S1 STROBE Checklist).

## Results

A total of 273,618 patients with COVID-19 were identified, and 114,449 patients with influenza were available for matching. Table 1 (and Tables A and B in S1 Tables) summarizes the baseline characteristics, and incidence of the 9 long-COVID features, for the full COVID-19 cohort (*n =* 273,618, mean [SD] age 46.3 [19.8] years, 55.6% female), as well as for the matched COVID-19 and influenza cohorts (*n* = 106,578). Adequate propensity score matching (SMD ≤ 0.1) was achieved for all comparisons.

**Table 1:** COVID-19 cohort, and for COVID-19 and influenza cohorts after propensity score matching. Only characteristics with a prevalence higher than 5% in the unmatched COVID-19 cohort are presented here; for additional baseline characteristics and outcomes, see Tables A and B in S1 Tables.

	COVID-19 (unmatched)	COVID-19 (matched)	Influenza (matched)
**COHORT SIZE**	273,618	106,578	106,578
**DEMOGRAPHICS**			
**Age**; mean (SD); y	46.3 (19.8)	39.4 (18.4)	38.3 (19.7)
**Sex**; n (%) female	152,157 (55.6)	62,293 (58.4)	61,419 (57.6)
**Race**; n (%)			
White	159,028 (58.1)	70,243 (65.9)	70,128 (65.8)
Black or African American	50,329 (18.4)	19,349 (18.2)	18,583 (17.4)
Unknown	54,131 (19.8)	12,565 (11.8)	13,693 (12.8)
**Ethnicity**; n (%)			
Hispanic or Latino	43,254 (15.8)	9,014 (8.5)	8,944 (8.4)
Not Hispanic of Latino	151,246 (55.3)	72,644 (68.2)	72,075 (67.6)
Unknown	79,118 (28.9)	24,920 (23.4)	25,559 (24.0)
**COMORBIDITIES**; n (%)			
Overweight and obesity	50,209 (18.4)	19,080 (17.9)	18,182 (17.1)
Hypertensive disease	83,970 (30.7)	28,188 (26.4)	26,189 (24.6)
Type 2 diabetes mellitus	43,127 (15.8)	12,087 (11.3)	11,254 (10.6)
Asthma	29,556 (10.8)	17,097 (16.0)	16,418 (15.4)
Nicotine dependence	20,091 (7.3)	12,602 (11.8)	12,111 (11.4)
Substance misuse	29,240 (10.7)	16,187 (15.2)	15,446 (14.5)
Mood disorders	42,041 (15.4)	19,933 (18.7)	18,916 (17.7)
Anxiety disorders	52,299 (19.1)	25,731 (24.1)	24,302 (22.8)
Ischemic heart diseases	24,980 (9.1)	7,990 (7.5)	7,350 (6.9)
Other forms of heart disease	49,825 (18.2)	16,688 (15.7)	15,654 (14.7)
CKD	18,455 (6.7)	5,310 (5.0)	5,029 (4.7)
Neoplasms (any)	52,535 (19.2)	20,945 (19.7)	19,474 (18.3)
**OUTCOMES,** % from 1 day to 6 months post-diagnosis (95% CI)			
Anxiety/Depression	22.82 (22.48–23.14)	26.69 (26.14–27.24)	19.79 (19.47–20.11)
Chest/Throat pain	12.60 (12.34–12.86)	12.80 (12.36–13.22)	7.17 (6.97–7.38)
Abnormal breathing	18.71 (18.41–19.02)	18.43 (17.96–18.90)	9.72 (9.48–9.95)
Myalgia	3.24 (3.09–3.38)	3.67 (3.42–3.91)	2.23 (2.12–2.35)
Fatigue	12.82 (12.56–13.09)	12.59 (12.16–13.03)	6.81 (6.60–7.00)
Headache	8.67 (8.44–8.90)	10.53 (10.14–10.90)	7.98 (7.75–8.19)
Abdominal symptoms	15.58 (15.26–15.87)	17.34 (16.84–17.83)	11.42 (11.16–11.66)
Cognitive symptoms	7.88 (7.69–8.08)	5.56 (5.28–5.85)	3.16 (3.02–3.30)
Pain	11.60 (11.33–11.87)	12.09 (11.67–12.54)	8.34 (8.13–8.56)
Any	57.00 (56.59–57.43)	59.37 (58.72–60.00)	42.77 (42.38–43.16)
**OUTCOMES,** % from 3 months to 6 months post-diagnosis (95% CI)			
Anxiety/Depression	15.49 (15.21–15.77)	19.24 (18.59–19.90)	14.27 (13.97–14.57)
Chest/Throat pain	5.71 (5.53–5.90)	6.48 (6.08–6.91)	3.79 (3.63–3.96)
Abnormal breathing	7.94 (7.72–8.16)	9.08 (8.62–9.54)	4.69 (4.51–4.87)
Myalgia	1.54 (1.44–1.64)	2.05 (1.82–2.28)	1.27 (1.17–1.36)
Fatigue	5.87 (5.68–6.06)	6.38 (5.99–6.79)	3.73 (3.58–3.89)
Headache	4.63 (4.47–4.80)	6.66 (6.25–7.07)	5.08 (4.89–5.27)
Abdominal symptoms	8.29 (8.06–8.51)	10.69 (10.16–11.22)	6.84 (6.64–7.06)
Cognitive symptoms	3.95 (3.80–4.10)	3.01 (2.74–3.29)	1.83 (1.71–1.94)
Pain	7.19 (6.98–7.39)	8.53 (8.06–9.00)	5.53 (5.33–5.72)
Any	36.55 (36.16–36.94)	42.34 (41.52–43.19)	29.70 (29.32–30.09)

CKD, chronic kidney disease; COVID-19, Coronavirus Disease 2019.

In the 6 months after COVID-19 diagnosis, 57.00% (95% CI 56.59 to 57.43) had at least one feature of long-COVID recorded. This value includes the incidence of features recorded in the acute as well as the later phase of the illness. In the 90- to 180-day “long” phase post-diagnosis, 36.55% had a long-COVID feature recorded (95% CI 36.18% to 36.94%). The incidence of individual features ranged from 3.24% (95% CI 3.09 to 3.38; including 1.54% in the “long” phase, 95% CI 1.44 to 1.64) for myalgia, to 22.8% (95% CI 22.48 to 23.14; including 15.49% in the “long” phase, 95% CI 15.21 to 15.77) for anxiety/depression.

**Fig 1:**
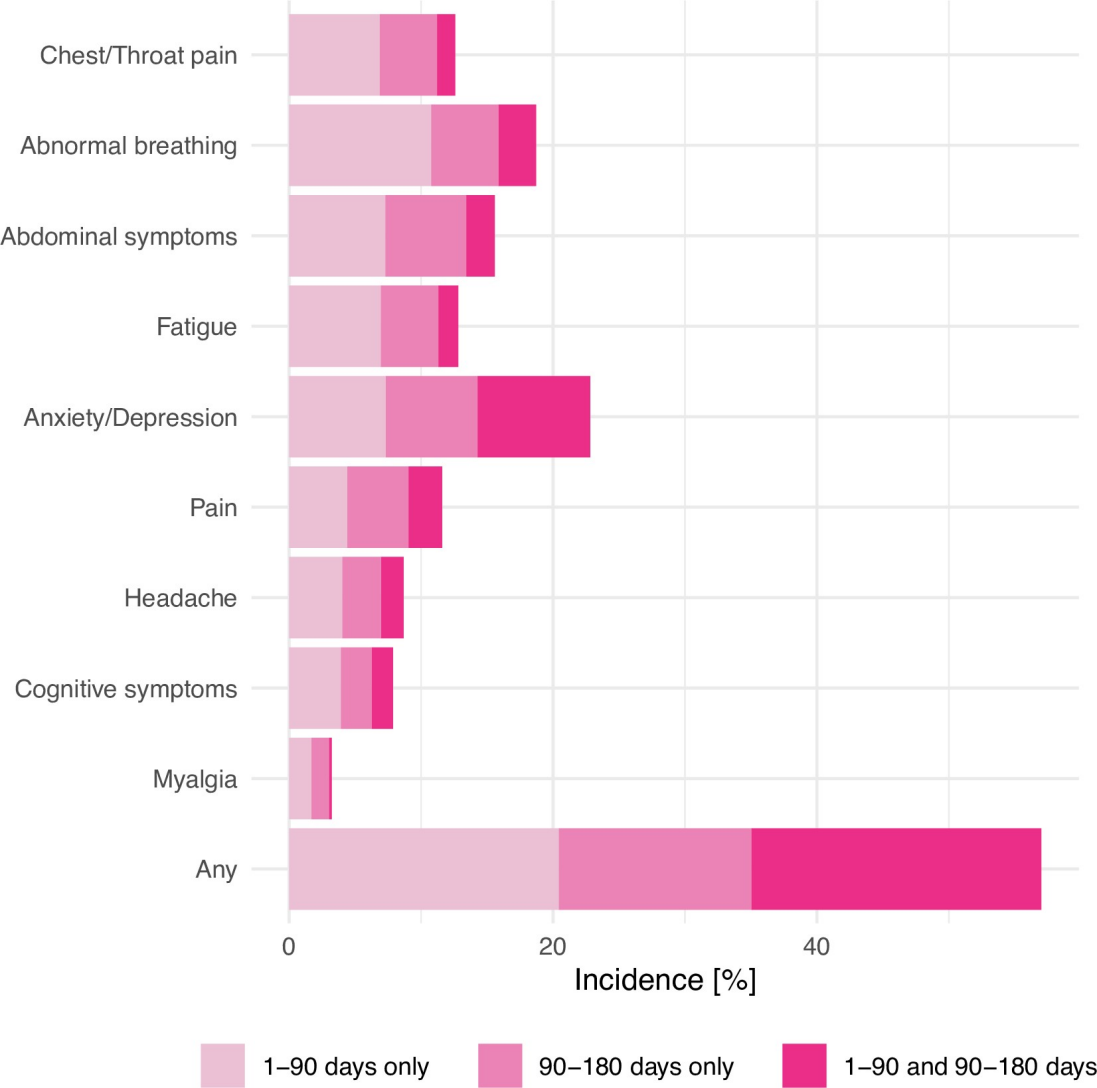
Incidence of each long-COVID feature in the 180 days after COVID-19. The total length of the bars represents the incidence over the entire 1–180-day period. The contributions to this overall incidence are provided in terms of incidence of features that occurred in the 1–90-day period only (i.e., those that did not recur in the 90–180-day period), incidence of features that occurred in the 90–180 days only (i.e., those that had not already occurred in the 1–90-day period), and incidence of features that occurred in the 1–90-day period and recurred in the 90–180-day period. As can be seen by comparing the 2 darker shades of the bottom bar, 60.1% of patients with a feature recorded for the first time in the 90–180 days after diagnosis had at least one feature recorded in the first 90 days. COVID-19, Coronavirus Disease 2019.

In the “long” phase, the rates of first occurrence versus recurrences varied between clinical features (Fig 1). Overall, of patients with long-COVID features recorded between 90 and 180 days, 39.9% had not had any feature recorded in the first 90 days; the remaining 60.1% had at least one long-COVID feature in the first 90 days and developed additional or recurrent features in the next 90 days. The incidence of all features, except pain, was lower in the 90- to 180-day period than in the 1- to 90-day period (Table C in S1 Tables).

**Fig 2:**
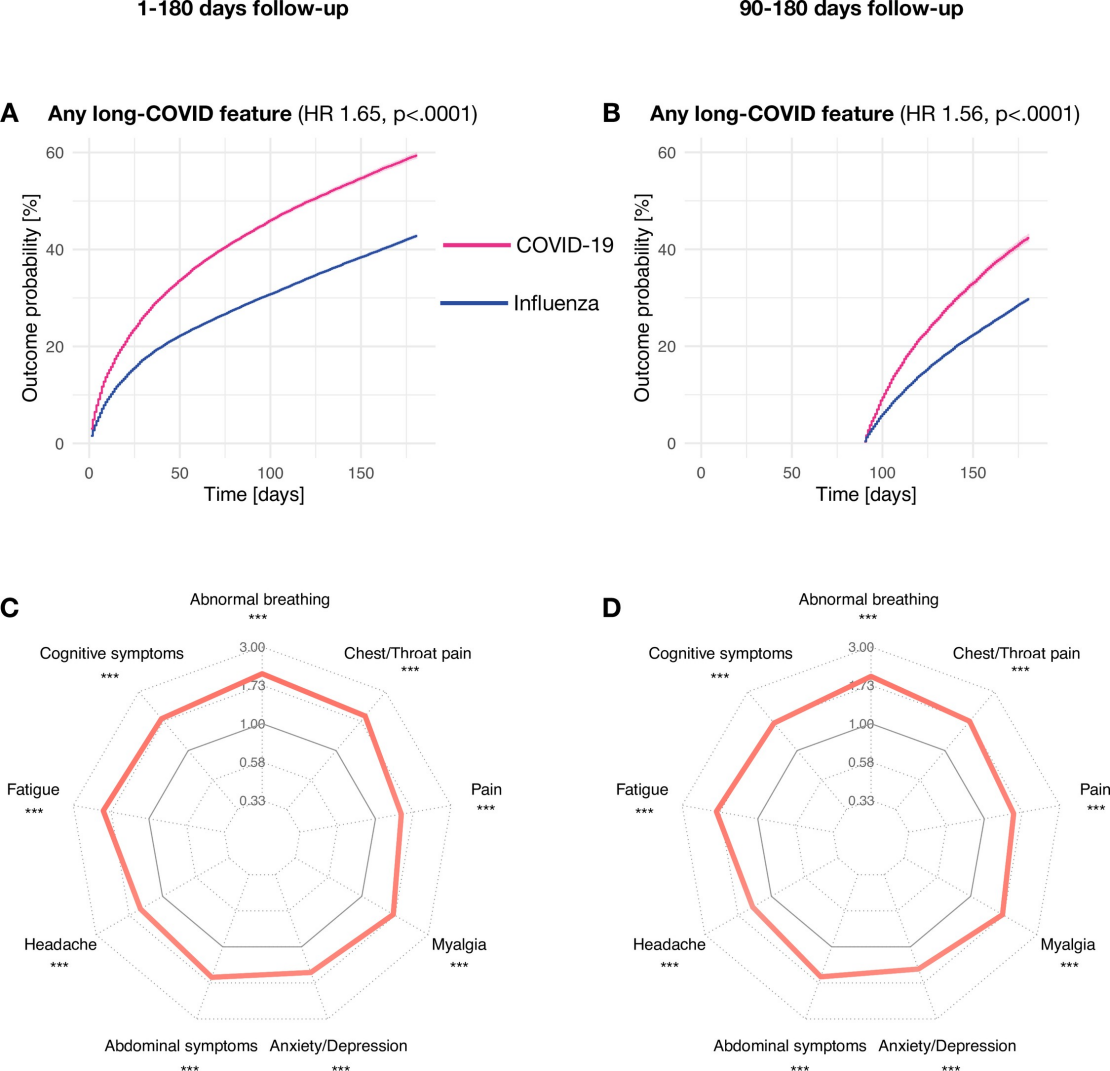
(A, B) Kaplan–Meier curves showing the emergence of long-COVID features over 6 months (A) and specifically over the “long” phase from 3 to 6 months (B) in the cohorts of patients diagnosed with COVID-19 and the matched cohort of patients diagnosed with influenza. (C, D) HRs of individual long-COVID features comparing the cohort of patients with COVID-19 to the matched cohort of patients with influenza. *p < 0.05, **p < 0.01, ***p < 0.001. All long-COVID features are more common after COVID-19 than after influenza. For Kaplan–Meier curves of individual long-COVID features, see Fig A in S1 Fig. COVID-19, Coronavirus Disease 2019; HR, hazard ratio.

As shown in Fig 2 and Fig A in S1 Fig, the incidence of each and any long-COVID feature was significantly higher after COVID-19 than after influenza (overall HR = 1.65 [95% CI 1.62 to 1.67]; individual HRs between 1.44 and 2.04; all *p* < 0.001 for the whole 1- to 180-day period; and overall HR = 1.56; individual HRs between 1.36 and 1.97; all *p* < 0.001 for the “long” phase). Long-COVID features are thus more common after COVID-19, although 42.8% of patients with influenza also had one of these features recorded over the same period (including 29.7% during the “long” phase). In contrast, the HR for atopic dermatitis (negative control) was not statistically significantly different from 1 (HR 1.05, 95% CI 0.91 to 1.23, *p* = 0.51 for the 1- to 180-day period; and HR 1.07, 95% CI 0.87 to 1.32, *p* = 0.52 for the “long” phase). The absolute risk increase of any long-COVID feature after COVID-19 compared to influenza was 16.60% (95% CI: 15.84% to 17.33%) for the whole follow-up window and 12.64% (11.73% to 13.57%) for the “long” phase and varied between 1.43% and 8.71% (0.78% to 4.97% in the “long” phase) for different features (Table D in S1 Tables).

**Fig 3:**
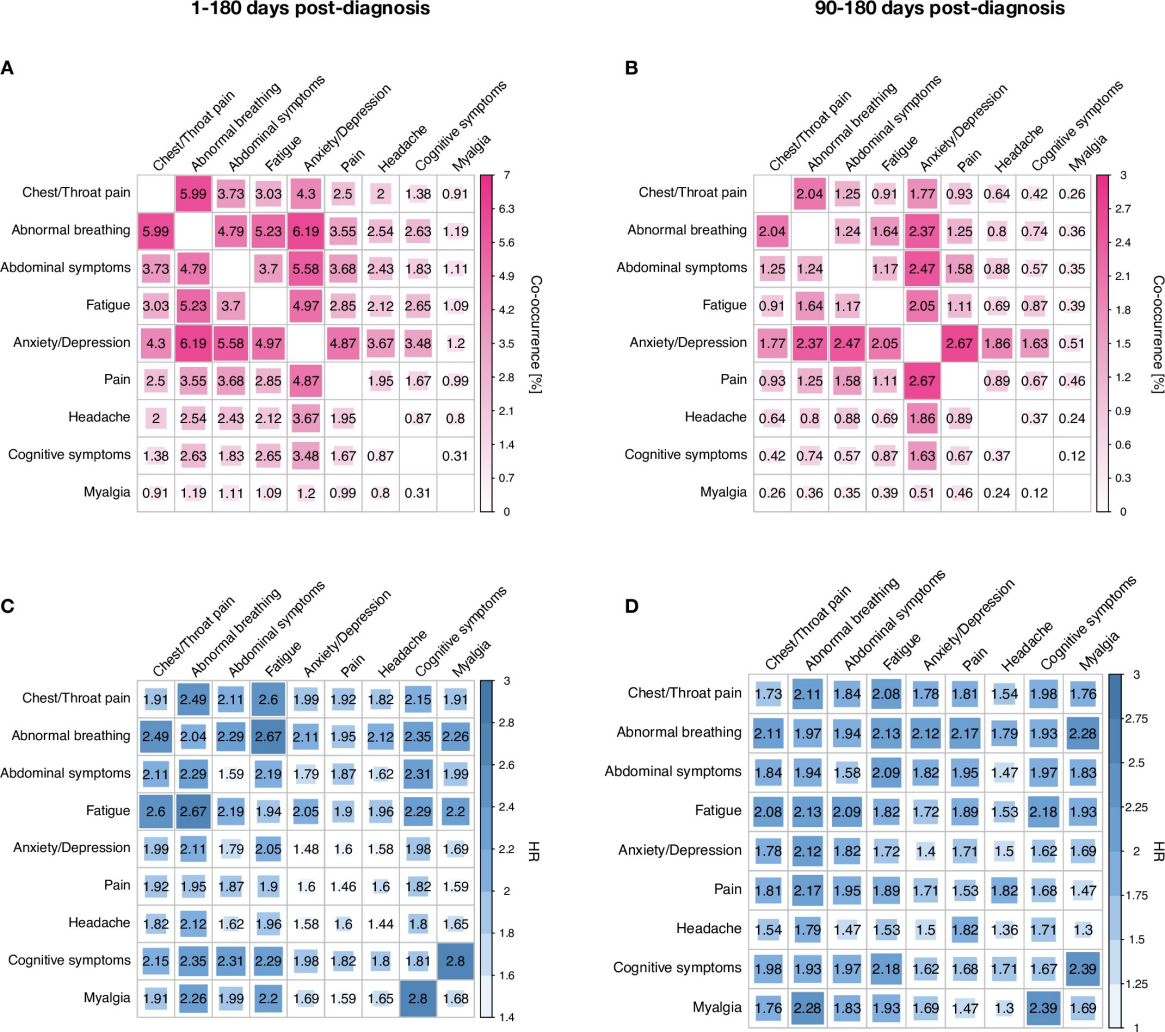
Co-occurrence of pairs of long-COVID symptoms (panels A and B, figures are percentages) and HRs for the co-occurrences relative to a matched cohort with influenza (panels C and D) for the whole 6 months (panels A and C) and the 3–6-month period (panels B and D). Higher values are shown by intensity of pink and blue shading. For example, the co-occurrence of myalgia and cognitive symptoms in the 1–180-day follow-up has a HR of 2.8, whereas the occurrence of each symptom has a HR of 1.68 and 1.81, respectively (see Fig 1). For 95% CIs, see Tables E–H in S1 Tables. CI, confidence interval; HR, hazard ratio.

Fig 3A and 3B show how often pairs of long-COVID features co-occur. For example, 5.99% of patients had both abnormal breathing and chest/throat pain (including 2.04% during the “long” phase). Fig 3C and 3D give the HRs for each co-occurrence compared to influenza. Long-COVID features are seen to co-occur more commonly after COVID-19, as shown by HRs significantly greater than 1, ranging from 1.58 to 2.80 (and from 1.30 to 2.39 for the “long” phase). CIs and *p*-values for all estimates are presented in Tables E-H in S1 Tables.

Most hazards for both incidence and co-occurrence of long-COVID features were found to be proportional, and, when they were not, they remained larger than 1 at 6 months for most outcomes (see Table I in S1 Tables and Figs B and C in S1 Fig).

**Fig 4:**
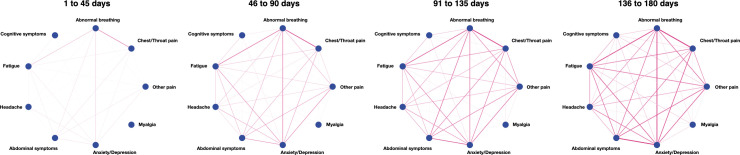
The long-COVID network emerges over the 6-month period, with an increase in the average degree over time. See text for details and Fig E in S1 Fig for a finer grained visualization (10-day intervals).

The clinical feature network was found to be more interconnected post-COVID-19 than following influenza (mean [95% CI] degree: 1.70 [1.54 to 1.87] versus 1.39 [1.26 to 1.53], *p* < 0.001, Fig D in S1 Fig). The network became significantly more interconnected over time (Fig 4 and Fig E in S1 Fig; biweekly change in average degree: 0.13, 95% CI 0.075 to 0.20, *p* < 0.001). During the 90- to 180-day window, the clinical feature network in the COVID-19 cohort was similarly interconnected as in the influenza cohort (see Fig F in S1 Fig and Table J in S1 Tables; *p* = 0.21).

**Fig 5:**
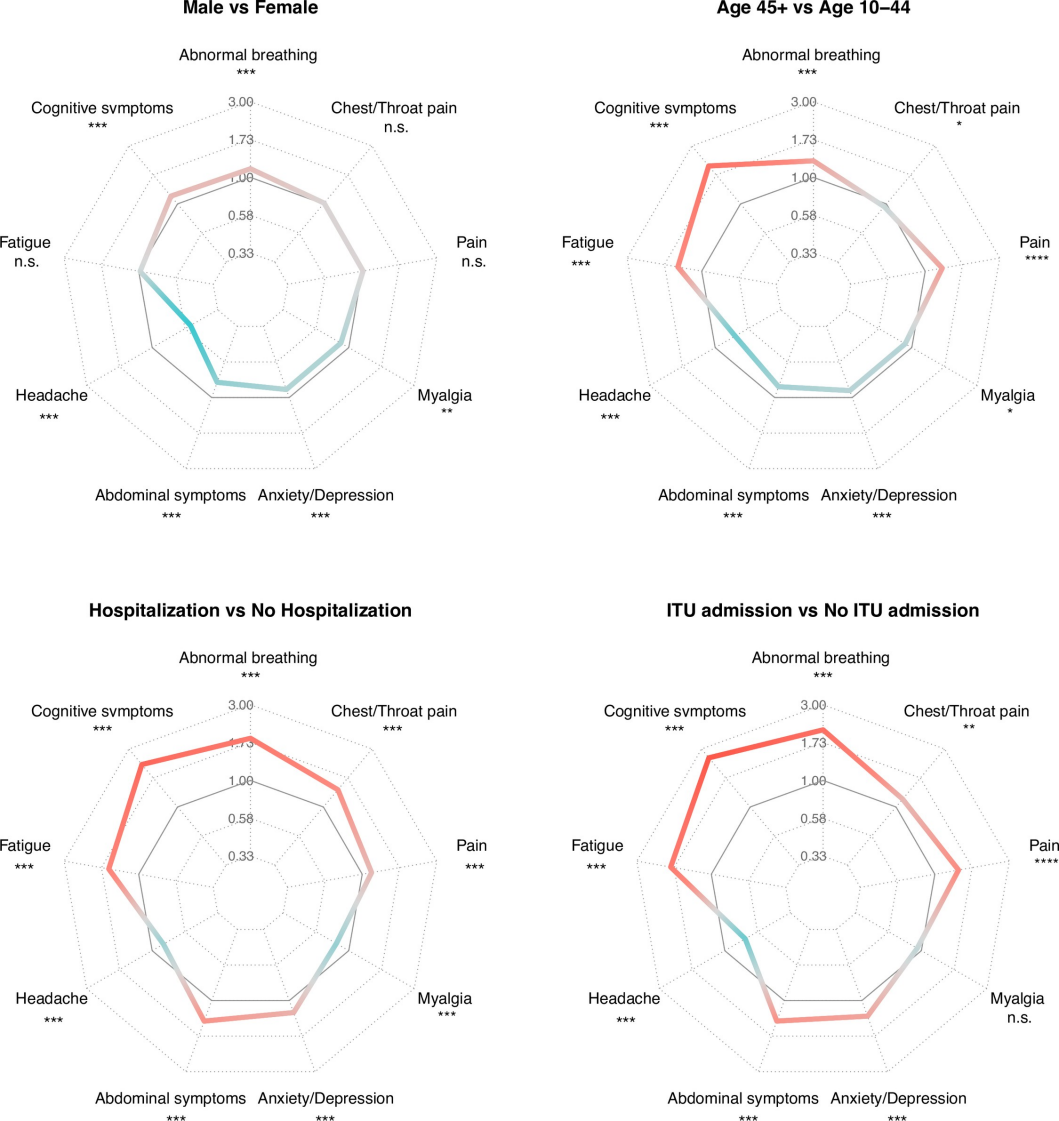
Spider plots summarizing the HRs for each long-COVID feature in subgroups based upon sex, age, and severity of COVID-19 as proxied by requiring hospitalization or ITU admission. HRs are shown comparing the first named group with the second named group. HRs greater than 1 are in red; HRs less than 1 in blue. Significance indicated by asterisks, *p < 0.05, **p < 0.01, ***p < 0.001. Each comparison is based on propensity score–matched cohorts; for baseline characteristics, see Tables M-T in S1 Tables). For spider plots of all subgroup analyses, see Fig AE in S1 Fig. COVID-19, Coronavirus Disease 2019; HR, hazard ratio; ITU, intensive treatment unit.

We next explored how sex, age, race, and indices of COVID-19 severity impacted on long-COVID features (Fig 5 and G-AE in S1 Fig and Tables K-T in S1 Tables). The incidence of “any” long-COVID feature varied from 46.42% in the 10- to 21-year age group, to 61.05% in the over 65s, 63.64% of those hospitalized, and 73.22% of those admitted to ITU. Females were significantly more likely to have headaches, abdominal symptoms, and anxiety/depression, whereas males were significantly more likely to have breathing difficulties and cognitive symptoms. Younger patients were significantly more likely to have headaches, abdominal symptoms, and anxiety/depression, whereas older patients were more likely to have breathing difficulties, cognitive symptoms, pain, and fatigue. Only minor differences were observed between white and non-white patients.

Differences in severity of the COVID-19 illness were also associated with differences in incidence of long-COVID features. Patients with more severe illnesses (as proxied by hospitalization, ITU admission, or leukocytosis) had significantly more features overall, and significantly more cognitive difficulties, but were less likely to have myalgia or headaches. There were no significant differences in the clinical feature networks in any of these subgroup comparisons (Table J in S1 Tables).

Finally, among people who had a given long-COVID feature reported at least once, the total number of recorded occurrences of that feature was significantly larger among patients with COVID-19 than patients with influenza (Table U in S1 Tables): *p* < 0.001 for all clinical features except chest/throat pain (*p* = 0.043), pain (*p* = 0.26), and myalgia (*p* = 0.77).

## Discussion

COVID-19 appears to be associated with long-term effects that are common and diverse, with 57% of patients having at least one long-COVID feature recorded in the 180 days after infection (Table 1 and Fig 1) and 37% having them in the 90 to 180 days after diagnosis, of whom 40% had not had one in the first 3 months. These features are all more common after COVID-19 than after influenza. The fact that most hazards were proportional between the 2 cohorts or that the HRs remained above 1 after 180 days indicates that the risk of long-COVID features occurring, or co-occurring, continues to increase 180 days after the illness and that, for most of them, they follow the same trend as in the acute phase of the illness.

Cognitive symptoms (for definition, see Supporting Methods D in S1 Text) were observed in 7.88% of patients after COVID-19, with markedly higher incidences in the elderly and in those hospitalized or needing ITU admission (Figs V and Y in S1 Fig). “Other symptoms and signs involving cognitive functions and awareness” (ICD-10 code R41) account for 67% of post-COVID cognitive symptoms in the matched COVID-19 cohort, which likely reflects the “brain fog,” word finding difficulties or poor concentration reported in descriptions and surveys of long-COVID. The present findings complement evidence of impaired cognitive performance in a large population of people who self-reported a history of COVID-19 [25]. In total, the data highlight cognition as an important issue after COVID-19, which requires surveillance and investigation.

The long-COVID features involving pain were notable for 3 reasons. First, the overall incidence of pain (of any kind) recorded after COVID-19 was 34.2% (Table V in S1 Tables), higher than any of the other features, and higher than after influenza (24.0%). Second, pain was the only feature that had a higher incidence in the 3- to 6-month period than in the 0- to 3-month period (Table C in S1 Tables). Pain, therefore, appears to be a prominent and relatively persistent element of long-COVID. Third, headache and myalgia had characteristics that differed from the other pain categories: They were more common in women and in younger patients, and notably so in those who had been less acutely ill (as proxied by not requiring hospitalization or ITU admission, and without leukocytosis; Fig 4 and Fig AE in S1 Fig). In each case, this was in the opposite direction to the overall burden of long-COVID features. As such, post-COVID headache and myalgia may result from a different mechanism than the other long-COVID features.

Long-COVID features were observed in all COVID-19 subgroups examined, but the incidence of features differed in relation to demographic and illness severity factors. Overall, there was a higher incidence of long-COVID features in the elderly, in more severely affected patients, and in women. However, it is notable that long-COVID features were also recorded in children and young adults, and in more than half of nonhospitalized patients, confirming that they occur even in young people and those who had a relatively mild illness (Tables K-L in S1 Tables). This is significant in public health terms, given that most people with COVID-19 are in the latter group. It is also of interest that almost 40% of patients with long-COVID symptoms recorded between 3 and 6 months had not had any such diagnosis in the first 3 months. Some of this may reflect a delay in presentation but also suggests that some patients may have a delayed onset of long-COVID features.

Besides high incidences of individual features, long-COVID features co-occurred (Fig 3) and formed a network that emerged over time (Fig 4). The evolution of this network indicates that, compared to the acute phase of the illness, when a long-COVID feature was observed in the later stage of the follow-up, it was more likely to co-occur with other features. This is consistent with the existence of a long-COVID “syndrome,” although this cannot be established by a study of this kind. Similarly, our data cannot identify whether there are subtypes of long-COVID. However, Fig 4 shows that abnormal breathing, chest/throat pain, fatigue, and anxiety/depression are particularly interconnected and form early, whereas myalgia and cognitive symptoms remain only weakly connected. It is therefore possible that the latter features may have a different origin and/or mechanism relative to the other long-COVID features.

The finding that 57% of patients have a long-COVID feature recorded in the 180 days after a diagnosis of COVID-19 should not be misinterpreted as a 57% incidence of long-COVID since this time window includes the acute phase of the illness. However, jointly analyzing the 2 time windows (1 to 180 days and 90 to 180 days) gives insight into the pattern of occurrences and recurrences of long-COVID features throughout the first 180 days post-COVID-19. Specifically, some features (e.g., abnormal breathing) are more common in the first 90 days post-COVID than in the next 90 days, whereas others (e.g., anxiety/depression) are more common in the “long” phase (Fig 1). It can also be seen that fatigue, when it occurs in the “long” phase, tends to be a first occurrence rather than a recurrence, whereas the opposite is true of depression/anxiety. Combined with the finding of a higher number of occurrences of most long-COVID features post-COVID than post-influenza, these results suggest that for some patients with long-COVID, the course of the illness is marked by relapses, in line with results from a recent study based on survey data [9]. However plausible, this conclusion cannot be drawn with confidence from our data since a recurrent record of a long-COVID feature might represent a persistent symptom rather than a relapse. In either case, they show that the burden of most long-COVID features appears greater for patients after COVID-19 than after influenza.

We note several limitations, beyond those inherent to research using EHRs [13,26] (summarized in Supporting Methods A in S1 Text). First, there is an increasingly long list of putative long-COVID features, and we captured only a proportion of them, albeit the majority of those that appear to be commonly described. For example, we did not measure anosmia or hair loss. Second, many patients may have unrecorded long-COVID features, making our incidence figures lower bound estimates. Third, we do not know with any clarity the persistence nor severity of the long-COVID features. Fourth, using an EHR network with both insured and uninsured patients represented provides a higher degree of external generalizability than many previous studies. However, there are limits; many people are likely to have had COVID-19 without it being recorded in their EHR, either because it went undiagnosed or because they were tested at other facilities. In addition, patients who died in the 6 months following their illness were not represented in the data. Fifth, because fewer patients had influenza than COVID-19 during the study period, the 1:1 matching resulted in a matched COVID-19 cohort, which had baseline characteristics similar to the influenza cohort, rather than the other way around. This implies that, strictly speaking, all results comparing the 2 cohorts apply to a population that has the baseline characteristics of the matched COVID-19 cohort, not the whole COVID-19 cohort and the two differ slightly (e.g., the matched cohort is younger than the whole cohort). A final limitation is that, after COVID-19, patients might be more likely to report symptoms, or health professionals more likely to make diagnoses, compared to patients recovering from influenza.

The last limitation deserves further consideration for it might artificially increase the HR between cohorts. But 2 factors suggest that any effect is likely to be limited. First, the 2 cohorts were diagnosed with atopic dermatitis (a negative control) at the same rate over the same time period. Second, at the time our data collection ended (December 2020), long-COVID was far less in the public consciousness than it is now (see Fig AF in S1 Fig) and thus less likely to have been a major factor influencing presentations with symptoms.

The data presented here shed light on the incidences and relative risks of long-COVID features, but say nothing about the causation or mechanisms involved [27–29], nor about the predictors beyond the limited demographic and severity markers we measured. Research aimed at these issues is required. For example, to what extent do preexisting health conditions or characteristics, or specific features of the acute infection, impact on long-COVID? Finally, we note that almost 43% of patients after influenza had at least one long-COVID feature recorded (Table 1) including 29.7% during the 90- to 180-day period. In this regard, we suggest researchers take a broad and balanced view as to the nature and specificity of long-COVID. In the meantime, clinical services need to be prepared and resourced to enable management of long-COVID features according to the best available evidence as it emerges [7,30,31].

## Disclaimer

The views expressed are those of the authors and not necessarily those of the UK National Health Service, NIHR, or the UK Department of Health.

## Supporting information

S1 STROBE Checklist The STROBE Checklist for this manuscript. (DOCX)

S1 Figures Supporting Figures. **Fig A.** Kaplan–Meier curves showing the emergence of long-COVID features over 6 months. All symptoms of long-COVID are more common after COVID-19 (pink) than after influenza (blue). COVID-19 and influenza cohorts are propensity score matched for known COVID-19 risk factors. Shaded areas around curves represent the 95% CI. **Fig B**. Time-varying HRs for the 12 outcomes (out of 46) for which there was evidence of nonproportionality of hazards in the main comparison between the cohort of patients with COVID-19 and a matched cohort of patients with influenza. Shaded area represents a 95% CI. **Fig C**. Time-varying HRs for the 11 outcomes (out of 46) for which there was evidence of nonproportionality of hazards in the comparison between the cohort of patients with COVID-19 and a matched cohort of patients with influenza when the time window for follow-up is set to 3–6 months. Note that the x-axis is shifted on these figures so that day 1 corresponds to 3 months post-index event. Shaded area represents a 95% CI. **Fig D**. Using Dice’s coefficients and permutation testing, the long-COVID symptom network is seen to be more interconnected in the 6 months after COVID-19 than after influenza, after controlling for the incidence of each symptom in the network (i.e., the normalized co-occurrence). The top panels represent the network as a graph with edges weighted by the values of Dice’s coefficient. The bottom panels provide the actual values of Dice’s coefficients for each pair of features. **Fig E**. Similar figure as Fig 4 of the main manuscript representing the evolution of the clinical feature network over time, with higher temporal granularity. **Fig F**. Using Dice’s coefficients and permutation testing, the long-COVID symptom network is seen to not be more interconnected in the 3 to 6 months after COVID-19 than after influenza, after controlling for the incidence of each symptom in the network (i.e., the normalized co-occurrence). The top panels represent the network as a graph with edges weighted by the values of Dice’s coefficient. The bottom panels provide the actual values of Dice’s coefficients for each pair of features. **Fig G**. Kaplan–Meier curves for the comparison of the incidence of each and any clinical feature of long-COVID in the 6 months after a diagnosis of COVID-19 comparing matched cohorts of females (pink curves) vs. males (blue curves). Shaded areas represent 95% CIs. **Fig H**. HR for the individual incidence (diagonal) and the co-occurrence (off-diagonal) of clinical features of long-COVID after a diagnosis of COVID-19 comparing matched cohorts of females vs. males (values higher than 1 indicate a significantly higher risk among females). Higher values are shown by intensity of red and blue shading. Only HR reaching significance at *p* < 0.05 are displayed, and the corresponding *p*-values are presented on the right panel (**** *p* < 0.0001, *** *p* < 0.001, ** *p* < 0.01, * *p* < 0.05). **Fig I**. Clinical feature networks after a diagnosis of COVID-19 among matched cohorts of females and males. Dice’s coefficients graphically represented as the edges of the networks in the top panels are reported numerically in the bottom panels. **Fig J**. Kaplan–Meier curves for the comparison of the incidence of each and any clinical feature of long-COVID in the 6 months after a diagnosis of COVID-19 comparing matched cohorts of non-white (pink curves) vs. white (blue curves). Shaded areas represent 95% CIs. **Fig K**. HR for the individual incidence (diagonal) and the co-occurrence (off-diagonal) of clinical features of long-COVID after a diagnosis of COVID-19 comparing matched cohorts of non-white vs. white (values higher than 1 indicate a significantly higher risk among non-white patients). Higher values are shown by intensity of red and blue shading. Only HR reaching significance at *p* < 0.05 are displayed, and the corresponding *p*-values are presented on the right panel (**** *p* < 0.0001, *** *p* < 0.001, ** *p* < 0.01, * *p* < 0.05). **Fig L**. Clinical feature networks after a diagnosis of COVID-19 among matched cohorts of non-white and white patients. Dice’s coefficients graphically represented as the edges of the networks in the top panels are reported numerically in the bottom panels. **Fig M**. Kaplan–Meier curves for the comparison of the incidence of each and any clinical feature of long-COVID in the 6 months after a diagnosis of COVID-19 comparing matched cohorts of patients age 45 and over (pink curves) vs. age 10–44 (blue curves). Shaded areas represent 95% CIs. **Fig N**. HR for the individual incidence (diagonal) and the co-occurrence (off-diagonal) of clinical features of long-COVID after a diagnosis of COVID-19 comparing matched cohorts of patients age 45 and over vs. patients age 10–44 (values higher than 1 indicate a significantly higher risk among those age 45 and over). Higher values are shown by intensity of red and blue shading. Only HR reaching significance at *p* < 0.05 are displayed, and the corresponding *p*-values are presented on the right panel (**** *p* < 0.0001, *** *p* < 0.001, ** *p* < 0.01, * *p* < 0.05). **Fig O**. Clinical feature networks after a diagnosis of COVID-19 among matched cohorts of patients age 45 and over vs. patients age 10–44. Dice’s coefficients graphically represented as the edges of the networks in the top panels are reported numerically in the bottom panels. **Fig P**. Kaplan–Meier curves for the comparison of the incidence of each and any clinical feature of long-COVID in the 6 months after a diagnosis of COVID-19 comparing matched cohorts of patients age 65 and over (pink curves) vs. age 45–64 (blue curves). Shaded areas represent 95% CIs. **Fig Q**. HR for the individual incidence (diagonal) and the co-occurrence (off-diagonal) of clinical features of long-COVID after a diagnosis of COVID-19 comparing matched cohorts of patients age 65 and over vs. patients age 45–64 (values higher than 1 indicate a significantly higher risk among those age 65 and over). Higher values are shown by intensity of red and blue shading. Only HR reaching significance at *p* < 0.05 are displayed, and the corresponding *p*-values are presented on the right panel (**** *p* < 0.0001, *** *p* < 0.001, ** *p* < 0.01, * *p* < 0.05). **Fig R**. Clinical feature networks after a diagnosis of COVID-19 among matched cohorts of patients age 65 and over vs. patients age 45–64. Dice’s coefficients graphically represented as the edges of the networks in the top panels are reported numerically in the bottom panels. **Fig S**. Kaplan–Meier curves for the comparison of the incidence of each and any clinical feature of long-COVID in the 6 months after a diagnosis of COVID-19 comparing matched cohorts of patients age 22–44 (pink curves) vs. age 10–21 (blue curves). Shaded areas represent 95% CIs. **Fig T**. HR for the individual incidence (diagonal) and the co-occurrence (off-diagonal) of clinical features of long-COVID after a diagnosis of COVID-19 comparing matched cohorts of patients age 22–44 vs. patients age 10–21 (values higher than 1 indicate a significantly higher risk among those age 22–44). Higher values are shown by intensity of red and blue shading. Only HR reaching significance at *p* < 0.05 are displayed, and the corresponding *p*-values are presented on the right panel (**** *p* < 0.0001, *** *p* < 0.001, ** *p* < 0.01, * *p* < 0.05). **Fig U.** Clinical feature networks after a diagnosis of COVID-19 among matched cohorts of patients age 22–44 vs. patients age 10–21. Dice’s coefficients graphically represented as the edges of the networks in the top panels are reported numerically in the bottom panels. **Fig V.** Kaplan–Meier curves for the comparison of the incidence of each and any clinical feature of long-COVID in the 6 months after a diagnosis of COVID-19 comparing matched cohorts of patients requiring (pink curves) vs. not requiring hospitalization (blue curves). Shaded areas represent 95% CIs. **Fig W**. HR for the individual incidence (diagonal) and the co-occurrence (off-diagonal) of clinical features of long-COVID after a diagnosis of COVID-19 comparing matched cohorts of patients requiring vs. not requiring hospitalization (values higher than 1 indicate a significantly higher risk among those requiring hospitalization). Higher values are shown by intensity of red and blue shading. Only HR reaching significance at *p* < 0.05 are displayed, and the corresponding *p*-values are presented on the right panel (**** *p* < 0.0001, *** *p* < 0.001, ** *p* < 0.01, * *p* < 0.05). **Fig X.** Clinical feature networks after a diagnosis of COVID-19 among matched cohorts of patients requiring vs. not requiring hospitalization. Dice’s coefficients graphically represented as the edges of the networks in the top panels are reported numerically in the bottom panels. **Fig Y.** Kaplan–Meier curves for the comparison of the incidence of each and any clinical feature of long-COVID in the 6 months after a diagnosis of COVID-19 comparing matched cohorts of patients requiring (pink curves) vs. not requiring ITU admission (blue curves). Shaded areas represent 95% CIs. **Fig Z.** HR for the individual incidence (diagonal) and the co-occurrence (off-diagonal) of clinical features of long-COVID after a diagnosis of COVID-19 comparing matched cohorts of patients requiring vs. not requiring ITU admission (values higher than 1 indicate a significantly higher risk among those requiring ITU admission). Higher values are shown by intensity of red and blue shading. Only HR reaching significance at *p* < 0.05 are displayed, and the corresponding *p*-values are presented on the right panel (**** *p* < 0.0001, *** *p* < 0.001, ** *p* < 0.01, * *p* < 0.05). **Fig AA.** Clinical feature networks after a diagnosis of COVID-19 among matched cohorts of patients requiring vs. not requiring ITU admission. Dice’s coefficients graphically represented as the edges of the networks in the top panels are reported numerically in the bottom panels. **Fig AB.** Kaplan–Meier curves for the comparison of the incidence of each and any clinical feature of long-COVID in the 6 months after a diagnosis of COVID-19 comparing matched cohorts of patients with (pink curves) vs. without leukocytosis (blue curves). Shaded areas represent 95% CIs. **Fig AC**. HR for the individual incidence (diagonal) and the co-occurrence (off-diagonal) of clinical features of long-COVID after a diagnosis of COVID-19 comparing matched cohorts of patients with vs. without leukocytosis (values higher than 1 indicate a significantly higher risk among those with leukocytosis). Higher values are shown by intensity of red and blue shading. Only HR reaching significance at *p* < 0.05 are displayed, and the corresponding *p*-values are presented on the right panel (**** *p* < 0.0001, *** *p* < 0.001, ** *p* < 0.01, * *p* < 0.05). **Fig AD**. Clinical feature networks after a diagnosis of COVID-19 among matched cohorts of patients with vs. without leukocytosis. Dice’s coefficients graphically represented as the edges of the networks in the top panels are reported numerically in the bottom panels. **Fig AE**. HRs comparing the incidence of clinical features of long-COVID between matched subgroups of patients diagnosed with COVID-19. The figure includes the spider plots shown in Fig 5 of the main manuscript. For each comparison of “Group A vs Group B”, a HR larger than 1 indicates that the incidence is higher in Group A (and vice versa for a HR lower than 1). Whether the HR is statistically significant is indicated with a star code underneath each feature: **** *p* < 0.0001, *** *p* < 0.001, ** *p* < 0.01, * *p* < 0.05, n.s. *p* > 0.05. **Fig AF.** Number of references on medRxiv containing the term “long-COVID” in 2-month interval since the beginning of the pandemic. This shows that the study (whose follow-up ended on December 16, 2020) largely took place at a time where public awareness long-COVID was significantly less than now. This suggests that public awareness alone is unlikely to have led to substantially more patients seeking medical attention for otherwise equal symptoms as the control cohort. CI, confidence interval; COVID-19, Coronavirus Disease 2019; HR, hazard ratio; ITU, intensive treatment unit.
(DOCX)

S1 Tables Supporting Tables. **Table A.** Characteristics of the unmatched COVID-19 cohort and the matched COVID-19 and influenza cohorts. **Table B.** Contributions of incidence (within 6 months of a diagnosis of COVID-19 vs. influenza) of subcategories making up the clinical features of long-COVID in matched cohorts. **Table C**. Incidence of long-COVID features in the whole cohort of patients with COVID-19 within the entire follow-up period (0–6 months), the first half of the follow-up period (0–3 months), and the second half of the follow-up period (3–6 months). In the analysis of the 3–6-month follow-up, those who had the long-COVID feature recorded in the first 3 months and then again in the next 3 months were included so that the sum of the incidences in the two-halves of the follow-up window exceeds the total incidence. **Table D**. Absolute risk increase in COVID-19 vs. influenza (a positive number indicates a higher risk in COVID-19) in the whole 0–6-month period as well as the “long” phase (3–6 months). **Table E.** 95% CIs corresponding to the entries in Fig 3A of the main manuscript, i.e., for the incidence (on the diagonal) and co-occurrence (off-diagonal) of long-COVID features in the 6 months after a diagnosis of COVID-19. **Table F.** 95% CIs corresponding to the entries in Fig 3B of the main manuscript, i.e., for the incidence (on the diagonal) and co-occurrence (off-diagonal) of long-COVID features in the period extending from 3 to 6 months after a diagnosis of COVID-19. **Table G.** 95% CIs corresponding to the entries in Fig 3C of the main manuscript, i.e., for the HRs of the incidence (on the diagonal) and co-occurrence (off-diagonal) of long-COVID features in the 6 months after a diagnosis of COVID-19 vs. influenza. All corresponding *p*-values were <0.0001 except for the co-occurrence of cognitive symptoms and myalgia (*p* = 0.0007). **Table H.** 95% CIs corresponding to the entries in Fig 3D of the main manuscript, i.e., for the HRs of the incidence (on the diagonal) and co-occurrence (off-diagonal) of long-COVID features in the period extending from 3 to 6 months after a diagnosis of COVID-19 vs. influenza. All corresponding *p*-values were <0.01 except for the co-occurrence of myalgia and headache (*p* = 0.1476), myalgia and cognitive symptoms (*p* = 0.1292), and myalgia and pain (*p* = 0.0139). **Table I**. *p*-values for the test of proportional hazards (obtained using the generalized Schoenfeld test) for the main analysis (1 day to 6 months follow-up) and the analysis restricted to the 3 months–6 months follow-up. A value lower than 0.05 indicates evidence for nonproportional hazards. **Table J.** Average degrees of the clinical feature networks in the different comparisons between cohorts. *p*-values were obtained using permutation tests. **Table K.** 6-month incidence of individual long-COVID features and of any feature in different subgroups of patients (defined by sex, race, or age) diagnosed with COVID-19. **Table L.** 6-month incidence of individual long-COVID features and of any feature in different subgroups of patients defined by indices of severity of COVID-19 illness. **Table M.** Characteristics of the female and male COVID-19 cohorts after propensity score matching**. Table N.** Characteristics of the non-white and white COVID-19 cohorts after propensity score matching**. Table O.** Characteristics of the age 45+ and age 10–44 COVID-19 cohorts after propensity score matching**. Table P.** Characteristics of the age 65+ and age 45–64 COVID-19 cohorts after propensity score matching**. Table Q.** Characteristics of the age 22–44 and age 10–21 COVID-19 cohorts after propensity score matching**. Table R.** Characteristics of COVID-19 cohorts requiring and not requiring hospitalization, after propensity score matching**. Table S.** Characteristics of COVID-19 cohorts requiring and not requiring ITU admission, after propensity score matching**. Table T.** Characteristics of leukocytosis and non-leukocytosis COVID-19 cohorts after propensity score matching**. Table U.** Mean count number of occurrences of each and any long-COVID feature among patients who have them recorded at least once, in the 6 months after a diagnosis of COVID-19 or influenza (using matched cohorts). The *p*-value tests the hypothesis that the counts are equal between the cohorts. **Table V.** Comparison in the 6-month incidence of any pain, between patients with COVID-19 and a matched cohort of patients with influenza. Any pain in this analysis refers to the composite endpoint of chest/throat pain, headache, myalgia, other pain (as defined in Supporting Methods D) or abdominal and pelvic pain (a subcategory of the abdominal symptoms also defined in Supporting Methods D). CI, confidence interval; COVID-19, Coronavirus Disease 2019; HR, hazard ratio; ITU, intensive treatment unit; SMD, standardized mean difference.
(DOCX)

S1 Text Supporting Methods. **Supporting Methods A.** TriNetX network. **Supporting Methods B.** Definition of cohorts. **Supporting Methods C.** Definition of covariates. **Supporting Methods D.** Definition of outcomes. **Supporting Methods E.** Details on statistical analyses. **Supporting Methods F.** Details on secondary analyses.
(DOCX)

## Abbreviations

CI: confidence interval
COVID-19: Coronavirus Disease 2019
EHR: electronic health record
HCO: healthcare organization
HR: hazard ratio
ITU: intensive treatment unit
SMD: standardized mean difference
