# Supplementary material for: Incidence, co-occurrence, and evolution of long-COVID features: A 6-month retrospective cohort study of 273,618 survivors of COVID-19
Source: PLoS Med. 2021 Sep 28;18(9):e1003773. doi: 10.1371/journal.pmed.1003773 (PMC8478214; doi:10.1371/journal.pmed.1003773)
Supplement: S1 Figures — Fig A. Kaplan–Meier curves showing the emergence of long-COVID features over 6 months. All symptoms of long-COVID are more common after COVID-19 (pink) than after influenza (blue). COVID-19 and influenza cohorts are propensity score matched for known COVID-19 risk factors. Shaded areas around curves represent the 95% CI. Fig B. Time-varying HRs for the 12 outcomes (out of 46) for which there was evidence of nonproportionality of hazards in the main comparison between the cohort of patients with COVID-19 and a matched cohort of patients with influenza. Shaded area represents a 95% CI. Fig C. Time-varying HRs for the 11 outcomes (out of 46) for which there was evidence of nonproportionality of hazards in the comparison between the cohort of patients with COVID-19 and a matched cohort of patients with influenza when the time window for follow-up is set to 3–6 months. Note that the x-axis is shifted on these figures so that day 1 corresponds to 3 months post-index event. Shaded area represents a 95% CI. Fig D. Using Dice’s coefficients and permutation testing, the long-COVID symptom network is seen to be more interconnected in the 6 months after COVID-19 than after influenza, after controlling for the incidence of each symptom in the network (i.e., the normalized co-occurrence). The top panels represent the network as a graph with edges weighted by the values of Dice’s coefficient. The bottom panels provide the actual values of Dice’s coefficients for each pair of features. Fig E. Similar figure as Fig 4 of the main manuscript representing the evolution of the clinical feature network over time, with higher temporal granularity. Fig F. Using Dice’s coefficients and permutation testing, the long-COVID symptom network is seen to not be more interconnected in the 3 to 6 months after COVID-19 than after influenza, after controlling for the incidence of each symptom in the network (i.e., the normalized co-occurrence). The top panels represent the network as a graph with edge [file pmed.1003773.s002.docx]

# Supplementary figures

**Fig A** – Kaplan-Meier curves showing the emergence of long-COVID features over 6 months. All symptoms of long-COVID are more common after COVID-19 (pink) than after influenza (blue). COVID-19 and influenza cohorts are propensity score-matched for known COVID-19 risk factors. Shaded areas around curves represent the 95% CI.

**Fig B** – Time-varying hazard ratios for the 12 outcomes (out of 46) for which there was evidence of non-proportionality of hazards in the main comparison between the cohort of patients with COVID-19 and a matched cohort of patients with influenza. Shaded area represents a 95% confidence interval

**Fig C** – Time-varying hazard ratios for the 11 outcomes (out of 46) for which there was evidence of non-proportionality of hazards in the comparison between the cohort of patients with COVID-19 and a matched cohort of patients with influenza when the time window for follow-up is set to 3-6 months. Note that the x-axis is shifted on these figures so that day 1 corresponds to 3 months post index event. Shaded area represents a 95% confidence interval

**Fig D** – Using Dice’s coefficients and permutation testing, the long-COVID symptom network is seen to be more interconnected in the 6 months after COVID-19 than after influenza, after controlling for the incidence of each symptom in the network (i.e. the normalized co-occurrence). The top panels represent the network as a graph with edges weighted by the values of Dice’s coefficient. The bottom panels provide the actual values of Dice’s coefficients for each pair of features.

**Fig E** – Similar figure as Figure 4 of the main manuscript representing the evolution of the clinical feature network over time, with higher temporal granularity.

**Fig F** – Using Dice’s coefficients and permutation testing, the long-COVID symptom network is seen to not be more interconnected in the 3 to 6 months after COVID-19 than after influenza, after controlling for the incidence of each symptom in the network (i.e. the normalized co-occurrence). The top panels represent the network as a graph with edges weighted by the values of Dice’s coefficient. The bottom panels provide the actual values of Dice’s coefficients for each pair of features.

**Fig G** – Kaplan-Meier curves for the comparison of the incidence of each and any clinical feature of long-COVID in the 6 months after a diagnosis of COVID-19 comparing matched cohorts of females (pink curves) vs. males (blue curves). Shaded areas represent 95% confidence intervals.

**Fig H** – Hazard ratio for the individual incidence (diagonal) and the co-occurrence (off-diagonal) of clinical features of long-COVID after a diagnosis of COVID-19 comparing matched cohorts of females vs. males (values higher than 1 indicate a significantly higher risk among females). Higher values are shown by intensity of red and blue shading. Only HR reaching significance at p<0.05 are displayed and the corresponding p-values are presented on the right panel (^****^ p<0.0001, ^***^ p<0.001, ^**^ p<0.01, ^*^ p<0.05).

**Fig I** – Clinical feature networks after a diagnosis of COVID-19 among matched cohorts of females and males. Dice’s coefficients graphically represented as the edges of the networks in the top panels are reported numerically in the bottom panels.

**Fig J** – Kaplan-Meier curves for the comparison of the incidence of each and any clinical feature of long-COVID in the 6 months after a diagnosis of COVID-19 comparing matched cohorts of non-white (pink curves) vs. white (blue curves). Shaded areas represent 95% confidence intervals.

**Fig K** – Hazard ratio for the individual incidence (diagonal) and the co-occurrence (off-diagonal) of clinical features of long-COVID after a diagnosis of COVID-19 comparing matched cohorts of non-white vs. white (values higher than 1 indicate a significantly higher risk among non-white patients). Higher values are shown by intensity of red and blue shading. Only HR reaching significance at p<0.05 are displayed and the corresponding p-values are presented on the right panel (^****^ p<0.0001, ^***^ p<0.001, ^**^ p<0.01, ^*^ p<0.05).

**Fig L** – Clinical feature networks after a diagnosis of COVID-19 among matched cohorts of non-white and white patients. Dice’s coefficients graphically represented as the edges of the networks in the top panels are reported numerically in the bottom panels.

**Fig M** – Kaplan-Meier curves for the comparison of the incidence of each and any clinical feature of long-COVID in the 6 months after a diagnosis of COVID-19 comparing matched cohorts of patients age 45 and over (pink curves) vs. age 10-44 (blue curves). Shaded areas represent 95% confidence intervals.

**Fig N** – Hazard ratio for the individual incidence (diagonal) and the co-occurrence (off-diagonal) of clinical features of long-COVID after a diagnosis of COVID-19 comparing matched cohorts of patients age 45 and over vs. patients age 10-44 (values higher than 1 indicate a significantly higher risk among those age 45 and over). Higher values are shown by intensity of red and blue shading. Only HR reaching significance at p<0.05 are displayed and the corresponding p-values are presented on the right panel (^****^ p<0.0001, ^***^ p<0.001, ^**^ p<0.01, ^*^ p<0.05).

**Fig O** – Clinical feature networks after a diagnosis of COVID-19 among matched cohorts of patients age 45 and over vs. patients age 10-44. Dice’s coefficients graphically represented as the edges of the networks in the top panels are reported numerically in the bottom panels.

**Fig P** – Kaplan-Meier curves for the comparison of the incidence of each and any clinical feature of long-COVID in the 6 months after a diagnosis of COVID-19 comparing matched cohorts of patients age 65 and over (pink curves) vs. age 45-64 (blue curves). Shaded areas represent 95% confidence intervals.

**Fig Q** – Hazard ratio for the individual incidence (diagonal) and the co-occurrence (off-diagonal) of clinical features of long-COVID after a diagnosis of COVID-19 comparing matched cohorts of patients age 65 and over vs. patients age 45-64 (values higher than 1 indicate a significantly higher risk among those age 65 and over). Higher values are shown by intensity of red and blue shading. Only HR reaching significance at p<0.05 are displayed and the corresponding p-values are presented on the right panel (^****^ p<0.0001, ^***^ p<0.001, ^**^ p<0.01, ^*^ p<0.05).

**Fig R** – Clinical feature networks after a diagnosis of COVID-19 among matched cohorts of patients age 65 and over vs. patients age 45-64. Dice’s coefficients graphically represented as the edges of the networks in the top panels are reported numerically in the bottom panels.

**Fig S** – Kaplan-Meier curves for the comparison of the incidence of each and any clinical feature of long-COVID in the 6 months after a diagnosis of COVID-19 comparing matched cohorts of patients age 22-44 (pink curves) vs. age 10-21 (blue curves). Shaded areas represent 95% confidence intervals.

**Fig T** – Hazard ratio for the individual incidence (diagonal) and the co-occurrence (off-diagonal) of clinical features of long-COVID after a diagnosis of COVID-19 comparing matched cohorts of patients age 22-44 vs. patients age 10-21 (values higher than 1 indicate a significantly higher risk among those age 22-44). Higher values are shown by intensity of red and blue shading. Only HR reaching significance at p<0.05 are displayed and the corresponding p-values are presented on the right panel (^****^ p<0.0001, ^***^ p<0.001, ^**^ p<0.01, ^*^ p<0.05).

**Fig U** – Clinical feature networks after a diagnosis of COVID-19 among matched cohorts of patients age 22-44 vs. patients age 10-21. Dice’s coefficients graphically represented as the edges of the networks in the top panels are reported numerically in the bottom panels.

**Fig V** – Kaplan-Meier curves for the comparison of the incidence of each and any clinical feature of long-COVID in the 6 months after a diagnosis of COVID-19 comparing matched cohorts of patients requiring (pink curves) vs. not requiring hospitalisation (blue curves). Shaded areas represent 95% confidence intervals.

**Fig W** – Hazard ratio for the individual incidence (diagonal) and the co-occurrence (off-diagonal) of clinical features of long-COVID after a diagnosis of COVID-19 comparing matched cohorts of patients requiring vs. not requiring hospitalisation (values higher than 1 indicate a significantly higher risk among those requiring hospitalisation). Higher values are shown by intensity of red and blue shading. Only HR reaching significance at p<0.05 are displayed and the corresponding p-values are presented on the right panel (^****^ p<0.0001, ^***^ p<0.001, ^**^ p<0.01, ^*^ p<0.05).

**Fig X** – Clinical feature networks after a diagnosis of COVID-19 among matched cohorts of patients requiring vs. not requiring hospitalisation. Dice’s coefficients graphically represented as the edges of the networks in the top panels are reported numerically in the bottom panels.

**Fig Y** – Kaplan-Meier curves for the comparison of the incidence of each and any clinical feature of long-COVID in the 6 months after a diagnosis of COVID-19 comparing matched cohorts of patients requiring (pink curves) vs. not requiring ITU admission (blue curves). Shaded areas represent 95% confidence intervals.

**Fig Z** – Hazard ratio for the individual incidence (diagonal) and the co-occurrence (off-diagonal) of clinical features of long-COVID after a diagnosis of COVID-19 comparing matched cohorts of patients requiring vs. not requiring ITU admission (values higher than 1 indicate a significantly higher risk among those requiring ITU admission). Higher values are shown by intensity of red and blue shading. Only HR reaching significance at p<0.05 are displayed and the corresponding p-values are presented on the right panel (^****^ p<0.0001, ^***^ p<0.001, ^**^ p<0.01, ^*^ p<0.05).

**Fig AA** – Clinical feature networks after a diagnosis of COVID-19 among matched cohorts of patients requiring vs. not requiring ITU admission. Dice’s coefficients graphically represented as the edges of the networks in the top panels are reported numerically in the bottom panels.

**Fig AB** – Kaplan-Meier curves for the comparison of the incidence of each and any clinical feature of long-COVID in the 6 months after a diagnosis of COVID-19 comparing matched cohorts of patients with (pink curves) vs. without leukocytosis (blue curves). Shaded areas represent 95% confidence intervals.

**Fig AC** – Hazard ratio for the individual incidence (diagonal) and the co-occurrence (off-diagonal) of clinical features of long-COVID after a diagnosis of COVID-19 comparing matched cohorts of patients with vs. without leukocytosis (values higher than 1 indicate a significantly higher risk among those with leukocytosis). Higher values are shown by intensity of red and blue shading. Only HR reaching significance at p<0.05 are displayed and the corresponding p-values are presented on the right panel (^****^ p<0.0001, ^***^ p<0.001, ^**^ p<0.01, ^*^ p<0.05).

**Fig AD** – Clinical feature networks after a diagnosis of COVID-19 among matched cohorts of patients with vs. without leukocytosis. Dice’s coefficients graphically represented as the edges of the networks in the top panels are reported numerically in the bottom panels.

**Fig AE** – Hazard ratios comparing the incidence of clinical features of long-COVID between matched subgroups of patients diagnosed with COVID-19. The figure includes the spider plots shown in Figure 5 of the main manuscript. For each comparison of ‘Group A vs Group B’, a HR larger than 1 indicates that the incidence is higher in Group A (and vice versa for a HR lower than 1). Whether the HR is statistically significant is indicated with a star code underneath each feature: ^****^ p<0.0001, ^***^ p<0.001, ^**^ p<0.01, ^*^ p<0.05, n.s. p>0.05.


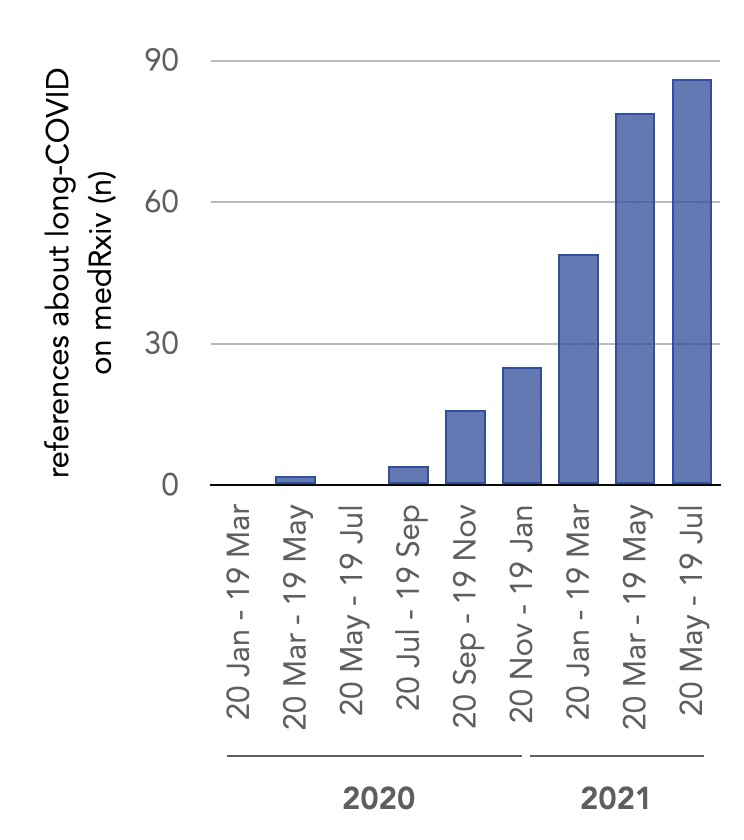


**Fig AF** – Number of references on medRxiv containing the term “long-COVID” in two-months interval since the beginning of the pandemic. This shows that the study (whose follow-up ended on December 16, 2020) largely took place at a time where public awareness long-COVID was significantly less than now. This suggests that public awareness alone is unlikely to have led to substantially more patients seeking medical attention for otherwise equal symptoms as the control cohort.
