# Supplementary material for: Incidence, co-occurrence, and evolution of long-COVID features: A 6-month retrospective cohort study of 273,618 survivors of COVID-19
Source: PLoS Med. 2021 Sep 28;18(9):e1003773. doi: 10.1371/journal.pmed.1003773 (PMC8478214; doi:10.1371/journal.pmed.1003773)
Supplement: S1 Tables — Table A. Characteristics of the unmatched COVID-19 cohort and the matched COVID-19 and influenza cohorts. Table B. Contributions of incidence (within 6 months of a diagnosis of COVID-19 vs. influenza) of subcategories making up the clinical features of long-COVID in matched cohorts. Table C. Incidence of long-COVID features in the whole cohort of patients with COVID-19 within the entire follow-up period (0–6 months), the first half of the follow-up period (0–3 months), and the second half of the follow-up period (3–6 months). In the analysis of the 3–6-month follow-up, those who had the long-COVID feature recorded in the first 3 months and then again in the next 3 months were included so that the sum of the incidences in the two-halves of the follow-up window exceeds the total incidence. Table D. Absolute risk increase in COVID-19 vs. influenza (a positive number indicates a higher risk in COVID-19) in the whole 0–6-month period as well as the “long” phase (3–6 months). Table E. 95% CIs corresponding to the entries in Fig 3A of the main manuscript, i.e., for the incidence (on the diagonal) and co-occurrence (off-diagonal) of long-COVID features in the 6 months after a diagnosis of COVID-19. Table F. 95% CIs corresponding to the entries in Fig 3B of the main manuscript, i.e., for the incidence (on the diagonal) and co-occurrence (off-diagonal) of long-COVID features in the period extending from 3 to 6 months after a diagnosis of COVID-19. Table G. 95% CIs corresponding to the entries in Fig 3C of the main manuscript, i.e., for the HRs of the incidence (on the diagonal) and co-occurrence (off-diagonal) of long-COVID features in the 6 months after a diagnosis of COVID-19 vs. influenza. All corresponding p-values were <0.0001 except for the co-occurrence of cognitive symptoms and myalgia (p = 0.0007). Table H. 95% CIs corresponding to the entries in Fig 3D of the main manuscript, i.e., for the HRs of the incidence (on the diagonal) and co-occurrence (off-diagonal) of lo [file pmed.1003773.s003.docx]

**Supplementary Tables**

**Table A** – Characteristics of the unmatched COVID-19 cohort and the matched COVID-19 and influenza cohorts. SMD = Standardised Mean Difference.

|  | **COVID-19 (unmatched)** | **COVID-19 (matched)** | **Influenza (matched)** | **SMD** |
| --- | --- | --- | --- | --- |
| Number | 273618 | 106578 | 106578 | - |
| DEMOGRAPHICS |  |  |  |  |
| Age; mean (SD); y | 46.3 (19.8) | 39.4 (18.4) | 38.3 (19.7) | 0.06 |
| Sex; n (%) |  |  |  |  |
| Female | 152157 (55.6) | 62293 (58.4) | 61419 (57.6) | 0.02 |
| Male | 120403 (44.0) | 44245 (41.5) | 45115 (42.3) | 0.02 |
| Other | 1058 (0.4) | 40 (0.04) | 44 (0.04) | 0.002 |
| Race; n (%) |  |  |  |  |
| White | 159028 (58.1) | 70243 (65.9) | 70128 (65.8) | 0.002 |
| Black or African American | 50329 (18.4) | 19349 (18.2) | 18583 (17.4) | 0.02 |
| Asian | 8227 (3.0) | 3740 (3.5) | 3509 (3.3) | 0.01 |
| American Indian or Alaska Native | 1075 (0.4) | 438 (0.4) | 435 (0.4) | 4.00E-04 |
| Native Hawaiian or Other Pacific Islander | 828 (0.3) | 243 (0.2) | 230 (0.2) | 0.003 |
| Unknown | 54131 (19.8) | 12565 (11.8) | 13693 (12.8) | 0.03 |
| Ethnicity; n (%) |  |  |  |  |
| Hispanic or Latino | 43254 (15.8) | 9014 (8.5) | 8944 (8.4) | 0.002 |
| Not Hispanic of Latino | 151246 (55.3) | 72644 (68.2) | 72075 (67.6) | 0.01 |
| Unknown | 79118 (28.9) | 24920 (23.4) | 25559 (24.0) | 0.01 |
| Problems related to housing and economic circumstances; n (%) | 2788 (1.0) | 911 (0.9) | 872 (0.8) | 0.004 |
| COMORBIDITIES; n (%) |  |  |  |  |
| Overweight and obesity | 50209 (18.4) | 19080 (17.9) | 18182 (17.1) | 0.02 |
| Hypertensive disease | 83970 (30.7) | 28188 (26.4) | 26189 (24.6) | 0.04 |
| Diabetes mellitus |  |  |  |  |
| Type 1 diabetes mellitus | 5764 (2.1) | 1993 (1.9) | 1893 (1.8) | 0.007 |
| Type 2 diabetes mellitus | 43127 (15.8) | 12087 (11.3) | 11254 (10.6) | 0.03 |
| Chronic lower respiratory diseases |  |  |  |  |
| Bronchitis; not specified as acute or chronic | 12716 (4.6) | 7928 (7.4) | 7758 (7.3) | 0.006 |
| Simple and mucopurulent chronic bronchitis | 1137 (0.4) | 596 (0.6) | 587 (0.6) | 0.001 |
| Unspecified chronic bronchitis | 1365 (0.5) | 641 (0.6) | 635 (0.6) | 7.00E-04 |
| Emphysema | 4127 (1.5) | 1745 (1.6) | 1714 (1.6) | 0.002 |
| Other chronic obstructive pulmonary disease | 12653 (4.6) | 5763 (5.4) | 5557 (5.2) | 0.009 |
| Asthma | 29556 (10.8) | 17097 (16.0) | 16418 (15.4) | 0.02 |
| Bronchiectasis | 1386 (0.5) | 715 (0.7) | 663 (0.6) | 0.006 |
| Nicotine dependence | 20091 (7.3) | 12602 (11.8) | 12111 (11.4) | 0.01 |
| Psychiatric comorbidities |  |  |  |  |
| Substance misuse | 29240 (10.7) | 16187 (15.2) | 15446 (14.5) | 0.02 |
| Psychotic disorders | 4583 (1.7) | 1417 (1.3) | 1320 (1.2) | 0.008 |
| Mood disorders | 42041 (15.4) | 19933 (18.7) | 18916 (17.7) | 0.02 |
| Anxiety disorders | 52299 (19.1) | 25731 (24.1) | 24302 (22.8) | 0.03 |
| Heart disease |  |  |  |  |
| Ischemic heart diseases | 24980 (9.1) | 7990 (7.5) | 7350 (6.9) | 0.02 |
| Other forms of heart disease | 49825 (18.2) | 16688 (15.7) | 15654 (14.7) | 0.03 |
| Chronic kidney diseases |  |  |  |  |
| Chronic kidney disease (CKD) | 18455 (6.7) | 5310 (5.0) | 5029 (4.7) | 0.01 |
| Hypertensive chronic kidney disease | 10353 (3.8) | 3092 (2.9) | 2884 (2.7) | 0.01 |
| Chronic liver disease |  |  |  |  |
| Alcoholic liver disease | 1286 (0.5) | 368 (0.3) | 339 (0.3) | 0.005 |
| Hepatic failure; not elsewhere classified | 1635 (0.6) | 454 (0.4) | 408 (0.4) | 0.007 |
| Chronic hepatitis; not elsewhere classified | 341 (0.1) | 118 (0.1) | 114 (0.1) | 0.001 |
| Fibrosis and cirrhosis of liver | 2934 (1.1) | 880 (0.8) | 828 (0.8) | 0.005 |
| Fatty (change of) liver; not elsewhere classified | 9632 (3.5) | 3363 (3.2) | 3113 (2.9) | 0.01 |
| Chronic passive congestion of liver | 1551 (0.6) | 678 (0.6) | 639 (0.6) | 0.005 |
| Portal hypertension | 1228 (0.4) | 342 (0.3) | 307 (0.3) | 0.006 |
| Other specified diseases of liver | 6228 (2.3) | 2263 (2.1) | 2174 (2.0) | 0.006 |
| Cerebral infarction | 6798 (2.5) | 1809 (1.7) | 1662 (1.6) | 0.01 |
| Dementia |  |  |  |  |
| Vascular dementia | 1481 (0.5) | 217 (0.2) | 197 (0.2) | 0.004 |
| Dementia in other diseases classified elsewhere | 2448 (0.9) | 332 (0.3) | 337 (0.3) | 8.00E-04 |
| Unspecified dementia | 5321 (1.9) | 781 (0.7) | 715 (0.7) | 0.007 |
| Alzheimer disease | 1951 (0.7) | 257 (0.2) | 256 (0.2) | 2.00E-04 |
| Frontotemporal dementia | 113 (0.04) | 19 (0.02) | 14 (0.01) | 0.004 |
| Dementia with Lewy bodies | 151 (0.06) | 30 (0.03) | 27 (0.03) | 0.002 |
| Neoplasms |  |  |  |  |
| Neoplasms (any) | 52535 (19.2) | 20945 (19.7) | 19474 (18.3) | 0.04 |
| Malignant neoplasms of lymphoid; hematopoietic and related tissue | 3104 (1.1) | 1454 (1.4) | 1346 (1.3) | 0.009 |
| Organ transplant |  |  |  |  |
| Renal Transplantation Procedures | 829 (0.3) | 212 (0.2) | 182 (0.2) | 0.007 |
| Liver Transplantation Procedures | 177 (0.07) | 38 (0.04) | 40 (0.04) | 0.001 |
| Psoriasis | 2965 (1.1) | 1372 (1.3) | 1314 (1.2) | 0.005 |
| Rheumatoid arthritis |  |  |  |  |
| Rheumatoid arthritis with rheumatoid factor | 1161 (0.4) | 484 (0.5) | 471 (0.4) | 0.002 |
| Other rheumatoid arthritis | 3937 (1.4) | 1596 (1.5) | 1557 (1.5) | 0.003 |
| Systemic lupus erythematosus (SLE) | 1635 (0.6) | 700 (0.7) | 678 (0.6) | 0.003 |
| Disorders involving the immune mechanism | 5959 (2.2) | 2630 (2.5) | 2481 (2.3) | 0.009 |

**Table B** – Contributions of incidence (within 6 months of a diagnosis of COVID-19 vs. influenza) of subcategories making up the clinical features of long-COVID in matched cohorts.

|  | **COVID-19** | **Influenza** |
| --- | --- | --- |
|  | **% (95% CI)** | **% (95% CI)** |
| **Mood disorders (F30-F39)** | 16.30 (15.89-16.72) | 11.34 (11.10-11.58) |
| **Anxiety disorders (F40-F48)** | 20.99 (20.52-21.46) | 14.48 (14.22-14.75) |
| **Myositis (M60)** | 0.72 (0.63-0.82) | 0.56 (0.50-0.61) |
| **Myalgia (M79.1)** | 3.29 (3.08-3.50) | 2.08 (1.97-2.19) |
| **Post-viral fatigue (G93.3)** | 2.02 (1.87-2.19) | 0.99 (0.92-1.06) |
| **Malaise/Fatigue (R53)** | 12.58 (12.19-12.97) | 6.44 (6.26-6.63) |
| **Migraine (G43)** | 5.78 (5.51-6.06) | 3.79 (3.65-3.94) |
| **Headache syndrome (G44)** | 2.86 (2.68-3.06) | 1.56 (1.47-1.66) |
| **Headache (R51)** | 3.98 (3.76-4.20) | 3.80 (3.66-3.95) |
| **Abdominal pain (R10)** | 13.89 (13.47-14.32) | 8.91 (8.69-9.12) |
| **Change in bowel habits (R19.4)** | 0.37 (0.30-0.46) | 0.22 (0.19-0.26) |
| **Diarrhoea (R19.7)** | 5.22 (4.98-5.48) | 3.43 (3.30-3.57) |
| **Dementia** | 1.10 (0.99-1.21) | 0.62 (0.56-0.68) |
| **Encephalopathy (G93.40)** | 0.96 (0.86-1.06) | 0.44 (0.39-0.49) |
| **Delirium (F05)** | 0.34 (0.28-0.42) | 0.14 (0.11-0.17) |
| **Mild cognitive impairment (MCI)** | 0.36 (0.29-0.44) | 0.17 (0.14-0.20) |
| **Somnolence/Stupor/Coma (R40)** | 0.88 (0.78-1.00) | 0.54 (0.49-0.60) |
| **Other cognitive symptoms (R41)** | 3.70 (3.50-3.92) | 1.96 (1.86-2.07) |
| **Symbolic dysfunction (R48)** | 0.13 (0.092-0.17) | 0.071 (0.054-0.094) |

**Table C** – Incidence of long-COVID features in the whole cohort of patients with COVID-19 within the entire follow-up period (0-6 months), the first half of the follow-up period (0-3 months), and the second half of the follow-up period (3-6 months). In the analysis of the 3-6 months follow-up, those who had the long-COVID feature recorded in the first 3 months and then again in the next 3 months were included so that the sum of the incidences in the two halves of the follow-up window exceeds the total incidence.

|  | 0-6 months | 0-3 months | 3-6 months |
| --- | --- | --- | --- |
|  | **% (95% CI)** | **% (95% CI)** | **% (95% CI)** |
| Chest/Throat pain | 12.60 (12.34-12.86) | 8.27 (8.11-8.43) | 5.71 (5.53-5.90) |
| Abnormal breathing | 18.71 (18.41-19.02) | 13.61 (13.42-13.81) | 7.94 (7.73-8.16) |
| Abdominal symptoms | 15.58 (15.26-15.87) | 9.45 (9.28-9.63) | 8.29 (8.07-8.51) |
| Fatigue | 12.82 (12.56-13.09) | 8.46 (8.30-8.63) | 5.87 (5.68-6.06) |
| Anxiety/Depression | 22.82 (22.48-23.14) | 15.87 (15.65-16.09) | 15.49 (15.21-15.77) |
| Pain | 11.60 (11.33-11.87) | 6.97 (6.82-7.13) | 7.19 (6.99-7.40) |
| Headache | 8.67 (8.44-8.90) | 5.76 (5.62-5.90) | 4.63 (4.47-4.80) |
| Cognitive symptoms | 7.88 (7.69-8.08) | 5.55 (5.43-5.69) | 3.95 (3.80-4.11) |
| Myalgia | 3.24 (3.09-3.38) | 1.91 (1.83-2.00) | 1.54 (1.44-1.64) |
| Any | 57.00 (56.59-57.43) | 42.43 (42.14-42.72) | 36.55 (36.18-36.94) |

**Table D** – Absolute risk increase in COVID-19 vs. influenza (a positive number indicates a higher risk in COVID-19) in the whole 0-6 months period as well as the ‘long’ phase (3-6 months).

|  | **0-6 months** | **3-6 months** |
| --- | --- | --- |
|  | **% (95% CI)** | **% (95% CI)** |
| **Anxiety/Depression** | 6.90 (6.26-7.54) | 4.97 (4.25-5.70) |
| **Chest/Throat Pain** | 5.63 (5.14-6.08) | 2.69 (2.26-3.15) |
| **Abnormal Breathing** | 8.71 (8.19-9.24) | 4.40 (3.90-4.88) |
| **Myalgia** | 1.43 (1.16-1.71) | 0.78 (0.53-1.04) |
| **Fatigue** | 5.79 (5.31-6.27) | 2.65 (2.22-3.08) |
| **Headache** | 2.55 (2.11-2.99) | 1.58 (1.13-2.04) |
| **Abdominal symptoms** | 5.92 (5.38-6.47) | 3.85 (3.27-4.41) |
| **Cognitive symptoms** | 2.40 (2.08-2.72) | 1.18 (0.89-1.48) |
| **Pain** | 3.76 (3.26-4.25) | 3.00 (2.49-3.52) |
| **Any** | 16.60 (15.84-17.33) | 12.64 (11.73-13.57) |

|  | **Chest/Throat pain** | **Abnormal breathing** | **Abdominal symptoms** | **Fatigue** | **Anxiety/Depression** | **Pain** | **Headache** | **Cognitive symptoms** | **Myalgia** |
| --- | --- | --- | --- | --- | --- | --- | --- | --- | --- |
| **Chest/Throat pain** | 12.34 - 12.86 | 5.85 - 6.22 | 3.64 - 3.96 | 2.95 - 3.26 | 4.21 - 4.54 | 2.38 - 2.67 | 1.93 - 2.17 | 1.31 - 1.51 | 0.84 - 1.01 |
| **Abnormal breathing** | 5.85 - 6.22 | 18.41 - 19.02 | 4.47 - 4.84 | 5.11 - 5.48 | 5.94 - 6.34 | 3.25 - 3.58 | 2.46 - 2.71 | 2.42 - 2.68 | 1.12 - 1.30 |
| **Abdominal symptoms** | 3.64 - 3.96 | 4.47 - 4.84 | 15.26 - 15.87 | 3.54 - 3.87 | 5.24 - 5.63 | 3.44 - 3.77 | 2.33 - 2.60 | 1.74 - 1.96 | 1.01 - 1.20 |
| **Fatigue** | 2.95 - 3.26 | 5.11 - 5.48 | 3.54 - 3.87 | 12.56 - 13.09 | 4.85 - 5.22 | 2.74 - 3.02 | 2.04 - 2.29 | 2.53 - 2.78 | 1.03 - 1.22 |
| **Anxiety/Depression** | 4.21 - 4.54 | 5.94 - 6.34 | 5.24 - 5.63 | 4.85 - 5.22 | 22.48 - 23.14 | 4.67 - 5.04 | 3.55 - 3.86 | 3.34 - 3.63 | 1.12 - 1.32 |
| **Pain** | 2.38 - 2.67 | 3.25 - 3.58 | 3.44 - 3.77 | 2.74 - 3.02 | 4.67 - 5.04 | 11.33 - 11.87 | 1.84 - 2.07 | 1.55 - 1.78 | 0.91 - 1.08 |
| **Headache** | 1.93 - 2.17 | 2.46 - 2.71 | 2.33 - 2.60 | 2.04 - 2.29 | 3.55 - 3.86 | 1.84 - 2.07 | 8.45 - 8.90 | 0.80 - 0.96 | 0.75 - 0.89 |
| **Cognitive symptoms** | 1.31 - 1.51 | 2.42 - 2.68 | 1.74 - 1.96 | 2.53 - 2.78 | 3.34 - 3.63 | 1.55 - 1.78 | 0.80 - 0.96 | 7.69 - 8.08 | 0.27 - 0.37 |
| **Myalgia** | 0.84 - 1.01 | 1.12 - 1.30 | 1.01 - 1.20 | 1.03 - 1.22 | 1.12 - 1.32 | 0.91 - 1.08 | 0.75 - 0.89 | 0.27 - 0.37 | 3.09 - 3.38 |

**Table E** – 95% confidence intervals corresponding to the entries in Figure 3A of the main manuscript, i.e. for the incidence (on the diagonal) and co-occurrence (off-diagonal) of long-COVID features in the 6-months after a diagnosis of COVID-19.

**Table F** – 95% confidence intervals corresponding to the entries in Figure 3B of the main manuscript, i.e. for the incidence (on the diagonal) and co-occurrence (off-diagonal) of long-COVID features in the period extending from 3 to 6 months after a diagnosis of COVID-19.

|  | **Chest/Throat pain** | **Abnormal breathing** | **Abdominal symptoms** | **Fatigue** | **Anxiety/Depression** | **Pain** | **Headache** | **Cognitive symptoms** | **Myalgia** |
| --- | --- | --- | --- | --- | --- | --- | --- | --- | --- |
| **Chest/Throat pain** | 5.53-5.90 | 1.93-2.16 | 1.16-1.35 | 0.83-0.99 | 1.67-1.87 | 0.86-1.01 | 0.57-0.71 | 0.37-0.47 | 0.22-0.30 |
| **Abnormal breathing** | 1.93-2.16 | 7.72-8.16 | 1.15-1.34 | 1.54-1.74 | 2.26-2.49 | 1.15-1.33 | 0.74-0.87 | 0.68-0.81 | 0.31-0.41 |
| **Abdominal symptoms** | 1.16-1.35 | 1.15-1.34 | 8.06-8.51 | 1.08-1.25 | 2.35-2.59 | 1.47-1.68 | 0.80-0.95 | 0.51-0.63 | 0.30-0.39 |
| **Fatigue** | 0.83-0.99 | 1.54-1.74 | 1.08-1.25 | 5.68-6.06 | 1.93-2.16 | 1.02-1.20 | 0.62-0.75 | 0.79-0.94 | 0.34-0.44 |
| **Anxiety/Depression** | 1.67-1.87 | 2.26-2.49 | 2.35-2.59 | 1.93-2.16 | 15.21-15.77 | 2.53-2.79 | 1.75-1.96 | 1.53-1.73 | 0.46-0.57 |
| **Pain** | 0.86-1.01 | 1.15-1.33 | 1.47-1.68 | 1.02-1.20 | 2.53-2.79 | 6.98-7.39 | 0.82-0.97 | 0.61-0.74 | 0.40-0.51 |
| **Headache** | 0.57-0.71 | 0.74-0.87 | 0.80-0.95 | 0.62-0.75 | 1.75-1.96 | 0.82-0.97 | 4.47-4.80 | 0.32-0.42 | 0.20-0.28 |
| **Cognitive symptoms** | 0.37-0.47 | 0.68-0.81 | 0.51-0.63 | 0.79-0.94 | 1.53-1.73 | 0.61-0.74 | 0.32-0.42 | 3.80-4.10 | 0.09-0.15 |
| **Myalgia** | 0.22-0.30 | 0.31-0.41 | 0.30-0.39 | 0.34-0.44 | 0.46-0.57 | 0.40-0.51 | 0.20-0.28 | 0.09-0.15 | 1.44-1.64 |

**Table G** – 95% confidence intervals corresponding to the entries in Figure 3C of the main manuscript, i.e. for the hazard ratios of the incidence (on the diagonal) and co-occurrence (off-diagonal) of long-COVID features in the 6-months after a diagnosis of COVID-19 vs. influenza. All corresponding p-values were < 0.0001 except for the co-occurrence of cognitive symptoms and myalgia (p=0.0007).

|  | **Chest/Throat pain** | **Abnormal breathing** | **Abdominal symptoms** | **Fatigue** | **Anxiety/Depression** | **Pain** | **Headache** | **Cognitive symptoms** | **Myalgia** |
| --- | --- | --- | --- | --- | --- | --- | --- | --- | --- |
| **Chest/Throat pain** | 1.83-1.99 | 2.33-2.64 | 1.93-2.29 | 2.31-2.86 | 1.85-2.13 | 1.73-2.10 | 1.63-2.01 | 1.79-2.48 | 1.59-2.21 |
| **Abnormal breathing** | 2.33-2.64 | 1.98-2.11 | 2.12-2.45 | 2.48-2.87 | 1.98-2.23 | 1.78-2.11 | 1.94-2.31 | 2.09-2.61 | 1.94-2.59 |
| **Abdominal symptoms** | 1.93-2.29 | 2.12-2.45 | 1.54-1.64 | 2.00-2.38 | 1.68-1.90 | 1.72-2.03 | 1.48-1.76 | 2.00-2.63 | 1.70-2.29 |
| **Fatigue** | 2.31-2.86 | 2.48-2.87 | 2.00-2.38 | 1.86-2.02 | 1.92-2.19 | 1.71-2.08 | 1.76-2.16 | 2.01-2.56 | 1.89-2.53 |
| **Anxiety/Depression** | 1.85-2.13 | 1.98-2.23 | 1.68-1.90 | 1.92-2.19 | 1.44-1.52 | 1.49-1.70 | 1.48-1.70 | 1.80-2.16 | 1.45-1.92 |
| **Pain** | 1.73-2.10 | 1.78-2.11 | 1.72-2.03 | 1.71-2.08 | 1.49-1.70 | 1.39-1.52 | 1.45-1.76 | 1.56-2.08 | 1.35-1.81 |
| **Headache** | 1.63-2.01 | 1.94-2.31 | 1.48-1.76 | 1.76-2.16 | 1.48-1.70 | 1.45-1.76 | 1.38-1.50 | 1.49-2.10 | 1.41-1.88 |
| **Cognitive symptoms** | 1.79-2.48 | 2.09-2.61 | 2.00-2.63 | 2.01-2.56 | 1.80-2.16 | 1.56-2.08 | 1.49-2.10 | 1.71-1.92 | 1.72-3.74 |
| **Myalgia** | 1.59-2.21 | 1.94-2.59 | 1.70-2.29 | 1.89-2.53 | 1.45-1.92 | 1.35-1.81 | 1.41-1.88 | 1.72-3.74 | 1.55-1.81 |

**Table H** – 95% confidence intervals corresponding to the entries in Figure 3D of the main manuscript, i.e. for the hazard ratios of the incidence (on the diagonal) and co-occurrence (off-diagonal) of long-COVID features in the period extending from 3 to 6 months after a diagnosis of COVID-19 vs. influenza. All corresponding p-values were < 0.01 except for the co-occurrence of myalgia and headache (p=0.1476), myalgia and cognitive symptoms (p=0.1292), and myalgia and pain (p=0.0139).

|  | **Chest/Throat pain** | **Abnormal breathing** | **Abdominal symptoms** | **Fatigue** | **Anxiety/Depression** | **Pain** | **Headache** | **Cognitive symptoms** | **Myalgia** |
| --- | --- | --- | --- | --- | --- | --- | --- | --- | --- |
| **Chest/Throat pain** | 1.60-1.86 | 1.83-2.38 | 1.53-2.14 | 1.63-2.50 | 1.55-2.00 | 1.47-2.13 | 1.22-1.85 | 1.32-2.57 | 1.18-2.32 |
| **Abnormal breathing** | 1.83-2.38 | 1.84-2.09 | 1.62-2.26 | 1.80-2.45 | 1.88-2.36 | 1.80-2.52 | 1.45-2.11 | 1.47-2.37 | 1.58-2.94 |
| **Abdominal symptoms** | 1.53-2.14 | 1.62-2.26 | 1.48-1.67 | 1.71-2.43 | 1.62-2.02 | 1.68-2.22 | 1.23-1.72 | 1.41-2.51 | 1.29-2.35 |
| **Fatigue** | 1.63-2.50 | 1.80-2.45 | 1.71-2.43 | 1.69-1.95 | 1.51-1.94 | 1.56-2.20 | 1.22-1.83 | 1.70-2.63 | 1.31-2.52 |
| **Anxiety/Depression** | 1.55-2.00 | 1.88-2.36 | 1.62-2.02 | 1.51-1.94 | 1.34-1.46 | 1.52-1.89 | 1.33-1.65 | 1.35-1.89 | 1.29-2.07 |
| **Pain** | 1.47-2.13 | 1.80-2.52 | 1.68-2.22 | 1.56-2.20 | 1.52-1.89 | 1.43-1.63 | 1.51-2.10 | 1.22-2.11 | 1.09-1.83 |
| **Headache** | 1.22-1.85 | 1.45-2.11 | 1.23-1.72 | 1.22-1.83 | 1.33-1.65 | 1.51-2.10 | 1.26-1.46 | 1.18-2.21 | 0.90-1.69 |
| **Cognitive symptoms** | 1.32-2.57 | 1.47-2.37 | 1.41-2.51 | 1.70-2.63 | 1.35-1.89 | 1.22-2.11 | 1.18-2.21 | 1.49-1.84 | 0.63-3.94 |
| **Myalgia** | 1.18-2.32 | 1.58-2.94 | 1.29-2.35 | 1.31-2.52 | 1.29-2.07 | 1.09-1.83 | 0.90-1.69 | 0.63-3.94 | 1.47-1.91 |

**Table I** – P-values for the test of proportional hazards (obtained using the generalized Schoenfeld test) for the main analysis (1 day to 6 months follow-up) and the analysis restricted to the 3 months-6 months follow-up. A value lower than 0.05 indicates evidence for non-proportional hazards.

|  | **1 day-6 months** | **3 months - 6 months** |
| --- | --- | --- |
| **Anxiety/Depression** | <0.0001 | 0.0062 |
| **Chest/Throat pain** | 0.00061 | 1 |
| **Abnormal breathing** | 0.0046 | 0.94 |
| **Myalgia** | 0.78 | 0.035 |
| **Fatigue** | 0.057 | <0.0001 |
| **Headache** | <0.0001 | 0.00083 |
| **Abdominal symptoms** | 0.22 | 0.26 |
| **Cognitive symptoms** | 0.36 | 0.68 |
| **Pain** | 0.0042 | 0.12 |
| **Any** | 0.13 | 0.95 |
| **Anxiety/Depression & Chest/Throat pain** | 0.19 | 0.12 |
| **Anxiety/Depression & Abnormal breathing** | <0.0001 | 0.5 |
| **Anxiety/Depression & Myalgia** | 0.28 | 0.97 |
| **Anxiety/Depression & Fatigue** | 0.19 | 0.054 |
| **Anxiety/Depression & Headache** | <0.0001 | 0.0055 |
| **Anxiety/Depression & Abdominal symptoms** | 0.16 | 0.75 |
| **Anxiety/Depression & Cognitive symptoms** | 0.74 | 0.99 |
| **Anxiety/Depression & Pain** | 0.91 | 0.92 |
| **Chest/Throat pain & Abnormal breathing** | 0.00014 | 0.52 |
| **Chest/Throat pain & Myalgia** | 0.63 | 0.099 |
| **Chest/Throat pain & Fatigue** | 0.8 | 0.92 |
| **Chest/Throat pain & Headache** | 0.0052 | 0.2 |
| **Chest/Throat pain & Abdominal symptoms** | 0.18 | 0.037 |
| **Chest/Throat pain & Cognitive symptoms** | 0.1 | 0.38 |
| **Chest/Throat pain & Pain** | 0.74 | 0.53 |
| **Abnormal breathing & Myalgia** | 0.044 | 0.018 |
| **Abnormal breathing & Fatigue** | 0.11 | 0.13 |
| **Abnormal breathing & Headache** | 0.009 | 0.63 |
| **Abnormal breathing & Abdominal symptoms** | 0.0012 | 0.71 |
| **Abnormal breathing & Cognitive symptoms** | 0.36 | 0.28 |
| **Abnormal breathing & Pain** | 0.73 | 0.49 |
| **Myalgia & Fatigue** | 0.98 | 0.38 |
| **Myalgia & Headache** | 0.37 | 0.0095 |
| **Myalgia & Abdominal symptoms** | 0.91 | 0.051 |
| **Myalgia & Cognitive symptoms** | 0.081 | 0.93 |
| **Myalgia & Pain** | 0.85 | 0.91 |
| **Fatigue & Headache** | 0.42 | 0.37 |
| **Fatigue & Abdominal symptoms** | 0.27 | 0.44 |
| **Fatigue & Cognitive symptoms** | 0.45 | 0.0011 |
| **Fatigue & Pain** | 0.25 | 0.9 |
| **Headache & Abdominal symptoms** | 0.067 | 0.029 |
| **Headache & Cognitive symptoms** | 0.63 | 0.75 |
| **Headache & Pain** | 0.83 | 0.078 |
| **Abdominal symptoms & Cognitive symptoms** | 0.59 | 0.42 |
| **Abdominal symptoms & Pain** | 0.24 | 0.016 |
| **Cognitive symptoms & Pain** | 0.19 | 0.36 |

**Table J** – Average degrees of the clinical feature networks in the different comparisons between cohorts. P-values were obtained using permutation tests.

|  | **Degree in Cohort 1** | **Degree in Cohort 2** | **p-value** |
| --- | --- | --- | --- |
| **COVID vs. Influenza (1 day-6 months)** | 1.70 | 1.39 | < 0.001 |
| **COVID vs. Influenza (3 months-6 months)** | 1.21 | 1.06 | 0.21 |
| **Female vs. Male** | 1.64 | 1.50 | 0.14 |
| **Age 45+ vs. Age 10-44** | 1.56 | 1.60 | 0.72 |
| **Age 22-44 vs. Age 10-21** | 1.31 | 1.26 | 0.83 |
| **Age 65+ vs. Age 45-64** | 1.57 | 1.73 | 0.29 |
| **Non-White vs. White** | 1.65 | 1.62 | 0.79 |
| **Hospitalization vs. No Hospitalization** | 1.81 | 1.61 | 0.059 |
| **Leukocytosis vs. No leukocytosis** | 1.84 | 1.78 | 0.67 |
| **ITU admission vs. No ITU admission** | 2.05 | 1.83 | 0.19 |

|  | **Female** | **Male** | **Non-White** | **White** | **Age 10-21** | **Age 22-44** | **Age 45-64** | **Age 65+** |
| --- | --- | --- | --- | --- | --- | --- | --- | --- |
|  | **% (95% CI)** | **% (95% CI)** | **% (95% CI)** | **% (95% CI)** | **% (95% CI)** | **% (95% CI)** | **% (95% CI)** | **% (95% CI)** |
| **Chest/Throat Pain** | 12.68 (12.35-13.00) | 12.49 (12.07-12.90) | 15.26 (14.70-15.80) | 11.55 (11.22-11.88) | 8.40 (7.43-9.35) | 12.31 (11.87-12.77) | 14.52 (14.04-15.01) | 11.62 (11.13-12.13) |
| **Abnormal Breathing** | 18.24 (17.87-18.60) | 19.44 (18.96-19.93) | 21.06 (20.49-21.64) | 18.01 (17.62-18.40) | 9.90 (8.88-10.87) | 15.48 (15.01-15.97) | 21.91 (21.37-22.44) | 21.75 (21.11-22.38) |
| **Abdominal symptoms** | 17.58 (17.19-17.99) | 12.51 (12.10-12.95) | 16.68 (16.10-17.26) | 15.07 (14.69-15.46) | 14.20 (12.96-15.49) | 17.67 (17.03-18.31) | 15.51 (14.99-16.02) | 13.58 (13.03-14.14) |
| **Fatigue** | 13.38 (13.03-13.71) | 11.97 (11.56-12.37) | 12.12 (11.65-12.62) | 13.36 (13.01-13.71) | 7.51 (6.58-8.48) | 10.43 ( 9.98-10.88) | 13.49 (13.05-13.95) | 17.14 (16.57-17.71) |
| **Anxiety/Depression** | 26.65 (26.21-27.11) | 17.01 (16.53-17.48) | 19.46 (18.89-20.01) | 25.62 (25.16-26.10) | 18.58 (17.33-19.87) | 24.30 (23.71-24.89) | 22.78 (22.23-23.34) | 22.49 (21.81-23.14) |
| **Pain** | 12.15 (11.80-12.49) | 10.78 (10.38-11.19) | 12.32 (11.79-12.82) | 12.00 (11.63-12.36) | 6.22 (5.43-7.01) | 8.64 (8.23-9.04) | 14.10 (13.61-14.59) | 13.71 (13.16-14.25) |
| **Headache** | 11.20 (10.89-11.51) | 4.86 (4.60-5.13) | 8.36 (7.95-8.77) | 8.88 (8.57-9.18) | 9.15 (8.21-10.09) | 11.63 (11.16-12.10) | 8.86 (8.49-9.22) | 4.28 (3.97-4.60) |
| **Cognitive symptoms** | 7.29 (7.03-7.55) | 8.76 (8.41-9.09) | 8.04 (7.67-8.42) | 8.14 (7.86-8.41) | 2.31 (1.80-2.79) | 2.98 (2.74-3.22) | 5.85 (5.55-6.15) | 19.53 (18.98-20.10) |
| **Myalgia** | 3.60 (3.40-3.81) | 2.68 (2.46-2.90) | 3.02 (2.76-3.28) | 3.27 (3.07-3.47) | 2.25 (1.74-2.76) | 3.68 (3.41-3.95) | 3.82 (3.54-4.07) | 2.10 (1.87-2.33) |
| **Any** | 59.77 (59.26-60.28) | 52.81 (52.15-53.50) | 57.57 (56.80-58.35) | 57.99 (57.43-58.54) | 46.42 (44.77-48.06) | 55.06 (54.34-55.77) | 58.92 (58.24-59.59) | 61.05 (60.29-61.81) |

**Table K** – 6-month incidence of individual long-COVID features and of any feature in different subgroups of patients (defined by sex, race, or age) diagnosed with COVID-19

**Table L** – 6-month incidence of individual long-COVID features and of any feature in different subgroups of patients defined by indices of severity of COVID-19 illness.

|  | **Non-hospitalised patients** | **Hospitalised patients** | **Patients with leukocytosis** | **Patients with ITU admission** |
| --- | --- | --- | --- | --- |
|  | **% (95% CI)** | **% (95% CI)** | **% (95% CI)** | **% (95% CI)** |
| **Chest/Throat Pain** | 11.35 (11.06-11.63) | 15.50 (14.92-16.07) | 14.47 (13.81-15.15) | 16.52 (15.44-17.61) |
| **Abnormal Breathing** | 15.36 (15.04-15.68) | 27.37 (26.70-28.05) | 28.13 (27.30-28.93) | 39.00 (37.56-40.43) |
| **Abdominal symptoms** | 14.72 (14.35-15.09) | 17.10 (16.47-17.71) | 16.70 (15.97-17.46) | 18.24 (17.09-19.42) |
| **Fatigue** | 11.05 (10.73-11.34) | 17.65 (17.05-18.26) | 18.77 (18.05-19.48) | 26.23 (24.90-27.54) |
| **Anxiety/Depression** | 22.53 (22.13-22.92) | 23.01 (22.37-23.65) | 24.88 (24.08-25.69) | 27.04 (25.66-28.34) |
| **Pain** | 10.85 (10.54-11.16) | 13.61 (13.06-14.16) | 14.31 (13.64-14.96) | 15.44 (14.39-16.54) |
| **Headache** | 9.43 (9.15-9.70) | 5.88 (5.52-6.25) | 5.14 (4.70-5.58) | 4.45 (3.81-5.11) |
| **Cognitive symptoms** | 4.91 (4.71-5.11) | 16.66 (16.14-17.17) | 17.18 (16.51-17.86) | 27.75 (26.58-28.91) |
| **Myalgia** | 3.42 (3.24-3.59) | 2.41 (2.17-2.66) | 2.33 (2.03-2.62) | 2.46 (1.95-2.98) |
| **Any** | 54.49 (54.03-54.96) | 63.64 (62.86-64.44) | 64.83 (63.86-65.72) | 73.22 (71.91-74.53) |

**Table M** – Characteristics of the female and male COVID-19 cohorts after propensity score matching**.** SMD = Standardised Mean Difference.

|  | **Female** | **Male** | **SMD** |
| --- | --- | --- | --- |
| Number | 106775 | 106775 | - |
| DEMOGRAPHICS |  |  |  |
| Age; mean (SD); y | 45.8 (20.2) | 46.0 (19.7) | 0.01 |
| Sex; n (%) |  |  |  |
| Female | 106775 (100.0) | 0 (0.0) | - |
| Male | 0 (0.0) | 106775 (100.0) | - |
| Other | 0 (0.0) | 0 (0.0) | - |
| Race; n (%) |  |  |  |
| White | 62071 (58.1) | 62361 (58.4) | 0.006 |
| Black or African American | 18171 (17.0) | 17919 (16.8) | 0.006 |
| Asian | 3391 (3.2) | 3359 (3.1) | 0.002 |
| American Indian or Alaska Native | 410 (0.4) | 420 (0.4) | 0.002 |
| Native Hawaiian or Other Pacific Islander | 358 (0.3) | 346 (0.3) | 0.002 |
| Unknown | 22374 (21.0) | 22370 (21.0) | 9.00E-05 |
| Ethnicity; n (%) |  |  |  |
| Hispanic or Latino | 16819 (15.8) | 17078 (16.0) | 0.007 |
| Not Hispanic of Latino | 57290 (53.7) | 57326 (53.7) | 7.00E-04 |
| Unknown | 32666 (30.6) | 32371 (30.3) | 0.006 |
| Problems related to housing and economic circumstances; n (%) | 934 (0.9) | 947 (0.9) | 0.001 |
| COMORBIDITIES; n (%) |  |  |  |
| Overweight and obesity | 15820 (14.8) | 16301 (15.3) | 0.01 |
| Hypertensive disease | 30254 (28.3) | 30648 (28.7) | 0.008 |
| Diabetes mellitus |  |  |  |
| Type 1 diabetes mellitus | 2019 (1.9) | 2064 (1.9) | 0.003 |
| Type 2 diabetes mellitus | 15502 (14.5) | 15813 (14.8) | 0.008 |
| Chronic lower respiratory diseases |  |  |  |
| Bronchitis; not specified as acute or chronic | 3889 (3.6) | 4008 (3.8) | 0.006 |
| Simple and mucopurulent chronic bronchitis | 366 (0.3) | 387 (0.4) | 0.003 |
| Unspecified chronic bronchitis | 447 (0.4) | 448 (0.4) | 1.00E-04 |
| Emphysema | 1427 (1.3) | 1448 (1.4) | 0.002 |
| Other chronic obstructive pulmonary disease | 4347 (4.1) | 4409 (4.1) | 0.003 |
| Asthma | 8979 (8.4) | 8944 (8.4) | 0.001 |
| Bronchiectasis | 450 (0.4) | 473 (0.4) | 0.003 |
| Nicotine dependence | 7075 (6.6) | 7160 (6.7) | 0.003 |
| Psychiatric comorbidities |  |  |  |
| Substance misuse | 10202 ( 9.6) | 10343 ( 9.7) | 0.004 |
| Psychotic disorders | 1609 (1.5) | 1630 (1.5) | 0.002 |
| Mood disorders | 11723 (11.0) | 11887 (11.1) | 0.005 |
| Anxiety disorders | 15080 (14.1) | 15249 (14.3) | 0.005 |
| Heart disease |  |  |  |
| Ischemic heart diseases | 8725 (8.2) | 8853 (8.3) | 0.004 |
| Other forms of heart disease | 17489 (16.4) | 17897 (16.8) | 0.01 |
| Chronic kidney diseases |  |  |  |
| Chronic kidney disease (CKD) | 6529 (6.1) | 6652 (6.2) | 0.005 |
| Hypertensive chronic kidney disease | 3622 (3.4) | 3686 (3.5) | 0.003 |
| Chronic liver disease |  |  |  |
| Alcoholic liver disease | 367 (0.3) | 393 (0.4) | 0.004 |
| Hepatic failure; not elsewhere classified | 541 (0.5) | 560 (0.5) | 0.002 |
| Chronic hepatitis; not elsewhere classified | 100 (0.09) | 101 (0.1) | 3.00E-04 |
| Fibrosis and cirrhosis of liver | 986 (0.9) | 1001 (0.9) | 0.001 |
| Fatty (change of) liver; not elsewhere classified | 3261 (3.1) | 3290 (3.1) | 0.002 |
| Chronic passive congestion of liver | 489 (0.5) | 493 (0.5) | 6.00E-04 |
| Portal hypertension | 394 (0.4) | 403 (0.4) | 0.001 |
| Other specified diseases of liver | 2001 (1.9) | 2029 (1.9) | 0.002 |
| Cerebral infarction | 2377 (2.2) | 2428 (2.3) | 0.003 |
| Dementia |  |  |  |
| Vascular dementia | 500 (0.5) | 509 (0.5) | 0.001 |
| Dementia in other diseases classified elsewhere | 826 (0.8) | 824 (0.8) | 2.00E-04 |
| Unspecified dementia | 1858 (1.7) | 1852 (1.7) | 4.00E-04 |
| Alzheimer disease | 638 (0.6) | 623 (0.6) | 0.002 |
| Frontotemporal dementia | 35 (0.03) | 43 (0.04) | 0.004 |
| Dementia with Lewy bodies | 51 (0.05) | 48 (0.04) | 0.001 |
| Neoplasms |  |  |  |
| Neoplasms (any) | 16878 (15.8) | 17462 (16.4) | 0.01 |
| Malignant neoplasms of lymphoid; hematopoietic and related tissue | 1133 (1.1) | 1125 (1.1) | 7.00E-04 |
| Organ transplant |  |  |  |
| Renal Transplantation Procedures | 280 (0.3) | 291 (0.3) | 0.002 |
| Liver Transplantation Procedures | 56 (0.05) | 60 (0.06) | 0.002 |
| Psoriasis | 1001 (0.9) | 1029 (1.0) | 0.003 |
| Rheumatoid arthritis |  |  |  |
| Rheumatoid arthritis with rheumatoid factor | 244 (0.2) | 247 (0.2) | 6.00E-04 |
| Other rheumatoid arthritis | 903 (0.8) | 901 (0.8) | 2.00E-04 |
| Systemic lupus erythematosus (SLE) | 188 (0.2) | 179 (0.2) | 0.002 |
| Disorders involving the immune mechanism | 1884 (1.8) | 1944 (1.8) | 0.004 |

**Table N** – Characteristics of the non-white & white COVID-19 cohorts after propensity score matching**.** SMD = Standardised Mean Difference.

|  | **Non-White** | **White** | **SMD** |
| --- | --- | --- | --- |
| Number | 58721 | 58721 | - |
| DEMOGRAPHICS |  |  |  |
| Age; mean (SD); y | 46.5 (18.5) | 47.5 (19.5) | 0.05 |
| Sex; n (%) |  |  |  |
| Female | 34938 (59.5) | 33820 (57.6) | 0.04 |
| Male | 23770 (40.5) | 24889 (42.4) | 0.04 |
| Other | 13 (0.02) | 12 (0.02) | 0.001 |
| Race; n (%) |  |  |  |
| White | 0 (0.0) | 58721 (100.0) | - |
| Black or African American | 48678 (82.9) | 0 (0.0) | - |
| Asian | 8161 (13.9) | 0 (0.0) | - |
| American Indian or Alaska Native | 1059 (1.8) | 0 (0.0) | - |
| Native Hawaiian or Other Pacific Islander | 823 (1.4) | 0 (0.0) | - |
| Unknown | 0 (0.0) | 0 (0.0) | - |
| Ethnicity; n (%) |  |  |  |
| Hispanic or Latino | 1557 (2.7) | 9438 (16.1) | 0.5 |
| Not Hispanic of Latino | 40092 (68.3) | 37091 (63.2) | 0.1 |
| Unknown | 17072 (29.1) | 12192 (20.8) | 0.2 |
| Problems related to housing and economic circumstances; n (%) | 818 (1.4) | 787 (1.3) | 0.005 |
| COMORBIDITIES; n (%) |  |  |  |
| Overweight and obesity | 14045 (23.9) | 13998 (23.8) | 0.002 |
| Hypertensive disease | 23337 (39.7) | 23446 (39.9) | 0.004 |
| Diabetes mellitus |  |  |  |
| Type 1 diabetes mellitus | 1660 (2.8) | 1647 (2.8) | 0.001 |
| Type 2 diabetes mellitus | 12546 (21.4) | 12778 (21.8) | 0.01 |
| Chronic lower respiratory diseases |  |  |  |
| Bronchitis; not specified as acute or chronic | 2910 (5.0) | 2676 (4.6) | 0.02 |
| Simple and mucopurulent chronic bronchitis | 290 (0.5) | 275 (0.5) | 0.004 |
| Unspecified chronic bronchitis | 346 (0.6) | 329 (0.6) | 0.004 |
| Emphysema | 1005 (1.7) | 952 (1.6) | 0.007 |
| Other chronic obstructive pulmonary disease | 2929 (5.0) | 2810 (4.8) | 0.009 |
| Asthma | 7975 (13.6) | 7884 (13.4) | 0.005 |
| Bronchiectasis | 368 (0.6) | 367 (0.6) | 2.00E-04 |
| Nicotine dependence | 5456 (9.3) | 5407 (9.2) | 0.003 |
| Psychiatric comorbidities |  |  |  |
| Substance misuse | 7646 (13.0) | 7539 (12.8) | 0.005 |
| Psychotic disorders | 1292 (2.2) | 1298 (2.2) | 7.00E-04 |
| Mood disorders | 8642 (14.7) | 8544 (14.6) | 0.005 |
| Anxiety disorders | 10270 (17.5) | 10092 (17.2) | 0.008 |
| Heart disease |  |  |  |
| Ischemic heart diseases | 5927 (10.1) | 5716 ( 9.7) | 0.01 |
| Other forms of heart disease | 12319 (21.0) | 12228 (20.8) | 0.004 |
| Chronic kidney diseases |  |  |  |
| Chronic kidney disease (CKD) | 5561 (9.5) | 5567 (9.5) | 3.00E-04 |
| Hypertensive chronic kidney disease | 3285 (5.6) | 3298 (5.6) | 0.001 |
| Chronic liver disease |  |  |  |
| Alcoholic liver disease | 214 (0.4) | 191 (0.3) | 0.007 |
| Hepatic failure; not elsewhere classified | 314 (0.5) | 284 (0.5) | 0.007 |
| Chronic hepatitis; not elsewhere classified | 95 (0.2) | 82 (0.1) | 0.006 |
| Fibrosis and cirrhosis of liver | 667 (1.1) | 671 (1.1) | 6.00E-04 |
| Fatty (change of) liver; not elsewhere classified | 1794 (3.1) | 1667 (2.8) | 0.01 |
| Chronic passive congestion of liver | 435 (0.7) | 445 (0.8) | 0.002 |
| Portal hypertension | 191 (0.3) | 179 (0.3) | 0.004 |
| Other specified diseases of liver | 1334 (2.3) | 1254 (2.1) | 0.009 |
| Cerebral infarction | 1928 (3.3) | 1916 (3.3) | 0.001 |
| Dementia |  |  |  |
| Vascular dementia | 376 (0.6) | 369 (0.6) | 0.002 |
| Dementia in other diseases classified elsewhere | 490 (0.8) | 462 (0.8) | 0.005 |
| Unspecified dementia | 1141 (1.9) | 1097 (1.9) | 0.005 |
| Alzheimer disease | 396 (0.7) | 367 (0.6) | 0.006 |
| Frontotemporal dementia | 17 (0.03) | 23 (0.04) | 0.006 |
| Dementia with Lewy bodies | 16 (0.03) | 26 (0.04) | 0.009 |
| Neoplasms |  |  |  |
| Neoplasms (any) | 10997 (18.7) | 10416 (17.7) | 0.03 |
| Malignant neoplasms of lymphoid; hematopoietic and related tissue | 634 (1.1) | 599 (1.0) | 0.006 |
| Organ transplant |  |  |  |
| Renal Transplantation Procedures | 275 (0.5) | 270 (0.5) | 0.001 |
| Liver Transplantation Procedures | 27 (0.05) | 33 (0.06) | 0.005 |
| Psoriasis | 379 (0.6) | 375 (0.6) | 9.00E-04 |
| Rheumatoid arthritis |  |  |  |
| Rheumatoid arthritis with rheumatoid factor | 269 (0.5) | 263 (0.4) | 0.002 |
| Other rheumatoid arthritis | 894 (1.5) | 819 (1.4) | 0.01 |
| Systemic lupus erythematosus (SLE) | 489 (0.8) | 485 (0.8) | 8.00E-04 |
| Disorders involving the immune mechanism | 1587 (2.7) | 1554 (2.6) | 0.003 |

**Table O** – Characteristics of the age 45+ & age 10-44 COVID-19 cohorts after propensity score matching**.** SMD = Standardised Mean Difference.

|  | **Age 45+** | **Age 10-44** | **SMD** |
| --- | --- | --- | --- |
| Number | 70671 | 70671 | - |
| DEMOGRAPHICS |  |  |  |
| Age; mean (SD); y | 58.1 (10.5) | 29.8 (8.9) | 2.9 |
| Sex; n (%) |  |  |  |
| Female | 38979 (55.2) | 39083 (55.3) | 0.003 |
| Male | 31384 (44.4) | 31269 (44.2) | 0.003 |
| Other | 308 (0.4) | 319 (0.5) | 0.002 |
| Race; n (%) |  |  |  |
| White | 41173 (58.3) | 41169 (58.3) | 1.00E-04 |
| Black or African American | 12028 (17.0) | 11822 (16.7) | 0.008 |
| Asian | 2198 (3.1) | 2202 (3.1) | 3.00E-04 |
| American Indian or Alaska Native | 292 (0.4) | 284 (0.4) | 0.002 |
| Native Hawaiian or Other Pacific Islander | 199 (0.3) | 213 (0.3) | 0.004 |
| Unknown | 14781 (20.9) | 14981 (21.2) | 0.007 |
| Ethnicity; n (%) |  |  |  |
| Hispanic or Latino | 11143 (15.8) | 11370 (16.1) | 0.009 |
| Not Hispanic of Latino | 37597 (53.2) | 37403 (52.9) | 0.006 |
| Unknown | 21931 (31.0) | 21898 (31.0) | 0.001 |
| Problems related to housing and economic circumstances; n (%) | 489 (0.7) | 539 (0.8) | 0.008 |
| COMORBIDITIES; n (%) |  |  |  |
| Overweight and obesity | 10549 (14.9) | 10397 (14.7) | 0.006 |
| Hypertensive disease | 11412 (16.1) | 11751 (16.6) | 0.01 |
| Diabetes mellitus |  |  |  |
| Type 1 diabetes mellitus | 888 (1.3) | 901 (1.3) | 0.002 |
| Type 2 diabetes mellitus | 5522 (7.8) | 5501 (7.8) | 0.001 |
| Chronic lower respiratory diseases |  |  |  |
| Bronchitis; not specified as acute or chronic | 2456 (3.5) | 2497 (3.5) | 0.003 |
| Simple and mucopurulent chronic bronchitis | 92 (0.1) | 91 (0.1) | 4.00E-04 |
| Unspecified chronic bronchitis | 107 (0.2) | 115 (0.2) | 0.003 |
| Emphysema | 137 (0.2) | 128 (0.2) | 0.003 |
| Other chronic obstructive pulmonary disease | 564 (0.8) | 573 (0.8) | 0.001 |
| Asthma | 6033 (8.5) | 6301 (8.9) | 0.01 |
| Bronchiectasis | 157 (0.2) | 144 (0.2) | 0.004 |
| Nicotine dependence | 3842 (5.4) | 4096 (5.8) | 0.02 |
| Psychiatric comorbidities |  |  |  |
| Substance misuse | 5693 (8.1) | 5999 (8.5) | 0.02 |
| Psychotic disorders | 677 (1.0) | 744 (1.1) | 0.01 |
| Mood disorders | 8175 (11.6) | 8439 (11.9) | 0.01 |
| Anxiety disorders | 10938 (15.5) | 11340 (16.0) | 0.02 |
| Heart disease |  |  |  |
| Ischemic heart diseases | 1453 (2.1) | 1354 (1.9) | 0.01 |
| Other forms of heart disease | 6973 ( 9.9) | 7197 (10.2) | 0.01 |
| Chronic kidney diseases |  |  |  |
| Chronic kidney disease (CKD) | 1660 (2.3) | 1641 (2.3) | 0.002 |
| Hypertensive chronic kidney disease | 965 (1.4) | 961 (1.4) | 5.00E-04 |
| Chronic liver disease |  |  |  |
| Alcoholic liver disease | 190 (0.3) | 193 (0.3) | 8.00E-04 |
| Hepatic failure; not elsewhere classified | 242 (0.3) | 235 (0.3) | 0.002 |
| Chronic hepatitis; not elsewhere classified | 45 (0.06) | 38 (0.05) | 0.004 |
| Fibrosis and cirrhosis of liver | 328 (0.5) | 319 (0.5) | 0.002 |
| Fatty (change of) liver; not elsewhere classified | 1875 (2.7) | 1881 (2.7) | 5.00E-04 |
| Chronic passive congestion of liver | 263 (0.4) | 242 (0.3) | 0.005 |
| Portal hypertension | 181 (0.3) | 157 (0.2) | 0.007 |
| Other specified diseases of liver | 978 (1.4) | 978 (1.4) | 0 |
| Cerebral infarction | 490 (0.7) | 481 (0.7) | 0.002 |
| Dementia |  |  |  |
| Vascular dementia | 22 (0.03) | 16 (0.02) | 0.005 |
| Dementia in other diseases classified elsewhere | 34 (0.05) | 28 (0.04) | 0.004 |
| Unspecified dementia | 47 (0.07) | 30 (0.04) | 0.01 |
| Alzheimer disease | 14 (0.02) | 11 (0.02) | 0.003 |
| Frontotemporal dementia | 10 (0.01) | 10 (0.01) | 0 |
| Dementia with Lewy bodies | 0 (0.0) | 0 (0.0) | - |
| Neoplasms |  |  |  |
| Neoplasms (any) | 10315 (14.6) | 10305 (14.6) | 4.00E-04 |
| Malignant neoplasms of lymphoid; hematopoietic and related tissue | 451 (0.6) | 486 (0.7) | 0.006 |
| Organ transplant |  |  |  |
| Renal Transplantation Procedures | 187 (0.3) | 187 (0.3) | 0 |
| Liver Transplantation Procedures | 27 (0.04) | 26 (0.04) | 7.00E-04 |
| Psoriasis | 557 (0.8) | 566 (0.8) | 0.001 |
| Rheumatoid arthritis |  |  |  |
| Rheumatoid arthritis with rheumatoid factor | 155 (0.2) | 136 (0.2) | 0.006 |
| Other rheumatoid arthritis | 509 (0.7) | 505 (0.7) | 7.00E-04 |
| Systemic lupus erythematosus (SLE) | 359 (0.5) | 390 (0.6) | 0.006 |
| Disorders involving the immune mechanism | 1098 (1.6) | 1125 (1.6) | 0.003 |

**Table P** – Characteristics of the age 65+ & age 45-64 COVID-19 cohorts after propensity score matching**.** SMD = Standardised Mean Difference.

|  | **Age 65+** | **Age 45-64** | **SMD** |
| --- | --- | --- | --- |
| Number | 39042 | 39042 | - |
| DEMOGRAPHICS |  |  |  |
| Age; mean (SD); y | 73.0 (7.0) | 55.0 (5.6) | 2.8 |
| Sex; n (%) |  |  |  |
| Female | 20692 (53.0) | 20259 (51.9) | 0.02 |
| Male | 18259 (46.8) | 18709 (47.9) | 0.02 |
| Other | 91 (0.2) | 74 (0.2) | 0.009 |
| Race; n (%) |  |  |  |
| White | 24670 (63.2) | 24371 (62.4) | 0.02 |
| Black or African American | 6958 (17.8) | 7358 (18.8) | 0.03 |
| Asian | 1207 (3.1) | 1242 (3.2) | 0.005 |
| American Indian or Alaska Native | 120 (0.3) | 113 (0.3) | 0.003 |
| Native Hawaiian or Other Pacific Islander | 88 (0.2) | 88 (0.2) | 0 |
| Unknown | 5999 (15.4) | 5870 (15.0) | 0.009 |
| Ethnicity; n (%) |  |  |  |
| Hispanic or Latino | 4733 (12.1) | 4526 (11.6) | 0.02 |
| Not Hispanic of Latino | 22480 (57.6) | 22446 (57.5) | 0.002 |
| Unknown | 11829 (30.3) | 12070 (30.9) | 0.01 |
| Problems related to housing and economic circumstances; n (%) | 394 (1.0) | 418 (1.1) | 0.006 |
| COMORBIDITIES; n (%) |  |  |  |
| Overweight and obesity | 8144 (20.9) | 8016 (20.5) | 0.008 |
| Hypertensive disease | 20406 (52.3) | 20530 (52.6) | 0.006 |
| Diabetes mellitus |  |  |  |
| Type 1 diabetes mellitus | 1210 (3.1) | 1215 (3.1) | 7.00E-04 |
| Type 2 diabetes mellitus | 10386 (26.6) | 10369 (26.6) | 0.001 |
| Chronic lower respiratory diseases |  |  |  |
| Bronchitis; not specified as acute or chronic | 2300 (5.9) | 2180 (5.6) | 0.01 |
| Simple and mucopurulent chronic bronchitis | 236 (0.6) | 245 (0.6) | 0.003 |
| Unspecified chronic bronchitis | 311 (0.8) | 302 (0.8) | 0.003 |
| Emphysema | 957 (2.5) | 973 (2.5) | 0.003 |
| Other chronic obstructive pulmonary disease | 3060 (7.8) | 3042 (7.8) | 0.002 |
| Asthma | 3966 (10.2) | 3858 ( 9.9) | 0.009 |
| Bronchiectasis | 321 (0.8) | 300 (0.8) | 0.006 |
| Nicotine dependence | 2771 (7.1) | 2799 (7.2) | 0.003 |
| Psychiatric comorbidities |  |  |  |
| Substance misuse | 4066 (10.4) | 4046 (10.4) | 0.002 |
| Psychotic disorders | 797 (2.0) | 750 (1.9) | 0.009 |
| Mood disorders | 6305 (16.1) | 6164 (15.8) | 0.01 |
| Anxiety disorders | 7325 (18.8) | 7146 (18.3) | 0.01 |
| Heart disease |  |  |  |
| Ischemic heart diseases | 6205 (15.9) | 6238 (16.0) | 0.002 |
| Other forms of heart disease | 11276 (28.9) | 11397 (29.2) | 0.007 |
| Chronic kidney diseases |  |  |  |
| Chronic kidney disease (CKD) | 4360 (11.2) | 4320 (11.1) | 0.003 |
| Hypertensive chronic kidney disease | 2446 (6.3) | 2399 (6.1) | 0.005 |
| Chronic liver disease |  |  |  |
| Alcoholic liver disease | 254 (0.7) | 237 (0.6) | 0.006 |
| Hepatic failure; not elsewhere classified | 353 (0.9) | 364 (0.9) | 0.003 |
| Chronic hepatitis; not elsewhere classified | 71 (0.2) | 85 (0.2) | 0.008 |
| Fibrosis and cirrhosis of liver | 713 (1.8) | 713 (1.8) | 0 |
| Fatty (change of) liver; not elsewhere classified | 1709 (4.4) | 1676 (4.3) | 0.004 |
| Chronic passive congestion of liver | 356 (0.9) | 345 (0.9) | 0.003 |
| Portal hypertension | 291 (0.7) | 287 (0.7) | 0.001 |
| Other specified diseases of liver | 1355 (3.5) | 1340 (3.4) | 0.002 |
| Cerebral infarction | 1574 (4.0) | 1528 (3.9) | 0.006 |
| Dementia |  |  |  |
| Vascular dementia | 164 (0.4) | 120 (0.3) | 0.02 |
| Dementia in other diseases classified elsewhere | 226 (0.6) | 157 (0.4) | 0.03 |
| Unspecified dementia | 417 (1.1) | 342 (0.9) | 0.02 |
| Alzheimer disease | 114 (0.3) | 78 (0.2) | 0.02 |
| Frontotemporal dementia | 26 (0.07) | 18 (0.05) | 0.009 |
| Dementia with Lewy bodies | 15 (0.04) | 10 (0.03) | 0.007 |
| Neoplasms |  |  |  |
| Neoplasms (any) | 11387 (29.2) | 11155 (28.6) | 0.01 |
| Malignant neoplasms of lymphoid; hematopoietic and related tissue | 717 (1.8) | 703 (1.8) | 0.003 |
| Organ transplant |  |  |  |
| Renal Transplantation Procedures | 152 (0.4) | 153 (0.4) | 4.00E-04 |
| Liver Transplantation Procedures | 43 (0.1) | 41 (0.1) | 0.002 |
| Psoriasis | 574 (1.5) | 531 (1.4) | 0.009 |
| Rheumatoid arthritis |  |  |  |
| Rheumatoid arthritis with rheumatoid factor | 288 (0.7) | 258 (0.7) | 0.009 |
| Other rheumatoid arthritis | 911 (2.3) | 874 (2.2) | 0.006 |
| Systemic lupus erythematosus (SLE) | 250 (0.6) | 260 (0.7) | 0.003 |
| Disorders involving the immune mechanism | 1198 (3.1) | 1163 (3.0) | 0.005 |

**Table Q** – Characteristics of the age 22-44 & age 10-21 COVID-19 cohorts after propensity score matching**.** SMD = Standardised Mean Difference.

|  | **Age 22-44** | **Age 10-21** | **SMD** |
| --- | --- | --- | --- |
| Number | 29753 | 29753 | - |
| DEMOGRAPHICS |  |  |  |
| Age; mean (SD); y | 31.2 (6.6) | 16.9 (3.2) | 2.7 |
| Sex; n (%) |  |  |  |
| Female | 17077 (57.4) | 16515 (55.5) | 0.04 |
| Male | 12434 (41.8) | 13009 (43.7) | 0.04 |
| Other | 242 (0.8) | 229 (0.8) | 0.005 |
| Race; n (%) |  |  |  |
| White | 17840 (60.0) | 17422 (58.6) | 0.03 |
| Black or African American | 3955 (13.3) | 4168 (14.0) | 0.02 |
| Asian | 590 (2.0) | 575 (1.9) | 0.004 |
| American Indian or Alaska Native | 110 (0.4) | 111 (0.4) | 6.00E-04 |
| Native Hawaiian or Other Pacific Islander | 188 (0.6) | 164 (0.6) | 0.01 |
| Unknown | 7070 (23.8) | 7313 (24.6) | 0.02 |
| Ethnicity; n (%) |  |  |  |
| Hispanic or Latino | 4604 (15.5) | 4898 (16.5) | 0.03 |
| Not Hispanic of Latino | 16345 (54.9) | 15886 (53.4) | 0.03 |
| Unknown | 8804 (29.6) | 8969 (30.1) | 0.01 |
| Problems related to housing and economic circumstances; n (%) | 89 (0.3) | 108 (0.4) | 0.01 |
| COMORBIDITIES; n (%) |  |  |  |
| Overweight and obesity | 1861 (6.3) | 2153 (7.2) | 0.04 |
| Hypertensive disease | 592 (2.0) | 659 (2.2) | 0.02 |
| Diabetes mellitus |  |  |  |
| Type 1 diabetes mellitus | 232 (0.8) | 231 (0.8) | 4.00E-04 |
| Type 2 diabetes mellitus | 285 (1.0) | 322 (1.1) | 0.01 |
| Chronic lower respiratory diseases |  |  |  |
| Bronchitis; not specified as acute or chronic | 484 (1.6) | 508 (1.7) | 0.006 |
| Simple and mucopurulent chronic bronchitis | 10 (0.03) | 16 (0.05) | 0.01 |
| Unspecified chronic bronchitis | 10 (0.03) | 14 (0.05) | 0.007 |
| Emphysema | 10 (0.03) | 10 (0.03) | 0 |
| Other chronic obstructive pulmonary disease | 31 (0.1) | 34 (0.1) | 0.003 |
| Asthma | 2802 (9.4) | 2905 ( 9.8) | 0.01 |
| Bronchiectasis | 25 (0.08) | 25 (0.08) | 0 |
| Nicotine dependence | 584 (2.0) | 556 (1.9) | 0.007 |
| Psychiatric comorbidities |  |  |  |
| Substance misuse | 1184 (4.0) | 1133 (3.8) | 0.009 |
| Psychotic disorders | 132 (0.4) | 130 (0.4) | 0.001 |
| Mood disorders | 2356 (7.9) | 2400 (8.1) | 0.005 |
| Anxiety disorders | 3439 (11.6) | 3589 (12.1) | 0.02 |
| Heart disease |  |  |  |
| Ischemic heart diseases | 66 (0.2) | 71 (0.2) | 0.004 |
| Other forms of heart disease | 1233 (4.1) | 1284 (4.3) | 0.009 |
| Chronic kidney diseases |  |  |  |
| Chronic kidney disease (CKD) | 131 (0.4) | 141 (0.5) | 0.005 |
| Hypertensive chronic kidney disease | 39 (0.1) | 54 (0.2) | 0.01 |
| Chronic liver disease |  |  |  |
| Alcoholic liver disease | 10 (0.03) | 10 (0.03) | 0 |
| Hepatic failure; not elsewhere classified | 23 (0.08) | 22 (0.07) | 0.001 |
| Chronic hepatitis; not elsewhere classified | 10 (0.03) | 10 (0.03) | 0 |
| Fibrosis and cirrhosis of liver | 29 (0.1) | 24 (0.08) | 0.006 |
| Fatty (change of) liver; not elsewhere classified | 175 (0.6) | 200 (0.7) | 0.01 |
| Chronic passive congestion of liver | 27 (0.09) | 25 (0.08) | 0.002 |
| Portal hypertension | 15 (0.05) | 10 (0.03) | 0.008 |
| Other specified diseases of liver | 96 (0.3) | 99 (0.3) | 0.002 |
| Cerebral infarction | 36 (0.1) | 39 (0.1) | 0.003 |
| Dementia |  |  |  |
| Vascular dementia | 0 (0.0) | 10 (0.03) | 0.03 |
| Dementia in other diseases classified elsewhere | 10 (0.03) | 10 (0.03) | 0 |
| Unspecified dementia | 10 (0.03) | 10 (0.03) | 0 |
| Alzheimer disease | 10 (0.03) | 10 (0.03) | 0 |
| Frontotemporal dementia | 0 (0.0) | 0 (0.0) | NA |
| Dementia with Lewy bodies | 0 (0.0) | 0 (0.0) | NA |
| Neoplasms |  |  |  |
| Neoplasms (any) | 1284 (4.3) | 1276 (4.3) | 0.001 |
| Malignant neoplasms of lymphoid; hematopoietic and related tissue | 103 (0.3) | 121 (0.4) | 0.01 |
| Organ transplant |  |  |  |
| Renal Transplantation Procedures | 10 (0.03) | 17 (0.06) | 0.01 |
| Liver Transplantation Procedures | 10 (0.03) | 10 (0.03) | 0 |
| Psoriasis | 70 (0.2) | 70 (0.2) | 0 |
| Rheumatoid arthritis |  |  |  |
| Rheumatoid arthritis with rheumatoid factor | 10 (0.03) | 10 (0.03) | 0 |
| Other rheumatoid arthritis | 25 (0.08) | 28 (0.09) | 0.003 |
| Systemic lupus erythematosus (SLE) | 42 (0.1) | 47 (0.2) | 0.004 |
| Disorders involving the immune mechanism | 243 (0.8) | 254 (0.9) | 0.004 |

**Table R** – Characteristics of COVID-19 cohorts requiring and not requiring hospitalisation, after propensity score matching**.** SMD = Standardised Mean Difference.

|  | **Hospitalised patients** | **Non Hospitalised patients** | **SMD** |
| --- | --- | --- | --- |
| Number | 52597 | 52597 | - |
| DEMOGRAPHICS |  |  |  |
| Age; mean (SD); y | 56.6 (18.6) | 57.2 (18.2) | 0.03 |
| Sex; n (%) |  |  |  |
| Female | 26940 (51.2) | 27902 (53.0) | 0.04 |
| Male | 25579 (48.6) | 24660 (46.9) | 0.03 |
| Other | 78 (0.1) | 35 (0.07) | 0.02 |
| Race; n (%) |  |  |  |
| White | 29633 (56.3) | 29885 (56.8) | 0.01 |
| Black or African American | 11357 (21.6) | 11529 (21.9) | 0.008 |
| Asian | 1814 (3.4) | 1973 (3.8) | 0.02 |
| American Indian or Alaska Native | 263 (0.5) | 286 (0.5) | 0.006 |
| Native Hawaiian or Other Pacific Islander | 172 (0.3) | 162 (0.3) | 0.003 |
| Unknown | 9358 (17.8) | 8762 (16.7) | 0.03 |
| Ethnicity; n (%) |  |  |  |
| Hispanic or Latino | 9740 (18.5) | 10210 (19.4) | 0.02 |
| Not Hispanic of Latino | 29987 (57.0) | 30741 (58.4) | 0.03 |
| Unknown | 12870 (24.5) | 11646 (22.1) | 0.06 |
| Problems related to housing and economic circumstances; n (%) | 1045 (2.0) | 1022 (1.9) | 0.003 |
| COMORBIDITIES; n (%) |  |  |  |
| Overweight and obesity | 14199 (27.0) | 15004 (28.5) | 0.03 |
| Hypertensive disease | 26350 (50.1) | 27194 (51.7) | 0.03 |
| Diabetes mellitus |  |  |  |
| Type 1 diabetes mellitus | 2041 (3.9) | 1995 (3.8) | 0.005 |
| Type 2 diabetes mellitus | 15366 (29.2) | 15438 (29.4) | 0.003 |
| Chronic lower respiratory diseases |  |  |  |
| Bronchitis; not specified as acute or chronic | 2505 (4.8) | 2609 (5.0) | 0.009 |
| Simple and mucopurulent chronic bronchitis | 334 (0.6) | 357 (0.7) | 0.005 |
| Unspecified chronic bronchitis | 482 (0.9) | 455 (0.9) | 0.005 |
| Emphysema | 1612 (3.1) | 1546 (2.9) | 0.007 |
| Other chronic obstructive pulmonary disease | 5105 ( 9.7) | 4718 (9.0) | 0.03 |
| Asthma | 6006 (11.4) | 6464 (12.3) | 0.03 |
| Bronchiectasis | 496 (0.9) | 494 (0.9) | 4.00E-04 |
| Nicotine dependence | 4913 (9.3) | 4983 (9.5) | 0.005 |
| Psychiatric comorbidities |  |  |  |
| Substance misuse | 7396 (14.1) | 7572 (14.4) | 0.01 |
| Psychotic disorders | 1734 (3.3) | 1598 (3.0) | 0.01 |
| Mood disorders | 9857 (18.7) | 10189 (19.4) | 0.02 |
| Anxiety disorders | 10894 (20.7) | 11312 (21.5) | 0.02 |
| Heart disease |  |  |  |
| Ischemic heart diseases | 9886 (18.8) | 9458 (18.0) | 0.02 |
| Other forms of heart disease | 17867 (34.0) | 17641 (33.5) | 0.009 |
| Chronic kidney diseases |  |  |  |
| Chronic kidney disease (CKD) | 7697 (14.6) | 6960 (13.2) | 0.04 |
| Hypertensive chronic kidney disease | 4506 (8.6) | 3973 (7.6) | 0.04 |
| Chronic liver disease |  |  |  |
| Alcoholic liver disease | 519 (1.0) | 467 (0.9) | 0.01 |
| Hepatic failure; not elsewhere classified | 745 (1.4) | 611 (1.2) | 0.02 |
| Chronic hepatitis; not elsewhere classified | 115 (0.2) | 112 (0.2) | 0.001 |
| Fibrosis and cirrhosis of liver | 1129 (2.1) | 1034 (2.0) | 0.01 |
| Fatty (change of) liver; not elsewhere classified | 2618 (5.0) | 2769 (5.3) | 0.01 |
| Chronic passive congestion of liver | 431 (0.8) | 435 (0.8) | 8.00E-04 |
| Portal hypertension | 514 (1.0) | 446 (0.8) | 0.01 |
| Other specified diseases of liver | 1717 (3.3) | 1754 (3.3) | 0.004 |
| Cerebral infarction | 2740 (5.2) | 2551 (4.8) | 0.02 |
| Dementia |  |  |  |
| Vascular dementia | 657 (1.2) | 563 (1.1) | 0.02 |
| Dementia in other diseases classified elsewhere | 1030 (2.0) | 946 (1.8) | 0.01 |
| Unspecified dementia | 2423 (4.6) | 2051 (3.9) | 0.04 |
| Alzheimer disease | 822 (1.6) | 781 (1.5) | 0.006 |
| Frontotemporal dementia | 49 (0.09) | 41 (0.08) | 0.005 |
| Dementia with Lewy bodies | 61 (0.1) | 55 (0.1) | 0.003 |
| Neoplasms |  |  |  |
| Neoplasms (any) | 12183 (23.2) | 12867 (24.5) | 0.03 |
| Malignant neoplasms of lymphoid; hematopoietic and related tissue | 1111 (2.1) | 1110 (2.1) | 1.00E-04 |
| Organ transplant |  |  |  |
| Renal Transplantation Procedures | 363 (0.7) | 323 (0.6) | 0.009 |
| Liver Transplantation Procedures | 80 (0.2) | 65 (0.1) | 0.008 |
| Psoriasis | 629 (1.2) | 677 (1.3) | 0.008 |
| Rheumatoid arthritis |  |  |  |
| Rheumatoid arthritis with rheumatoid factor | 276 (0.5) | 289 (0.5) | 0.003 |
| Other rheumatoid arthritis | 1128 (2.1) | 1170 (2.2) | 0.005 |
| Systemic lupus erythematosus (SLE) | 423 (0.8) | 414 (0.8) | 0.002 |
| Disorders involving the immune mechanism | 1905 (3.6) | 1878 (3.6) | 0.003 |

**Table S** – Characteristics of COVID-19 cohorts requiring and not requiring intensive treatment unit (ITU) admission, after propensity score matching**.** SMD = Standardised Mean Difference.

|  | **ITU admission** | **No ITU admission** | **SMD** |
| --- | --- | --- | --- |
| Number | 10378 | 10378 | - |
| DEMOGRAPHICS |  |  |  |
| Age; mean (SD); y | 59.7 (17.4) | 60.7 (17.3) | 0.06 |
| Sex; n (%) |  |  |  |
| Female | 4362 (42.0) | 4440 (42.8) | 0.02 |
| Male | 6010 (57.9) | 5935 (57.2) | 0.01 |
| Other | 10 (0.1) | 10 (0.1) | 0 |
| Race; n (%) |  |  |  |
| White | 5998 (57.8) | 5991 (57.7) | 0.001 |
| Black or African American | 2289 (22.1) | 2327 (22.4) | 0.009 |
| Asian | 355 (3.4) | 377 (3.6) | 0.01 |
| American Indian or Alaska Native | 50 (0.5) | 54 (0.5) | 0.005 |
| Native Hawaiian or Other Pacific Islander | 38 (0.4) | 46 (0.4) | 0.01 |
| Unknown | 1648 (15.9) | 1583 (15.3) | 0.02 |
| Ethnicity; n (%) |  |  |  |
| Hispanic or Latino | 2347 (22.6) | 2390 (23.0) | 0.01 |
| Not Hispanic of Latino | 6092 (58.7) | 6130 (59.1) | 0.007 |
| Unknown | 1939 (18.7) | 1858 (17.9) | 0.02 |
| Problems related to housing and economic circumstances; n (%) | 265 (2.6) | 243 (2.3) | 0.01 |
| COMORBIDITIES; n (%) |  |  |  |
| Overweight and obesity | 3578 (34.5) | 3688 (35.5) | 0.02 |
| Hypertensive disease | 6597 (63.6) | 6729 (64.8) | 0.03 |
| Diabetes mellitus |  |  |  |
| Type 1 diabetes mellitus | 725 (7.0) | 645 (6.2) | 0.03 |
| Type 2 diabetes mellitus | 4448 (42.9) | 4433 (42.7) | 0.003 |
| Chronic lower respiratory diseases |  |  |  |
| Bronchitis; not specified as acute or chronic | 664 (6.4) | 659 (6.3) | 0.002 |
| Simple and mucopurulent chronic bronchitis | 106 (1.0) | 97 (0.9) | 0.009 |
| Unspecified chronic bronchitis | 139 (1.3) | 115 (1.1) | 0.02 |
| Emphysema | 553 (5.3) | 470 (4.5) | 0.04 |
| Other chronic obstructive pulmonary disease | 1588 (15.3) | 1463 (14.1) | 0.03 |
| Asthma | 1376 (13.3) | 1344 (12.9) | 0.009 |
| Bronchiectasis | 159 (1.5) | 158 (1.5) | 8.00E-04 |
| Nicotine dependence | 1280 (12.3) | 1270 (12.2) | 0.003 |
| Psychiatric comorbidities |  |  |  |
| Substance misuse | 1928 (18.6) | 1897 (18.3) | 0.008 |
| Psychotic disorders | 461 (4.4) | 456 (4.4) | 0.002 |
| Mood disorders | 2395 (23.1) | 2462 (23.7) | 0.02 |
| Anxiety disorders | 2729 (26.3) | 2776 (26.7) | 0.01 |
| Heart disease |  |  |  |
| Ischemic heart diseases | 3012 (29.0) | 2853 (27.5) | 0.03 |
| Other forms of heart disease | 5610 (54.1) | 5593 (53.9) | 0.003 |
| Chronic kidney diseases |  |  |  |
| Chronic kidney disease (CKD) | 2298 (22.1) | 2185 (21.1) | 0.03 |
| Hypertensive chronic kidney disease | 1446 (13.9) | 1337 (12.9) | 0.03 |
| Chronic liver disease |  |  |  |
| Alcoholic liver disease | 161 (1.6) | 143 (1.4) | 0.01 |
| Hepatic failure; not elsewhere classified | 365 (3.5) | 262 (2.5) | 0.06 |
| Chronic hepatitis; not elsewhere classified | 39 (0.4) | 29 (0.3) | 0.02 |
| Fibrosis and cirrhosis of liver | 353 (3.4) | 267 (2.6) | 0.05 |
| Fatty (change of) liver; not elsewhere classified | 686 (6.6) | 746 (7.2) | 0.02 |
| Chronic passive congestion of liver | 132 (1.3) | 128 (1.2) | 0.003 |
| Portal hypertension | 155 (1.5) | 124 (1.2) | 0.03 |
| Other specified diseases of liver | 469 (4.5) | 474 (4.6) | 0.002 |
| Cerebral infarction | 941 (9.1) | 867 (8.4) | 0.03 |
| Dementia |  |  |  |
| Vascular dementia | 168 (1.6) | 175 (1.7) | 0.005 |
| Dementia in other diseases classified elsewhere | 248 (2.4) | 260 (2.5) | 0.007 |
| Unspecified dementia | 646 (6.2) | 641 (6.2) | 0.002 |
| Alzheimer disease | 159 (1.5) | 187 (1.8) | 0.02 |
| Frontotemporal dementia | 12 (0.1) | 10 (0.1) | 0.006 |
| Dementia with Lewy bodies | 14 (0.1) | 20 (0.2) | 0.01 |
| Neoplasms |  |  |  |
| Neoplasms (any) | 2794 (26.9) | 2877 (27.7) | 0.02 |
| Malignant neoplasms of lymphoid; hematopoietic and related tissue | 298 (2.9) | 302 (2.9) | 0.002 |
| Organ transplant |  |  |  |
| Renal Transplantation Procedures | 111 (1.1) | 90 (0.9) | 0.02 |
| Liver Transplantation Procedures | 23 (0.2) | 20 (0.2) | 0.006 |
| Psoriasis | 182 (1.8) | 182 (1.8) | 0 |
| Rheumatoid arthritis |  |  |  |
| Rheumatoid arthritis with rheumatoid factor | 75 (0.7) | 81 (0.8) | 0.007 |
| Other rheumatoid arthritis | 277 (2.7) | 295 (2.8) | 0.01 |
| Systemic lupus erythematosus (SLE) | 88 (0.8) | 99 (1.0) | 0.01 |
| Disorders involving the immune mechanism | 457 (4.4) | 429 (4.1) | 0.01 |

**Table T** – Characteristics of leukocytosis and non-leukocytosis COVID-19 cohorts after propensity score matching**.** SMD = Standardised Mean Difference.

|  | **Leukocytosis** | **No leukocytosis** | **SMD** |
| --- | --- | --- | --- |
| Number | 25417 | 25417 | - |
| DEMOGRAPHICS |  |  |  |
| Age; mean (SD); y | 57.0 (19.1) | 57.0 (18.6) | 5.00E-04 |
| Sex; n (%) |  |  |  |
| Female | 12459 (49.0) | 12428 (48.9) | 0.002 |
| Male | 12923 (50.8) | 12964 (51.0) | 0.003 |
| Other | 35 (0.1) | 25 (0.1) | 0.01 |
| Race; n (%) |  |  |  |
| White | 14561 (57.3) | 14669 (57.7) | 0.009 |
| Black or African American | 5592 (22.0) | 5434 (21.4) | 0.02 |
| Asian | 1015 (4.0) | 1058 (4.2) | 0.009 |
| American Indian or Alaska Native | 155 (0.6) | 155 (0.6) | 0 |
| Native Hawaiian or Other Pacific Islander | 70 (0.3) | 75 (0.3) | 0.004 |
| Unknown | 4024 (15.8) | 4026 (15.8) | 2.00E-04 |
| Ethnicity; n (%) |  |  |  |
| Hispanic or Latino | 4323 (17.0) | 4544 (17.9) | 0.02 |
| Not Hispanic of Latino | 15107 (59.4) | 14878 (58.5) | 0.02 |
| Unknown | 5987 (23.6) | 5995 (23.6) | 7.00E-04 |
| Problems related to housing and economic circumstances; n (%) | 550 (2.2) | 534 (2.1) | 0.004 |
| COMORBIDITIES; n (%) |  |  |  |
| Overweight and obesity | 7921 (31.2) | 7809 (30.7) | 0.01 |
| Hypertensive disease | 14099 (55.5) | 14089 (55.4) | 8.00E-04 |
| Diabetes mellitus |  |  |  |
| Type 1 diabetes mellitus | 1042 (4.1) | 1028 (4.0) | 0.003 |
| Type 2 diabetes mellitus | 8420 (33.1) | 8442 (33.2) | 0.002 |
| Chronic lower respiratory diseases |  |  |  |
| Bronchitis; not specified as acute or chronic | 1466 (5.8) | 1417 (5.6) | 0.008 |
| Simple and mucopurulent chronic bronchitis | 225 (0.9) | 226 (0.9) | 4.00E-04 |
| Unspecified chronic bronchitis | 271 (1.1) | 262 (1.0) | 0.003 |
| Emphysema | 1068 (4.2) | 1018 (4.0) | 0.01 |
| Other chronic obstructive pulmonary disease | 3405 (13.4) | 3323 (13.1) | 0.01 |
| Asthma | 3431 (13.5) | 3382 (13.3) | 0.006 |
| Bronchiectasis | 292 (1.1) | 287 (1.1) | 0.002 |
| Nicotine dependence | 3137 (12.3) | 3139 (12.3) | 2.00E-04 |
| Psychiatric comorbidities |  |  |  |
| Substance misuse | 4463 (17.6) | 4490 (17.7) | 0.003 |
| Psychotic disorders | 882 (3.5) | 872 (3.4) | 0.002 |
| Mood disorders | 5350 (21.0) | 5233 (20.6) | 0.01 |
| Anxiety disorders | 6131 (24.1) | 6011 (23.6) | 0.01 |
| Heart disease |  |  |  |
| Ischemic heart diseases | 5772 (22.7) | 5738 (22.6) | 0.003 |
| Other forms of heart disease | 10361 (40.8) | 10345 (40.7) | 0.001 |
| Chronic kidney diseases |  |  |  |
| Chronic kidney disease (CKD) | 4458 (17.5) | 4427 (17.4) | 0.003 |
| Hypertensive chronic kidney disease | 2751 (10.8) | 2754 (10.8) | 4.00E-04 |
| Chronic liver disease |  |  |  |
| Alcoholic liver disease | 238 (0.9) | 254 (1.0) | 0.006 |
| Hepatic failure; not elsewhere classified | 495 (1.9) | 434 (1.7) | 0.02 |
| Chronic hepatitis; not elsewhere classified | 44 (0.2) | 47 (0.2) | 0.003 |
| Fibrosis and cirrhosis of liver | 497 (2.0) | 519 (2.0) | 0.006 |
| Fatty (change of) liver; not elsewhere classified | 1308 (5.1) | 1285 (5.1) | 0.004 |
| Chronic passive congestion of liver | 197 (0.8) | 197 (0.8) | 0 |
| Portal hypertension | 213 (0.8) | 223 (0.9) | 0.004 |
| Other specified diseases of liver | 773 (3.0) | 769 (3.0) | 9.00E-04 |
| Cerebral infarction | 1591 (6.3) | 1557 (6.1) | 0.006 |
| Dementia |  |  |  |
| Vascular dementia | 365 (1.4) | 368 (1.4) | 0.001 |
| Dementia in other diseases classified elsewhere | 578 (2.3) | 578 (2.3) | 0 |
| Unspecified dementia | 1356 (5.3) | 1374 (5.4) | 0.003 |
| Alzheimer disease | 405 (1.6) | 410 (1.6) | 0.002 |
| Frontotemporal dementia | 23 (0.09) | 26 (0.1) | 0.004 |
| Dementia with Lewy bodies | 33 (0.1) | 32 (0.1) | 0.001 |
| Neoplasms |  |  |  |
| Neoplasms (any) | 5831 (22.9) | 5782 (22.7) | 0.005 |
| Malignant neoplasms of lymphoid; hematopoietic and related tissue | 599 (2.4) | 579 (2.3) | 0.005 |
| Organ transplant |  |  |  |
| Renal Transplantation Procedures | 142 (0.6) | 150 (0.6) | 0.004 |
| Liver Transplantation Procedures | 23 (0.09) | 27 (0.1) | 0.005 |
| Psoriasis | 349 (1.4) | 323 (1.3) | 0.009 |
| Rheumatoid arthritis |  |  |  |
| Rheumatoid arthritis with rheumatoid factor | 155 (0.6) | 141 (0.6) | 0.007 |
| Other rheumatoid arthritis | 603 (2.4) | 577 (2.3) | 0.007 |
| Systemic lupus erythematosus (SLE) | 210 (0.8) | 197 (0.8) | 0.006 |
| Disorders involving the immune mechanism | 896 (3.5) | 866 (3.4) | 0.006 |

**Table U** – Mean count number of occurrences of each and any long-COVID feature among patients who have them recorded at least once, in the 6-months after a diagnosis of COVID-19 or influenza (using matched cohorts). The p-value tests the hypothesis that the counts are equal between the cohorts.

|  | **COVID-19** | **Influenza** | **p-value** |
| --- | --- | --- | --- |
|  | **Mean count (95% CI)** | **Mean count (95% CI)** | **(Poisson regression)** |
| **Anxiety/Depression** | 3.08 (3.06-3.11) | 2.84 (2.81-2.87) | <0.0001 |
| **Chest/Throat Pain** | 1.83 (1.80-1.85) | 1.78 (1.74-1.81) | 0.043 |
| **Abnormal Breathing** | 2.04 (2.01-2.06) | 1.92 (1.89-1.95) | <0.0001 |
| **Myalgia** | 1.66 (1.60-1.71) | 1.64 (1.58-1.70) | 0.77 |
| **Fatigue** | 1.88 (1.85-1.91) | 1.72 (1.68-1.75) | <0.0001 |
| **Headache** | 2.30 (2.26-2.33) | 1.99 (1.96-2.03) | <0.0001 |
| **Abdominal symptoms** | 2.07 (2.04-2.10) | 1.98 (1.95-2.01) | <0.0001 |
| **Cognitive symptoms** | 2.75 (2.70-2.81) | 2.44 (2.38-2.50) | <0.0001 |
| **Pain** | 2.46 (2.42-2.50) | 2.43 (2.39-2.47) | 0.26 |
| **Any** | 3.34 (3.32-3.35) | 3.07 (3.05-3.09) | <0.0001 |

**Table V** – Comparison in the 6-month incidence of any pain, between patients with COVID-19 and a matched cohort of patients with influenza. Any pain in this analysis refers to the composite endpoint of chest/throat pain, headache, myalgia, other pain (as defined in Supplementary Methods 4) or abdominal and pelvic pain (a subcategory of the abdominal symptoms also defined in Supplementary Methods 4).

| **Patients with COVID-19** | **Matched patients with influenza** | **Comparison** | |
| --- | --- | --- | --- |
| **6-month incidence, % (95% CI)** | **6-month incidence, % (95% CI)** | **HR (95% CI)** | **p-value** |
| 34.15 (33.64-34.65) | 23.98 (23.67-24.29) | 1.53 (1.49-1.56) | < 0.0001 |

# References

1. Taquet M, Luciano S, Geddes JR, Harrison PJ. Bidirectional associations between COVID-19 and psychiatric disorder: retrospective cohort studies of 62 354 COVID-19 cases in the USA. Lancet Psychiatry. 2021;8: 130–140.

2. Taquet M, Geddes JR, Husain M, Luciano S, Harrison PJ. 6-month neurological and psychiatric outcomes in 236 379 survivors of COVID-19: a retrospective cohort study using electronic health records. Lancet Psychiatry. 2021;8: 416–427.

3. Casey JA, Schwartz BS, Stewart WF, Adler NE. Using Electronic Health Records for Population Health Research: A Review of Methods and Applications. Annu Rev Public Health. 2016;37: 61–81.

4. Cowie MR, Blomster JI, Curtis LH, Duclaux S, Ford I, Fritz F, et al. Electronic health records to facilitate clinical research. Clin Res Cardiol. 2017;106: 1–9.

5. Jetley G, Zhang H. Electronic health records in IS research: Quality issues, essential thresholds and remedial actions. Decis Support Syst. 2019;126: 113137.

6. de Lusignan S, Dorward J, Correa A, Jones N, Akinyemi O, Amirthalingam G, et al. Risk factors for SARS-CoV-2 among patients in the Oxford Royal College of General Practitioners Research and Surveillance Centre primary care network: a cross-sectional study. Lancet Infect Dis. 2020. doi:10.1016/S1473-3099(20)30371-6

7. Zhang J-J, Dong X, Cao Y-Y, Yuan Y-D, Yang Y-B, Yan Y-Q, et al. Clinical characteristics of 140 patients infected with SARS-CoV-2 in Wuhan, China. Allergy. 2020. Available: https://onlinelibrary.wiley.com/doi/abs/10.1111/all.14238

8. Chen N, Zhou M, Dong X, Qu J, Gong F, Han Y, et al. Epidemiological and clinical characteristics of 99 cases of 2019 novel coronavirus pneumonia in Wuhan, China: a descriptive study. Lancet. 2020;395: 507–513.

9. Wang QQ, Kaelber DC, Xu R, Volkow ND. COVID-19 risk and outcomes in patients with substance use disorders: analyses from electronic health records in the United States. Mol Psychiatry. 2021;26: 30–39.

10. Williamson EJ, Walker AJ, Bhaskaran K, Bacon S, Bates C, Morton CE, et al. Factors associated with COVID-19-related death using OpenSAFELY. Nature; 584: 430–436.

11. Grambsch PM, Therneau TM. Proportional hazards tests and diagnostics based on weighted residuals. Biometrika. 1994;81: 515.

12. Royston P, Parmar MKB. Flexible parametric proportional-hazards and proportional-odds models for censored survival data, with application to prognostic modelling and estimation of treatment effects. Statistics in Medicine. 2002. pp. 2175–2197. doi:10.1002/sim.1203

13. Liu X-R, Pawitan Y, Clements M. Parametric and penalized generalized survival models. Stat Methods Med Res. 2018;27: 1531–1546.

14. Dice LR. Measures of the amount of ecologic association between species. Ecology. 1945;26: 297–302.

15. Efron B, Gong G. A leisurely look at the bootstrap, the jackknife, and cross-validation. Am Stat. 1983;37: 36–48.
