# Supplementary material for: Incidence, co-occurrence, and evolution of long-COVID features: A 6-month retrospective cohort study of 273,618 survivors of COVID-19
Source: PLoS Med. 2021 Sep 28;18(9):e1003773. doi: 10.1371/journal.pmed.1003773 (PMC8478214; doi:10.1371/journal.pmed.1003773)
Supplement: S1 Text — Supporting Methods A. TriNetX network. Supporting Methods B. Definition of cohorts. Supporting Methods C. Definition of covariates. Supporting Methods D. Definition of outcomes. Supporting Methods E. Details on statistical analyses. Supporting Methods F. Details on secondary analyses. (DOCX) [file pmed.1003773.s004.docx]

# Supporting Methods

## A TriNetX network

This section provides an expanded version of our previous description of the network [1,2].

*Legal and ethical status*

TriNetX’s Analytics network is compliant with the Health Insurance Portability and Accountability Act (HIPAA), the US federal law which protects the privacy and security of healthcare data. TriNetX is certified to the ISO 27001:2013 standard and maintains an Information Security Management System (ISMS) to ensure the protection of the healthcare data it has access to and to meet the requirements of the HIPAA Security Rule. Any data displayed on the TriNetX Platform in aggregate form, or any patient level data provided in a data set generated by the TriNetX Platform, only contains de-identified data as per the de-identification standard defined in Section §164.514(a) of the HIPAA Privacy Rule. The process by which the data is de-identified is attested to through a formal determination by a qualified expert as defined in Section §164.514(b)(1) of the HIPAA Privacy Rule. This formal determination by a qualified expert, refreshed in December 2020, supersedes the need for TriNetX’s previous waiver from the Western Institutional Review Board (IRB). The network contains data that are provided by participating Health Care Organizations (HCOs), each of which represents and warrants that it has all necessary rights, consents, approvals and authority to provide the data to TriNetX under a Business Associate Agreement (BAA), so long as their name remains anonymous as a data source and their data are utilized for research purposes. The data shared through the TriNetX Platform are attenuated to ensure that they do not include sufficient information to facilitate the determination of which HCO contributed which specific information about a patient.

*Acquisition of data, quality control, and other procedures*

The data are stored onboard a TriNetX appliance – a physical server residing at the institution’s data centre or a virtual hosted appliance. The TriNetX platform is a fleet of these appliances connected into a federated network able to broadcast queries to each appliance. Results are subsequently collected and aggregated.

Once the data are sent to the network, they are mapped to a standard and controlled set of clinical terminologies and undergo a data quality assessment including ‘data cleaning’ that rejects records which do not meet the TriNetX quality standards. HIPAA compliance of the clinical patient data is achieved using de-identification. Different data modalities are available in the network. They include demographics (coded to HL7 version 3 administrative standards), diagnoses (represented by ICD-10-CM codes), procedures (coded in ICD-10-PCS or CPT), and measurements (coded to LOINC). While extensive information is provided about patients’ diagnoses and procedures, other variables (such as socioeconomic and lifetime factors) are not comprehensively represented.

The data from a typical HCO generally go back around 7 years, with some going back 13 years. The data are continuously updated. HCOs update their data at various times, with most refreshing every 1, 2, or 4 weeks.

The data come primarily (>93%) from HCOs in the USA, with the remainder coming from India, Australia, Malaysia, Taiwan, Spain, UK, and Bulgaria. As noted above, to comply with legal frameworks and ethical guidelines guarding against data re-identification, the identity of participating HCOs and their individual contribution to each dataset are not disclosed to researchers.

Data quality assessment followed a standardised strategy wherein the data are reviewed for conformance (adherence to specified standards and formats), completeness (quantifying data presence or absence) and plausibility (believability of the data from a clinical perspective). There are pre-defined metrics for each of the above assessment categories. Results for these metrics are visualised and reviewed for each new site that joins the network as well as on an ongoing basis. Any identified issue is communicated to the data provider and resolved before continuing data collection.

The basic formatting of contributed data is also checked (e.g. to ensure that dates are properly represented). Records are checked against a list of required fields (e.g., patient identifier) and rejects those records for which the required information is missing. Referential integrity checking is done to ensure that data spanning multiple database tables can be successfully joined together. As the data are refreshed, changes in volume of data over time is monitored to ensure data validity. At least one non-demographic fact for each patient is required for them to be counted in the dataset. Patient records with only demographics information are discarded.

The software also undergoes quality control. The engineers testing the software are independent from the engineers developing it. Each test code is checked by two independent testing engineers. Each piece of software is tested extensively against a range of synthetic data (i.e. generated for the purpose of testing) for which the expected output is established independently. If the software fails to return this output, then the software is deemed to have failed the test and is examined and modified accordingly. For statistical software (including that used for propensity score matching, for Kaplan-Meier analysis, etc), an additional quality control step is implemented. Two independent codes are written in two different programming languages (typically R and python) and the statistical results are compared. If discrepancies are identified, then the codes are deemed to have failed the test and are examined and modified accordingly. All the code is reviewed independently by another engineer.

The test strategy follows three levels of granularity:

1. Unit tests: These test specific blocks, or units, of code that perform specific actions (e.g. querying the database).
2. Integration tests: These ensure that different components are working together correctly.
3. End-to-end tests: These tests run the entire system and check the final output.

*Some comments on advantages and disadvantages of EHR data*

One advantage of EHR data, like those in TriNetX, over insurance claim data is that both insured and uninsured patients are included. An advantage of EHR data over survey data is that they represent the diagnostic rates in the population presenting to healthcare facilities. This provides an accurate account of the burden of specific diagnoses on healthcare systems. However, there are also limitations inherent to research using electronic health records [3–5], including TriNetX:

1. Undiagnosed patients who might have features of long-COVID but did not seek medical attention (or in whom the diagnosis was missed) are not included leading to underestimation of actual incidences.
2. Despite the matching and use of various comparison cohorts, there may well be residual confounding, particularly related to social and economic factors which are not well captured in EHR networks and which might influence outcomes post COVID-19.
3. We do not know which diagnoses were made in primary or secondary care or specialist facilities, nor by whom.
4. A patient may be seen in different HCOs for different parts of their care, and if one HCO is not part of the federated network then part of their medical records may not be available. Using a network of HCOs (rather than a single HCO) limits this possibility but does not fully remove it.
5. How long a symptom/diagnosis persists is difficult to assess using EHR data as this is not typically coded. As a result, we can comment on incidence of new cases but cannot assess the duration of clinical features.
6. Since the data are presented as they are recorded, we cannot be sure that there has not been mis-recording of information, adding a degree of noise to the data.
7. Historical data before the start of EHRs (or the addition of an HCO to the network) may well be incomplete.

## B Definition of cohorts

The control cohort used consisted of patients with a diagnosis of influenza. Specifically, patients with influenza were those who had any of the following diagnoses:

- J09: Influenza due to certain identified influenza viruses
- J10: Influenza due to other identified influenza virus
- J11: Influenza due to unidentified influenza virus.

Because some patients with the control index event might have had COVID-19 at a different point in time, we excluded from the control cohorts all those who had COVID-19 at any point in time. To avoid any contamination between cohorts, COVID-19 as an exclusion criterion was defined in the broader sense to be all patients with a confirmed diagnosis of COVID-19 (ICD-10 code U07.1) but also patients with an unconfirmed COVID-19 diagnosis (U07.2), a recorded positive PCR test for COVID-19, or any of the following recorded on or after January 20, 2020: Pneumonia due to SARS-associated coronavirus (J12.81), Other coronavirus as the cause of disease classified elsewhere (B97.29), or Coronavirus infection unspecified (B34.2). Inclusion of the latter three diagnostic codes captures patients who receive a COVID-19 diagnosis in the early stage of the pandemic when the ICD code for COVID-19 (U07) was not yet defined. Specifically, the following codes were excluded from the control cohort if they occurred on or after January 20, 2020:

- U07.1: COVID-19, virus identified
- U07.2: COVID-19, virus not identified
- J12.81: Pneumonia due to SARS-associated coronavirus
- B97.29: Other coronavirus as the cause of disease classified elsewhere
- B34.2: Coronavirus infection, unspecified
- Positive SARS-CoV-2 RNA in Respiratory specimen
- Positive SARS-CoV-2 RNA in Unspecified specimen
- Positive SARS-CoV-2 N gene in Respiratory specimen
- Positive SARS-CoV-2 N gene in Unspecified specimen
- Positive SARS-CoV-2 RdRp gene in Respiratory specimen
- Positive SARS-CoV-2 E gene in Respiratory specimen
- Positive SARS-CoV-2 E gene in Unspecified specimen
- Positive SARS-CoV-2 RNA panel in Respiratory specimen
- Positive SARS-CoV-2 RNA panel in Unspecified specimen
- Positive SARS-CoV-2 RNA in Nasopharynx
- Positive SARS coronavirus 2 and related RNA
- Positive SARS-related coronavirus RNA in Respiratory specimen
- Positive SARS coronavirus 2 ORF1ab in Respiratory specimen

The duration of follow-up of the patients depended on when they had the index event. Patients who had the index events more than 6 months before the date of the analysis (December 16, 2020) had 6 months of follow-up. The other patients were followed up until December 16, 2020. The Kaplan-Meier estimator accommodates differences in duration of follow-up by means of censoring.

To keep the cohorts as homogeneous as possible (and because sample size was not an issue), we included fewer patients in the cohort of interest (by only including those with a diagnostic code for COVID-19), and excluded more patients from the control cohort (by excluding both those with a diagnostic code and those with a positive tests). Making the cohorts more homogeneous decreases the sensitivity of the findings to bias even when it comes at the price of a smaller sample size (for an excellent discussion of that point, see Rosenbaum P. Observation and experiment. Chapter 10. Harvard University Press; 2018 Nov 5).

There are both clinical and statistical reasons to exclude patients who had died during the study period.

Clinically, if a patient receives one of the codes for the features of long COVID but then dies later on, it is possible that they had a misdiagnosed critical health event which can hardly be attributed to long-COVID. For instance, if a patient sees a clinician with shortness of breath, receives a code for “abnormal breathing”, but then dies, it is possible that they had a misdiagnosed pulmonary embolism. The same applies to most features of long COVID investigated (e.g. abdominal symptoms being misdiagnosed abdominal aortic aneurysm, chest pain being misdiagnosed myocardial infarction, etc). One exception perhaps are psychiatric symptoms which might lead to death by suicide even as part of a long-COVID clinical picture. However, the impact of including or excluding people who had died on the 6-month psychiatric outcomes has already been investigated in a previous study and was found to be minimal [2].

Statistically, including patients who had died introduces the issue of competing risks which violates one key assumption of standard survival analysis: that the event may occur following the censoring event. While models for competing risks exist, because of the first reason above, we preferred to use a standard survival analysis while excluding patients who had died.

## C Definition of covariates

To reduce the effect of confounding on associations between a diagnosis of COVID-19 and a subsequent clinical feature of long-COVID, cohorts were matched for established or suspected risk factors for COVID-19 [6–9] and for established risk factors for COVID-19 death [10] (taken to be risk factors of a more severe COVID-19 illness). The following confounding factors were therefore included (with ICD-10/CDC codes in brackets):

1. **Age** at the time of diagnosis.
2. **Sex** coded as female, male, or other.
3. **Race** encoded as 6 separate dichotomous variables: White (2106-3), Black or African American (2054-5), American Indian or Alaska Native (1002-5), Asian (2028-9), Native Hawaiian or Other Pacific Islander (2076-8), or Unknown Race (2131-1).
4. **Ethnicity** encoded as Hispanic or Latino (2135-2), Not Hispanic or Latino (2186-5), or Unknown Ethnicity.
5. **Socioeconomic deprivation** encoded as the ICD-10 code for Problems related to housing and economic circumstances (Z59).
6. **Obesity** encoded as one dichotomous variable and one categorical variable: Overweight and obesity (E66) and body mass index (categorised into < 25 kg/m^2^, 25-30 kg/m^2^, ≥ 30 kg/m^2^ which are the WHO thresholds for not obese, pre-obese, and obesity).
7. **Hypertension** encoded as 2 dichotomous and 2 categorical variables: Hypertensive diseases (I10-I16), the now deprecated version that was used until 2018 Hypertension diseases (I10-I15), measurements of systolic blood pressure (categorised into < 140mmHg, 140-160mmHg, and ≥ 160mmHg), and diastolic blood pressure (categorised into < 90mmHg, 90-100mmHg, and ≥ 100mmHg). The blood pressure categories correspond to the absence of hypertension, stage 1 hypertension, and stage 2 (and over) hypertension as per the NICE guidelines.
8. **Diabetes** **mellitus** encoded as 2 dichotomous variables: Type 1 diabetes mellitus (E10) and Type 2 diabetes mellitus (E11).
9. **Chronic lower respiratory diseases** encoded by each sub-category of the corresponding ICD-10 group: Bronchitis, not specified as acute or chronic (J40), Simple and mucopurulent chronic bronchitis (J41), Unspecified chronic bronchitis (J42), Emphysema (J43), Other chronic obstructive pulmonary disease (J44), Asthma (J45), Bronchiectasis (J47).
10. **Nicotine dependence** encoded as the corresponding ICD-10 diagnosis (F17.2).
11. **Substance use disorders** encoded as the ICD-10 code for mental and behavioural disorders due to psychoactive substance use (F10-F19).
12. **Psychotic disorders** encoded as the ICD-10 code for schizophrenia, schizotypal, delusional, and other non-mood psychotic disorders (F20-F29).
13. **Mood disorders** encoded as the ICD-10 code for mood disorders (F30-F39).
14. **Anxiety disorders** encoded as the ICD-10 code for anxiety, dissociative, stress-related, somatoform and other nonpsychotic mental disorders (F40-F48).
15. **Heart diseases** encoded as 2 categorical variables: Ischaemic heart disease (I20-I25) and Other forms of heart disease (I30-I52).
16. **Chronic kidney disease** encoded as 2 dichotomous variables: Chronic kidney disease (N18) and Hypertensive chronic kidney disease (I12).
17. **Chronic liver disease** encoded as 8 categorical variables: Alcoholic liver disease (K70), Hepatic failure, not elsewhere classified (K72), Chronic hepatitis, not elsewhere classified (K73), Fibrosis and cirrhosis of liver (K74), Fatty (change of) liver, not elsewhere classified (K76.0), Chronic passive congestion of liver (K76.1), Portal hypertension (K76.6), Other specified diseases of liver (K76.8).
18. **Stroke** encoded as the dichotomous variable Cerebral infarction (I63) .
19. **Dementia** encoded as 6 dichotomous variables: Vascular dementia (F01), Dementia in other diseases classified elsewhere (F02), Unspecified dementia (F03), Alzheimer's disease (G30), Frontotemporal dementia (G31.0), and Dementia with Lewy bodies (G31.83).
20. **Cancer and haematological cancer in particular** encoded as 2 dichotomous variables: Neoplasms (C00-D49) and Malignant neoplasms of lymphoid, hematopoietic and related tissue (C81-C96).
21. **Organ transplant** encoded as 2 dichotomous variables: Renal Transplantation Procedures and Liver Transplantation Procedures.
22. **Rheumatoid arthritis** encoded as 2 dichotomous variables: Rheumatoid arthritis with rheumatoid factor (M05) and Other rheumatoid arthritis (M06).
23. **Lupus** encoded as a dichotomous variable corresponding ICD-10 code (M32).
24. **Psoriasis** encoded as a dichotomous variable corresponding ICD-10 code (L40).
25. **Disorders involving an immune mechanism** encoded as a dichotomous variable “Certain disorders involving the immune mechanism” (D80-D89).

Each individual code was considered a confounding factor in and of itself so that matching was achieved for each of them individually. For instance, matching was achieved for each subcategory (and not just for the whole category) of chronic lower respiratory diseases. For variables representing diagnoses and socioeconomic deprivation, an individual was considered positive if the diagnostic was recorded at least once in their health record before the index event. For categorical variables representing measurements (i.e. BMI and blood pressures), all available measurements for all individuals were used and propensity score matching sought to define cohorts with similar numbers of measurements falling into each category.

## D Definition of outcomes

*ICD-10 codes*

All outcomes were defined as an event recorded in the patient’s electronic health. Specifically, the following ICD-10 codes (with the ICD-10 labels in brackets) were used to define outcomes:

1. **Chest/Throat pain**: R07 (‘Pain in throat and chest’).
2. **Abnormal breathing**: R06 (‘Abnormalities of breathing’).
3. **Abdominal symptoms**: R10 (‘Abdominal and pelvic pain’), R19.4 (‘Change in bowel habit’), or R19.7 (‘Diarrhoea, unspecified’).
4. **Fatigue**: G93.3 (‘Postviral fatigue syndrome’) or R53 (‘Malaise and fatigue’).
5. **Anxiety/Depression**: F30-F39 (‘Mood disorders’) or F40-F48 (‘Anxiety, dissociative, stress-related, somatoform and other nonpsychotic mental disorders’). F30-39 also encompasses diagnoses other than depression (e.g. mania), but these comprise only a small fraction of cases and so we use Anxiety/Depression for convenience.
6. **Pain**: G89 (‘Pain not elsewhere specified’) or R52 (‘Pain, unspecified’).
7. **Headache**: R51 (‘Headache’), G43 (‘Migraine’), or G44 (‘Other headache syndrome’).
8. **Cognitive symptoms**: R40 (‘Somnolence, stupor and coma’), R41 (‘Other symptoms and signs involving cognitive functions and awareness’), R48 (‘Dyslexia and other symbolic dysfunction’), G93.40 (‘Encephalopathy, unspecified’), G31.84 (‘Mild cognitive impairment’), G30 (‘Alzheimer’s disease’), G31.0 (‘Frontotemporal dementia’), G31.83 (‘Dementia with Lewy bodies’), F01 (‘Vascular dementia’), F02 (‘Dementia in other disease classified elsewhere’), F03 (‘Unspecified dementia’), F05 (‘Delirium due to known physiological condition’), or F06.8 (‘Other specified mental disorders due to known physiological condition’). These terms were used to capture the range of diagnostic codes that patients presenting with ‘brain fog’ might receive.
9. **Myalgia**: M79.1 (‘Myalgia’) or M60 (‘Myositis’). Inclusion of the latter category was intended to capture those patients who received a specific diagnosis when presenting with myalgia.
10. **Atopic dermatitis (used as a negative control)**: L20 (‘Atopic dermatitis’)

*Comments on the use of EHR to characterize clinical features*

If one is interested in the number of people who suffer with a headache within the 6 months after a diagnosis of COVID-19, they face a practical question: What ought we to consider a headache worth counting? Counting all headaches (however mild and transient) might not be the quantity of interest. One objective definition which also captures the patient’s subjective experience would be to count “any headache that the patient sought medical attention for”. This approach to defining thresholds on the features has three advantages: (i) it can be captured in EHR data, (ii) it not only represents the burden for the patient but also the burden for healthcare systems, and (iii) it can be applied universally to different symptoms (otherwise, how are we to decide how severe a constipation ought to be to match the severity threshold that we have set on headaches?). EHR thus provide data on clinical features for which thresholds are defined based on the patient seeking medical attention.

## E Details on statistical analyses

*Separately assessing the incidence of features based on the time window during which they occurred*

If a feature was recorded for an individual in the 6 months after a COVID-19 diagnosis, then there are three possibilities:

- This feature in this individual was recorded in the first 3 months after a COVID-19 diagnosis but not in the next 3 months (we name the incidence of such features *I_1_*).
- This feature in this individual was recorded in the 3-6 months after a COVID-19 diagnosis but not in the first 3 months (*I_2_*).
- This feature in this individual was recorded both in the first 3 months and in the next 3 months after a COVID-19 diagnosis (*I_12_*).

We can estimate *I_1_*, *I_2_*, and *I_12_*, using the Kaplan-Meier estimators over the whole period and over the ‘long’ phase only. Let us denote by:

- *I(1,180)* the Kaplan-Meier estimated cumulative incidence of a long-COVID feature over the whole 180 days (i.e. the value at the end-timepoint of the Kaplan-Meier curves with the 1-180 days time window),
- *I(1,90)* the Kaplan-Meier estimated cumulative incidence of a long-COVID feature over the first 90 days (i.e. the value at the mid-timepoint of the Kaplan-Meier curves with the 1-180 days time window), and
- *I(90,180)* the Kaplan-Meier estimated cumulative incidence of a long-COVID feature over the 90-180 days (i.e. the value at the end-timepoint of the Kaplan-Meier curves with the 90-180 days time window).

We have the following relationships:

*I_2_ = I(0,180)- I(0,90)*,

*I_12_ = I(90,180)-I_2_*, and

*I_1_ = I(0,90)-I_12_*.

The first equation expresses that the number of patients who experienced the feature only in the ‘long’ phase are all those which experienced the feature at some point in the either the ‘earlier’ or ‘long’ phase occurred minus those who experienced the feature in the ‘earlier’ phase. The second equation expresses that those who experienced the feature in both the ‘earlier’ and ‘long phase’ equals all those who experienced the feature in the ‘long’ phase (i.e. both first and recurrent feature) minus those who only experienced the feature in the ‘long’ phase. The third equation expresses that the number of patients who experienced the feature in the ‘earlier’ phase only equals the total number of those who experienced it in the ‘earlier’ phase minus those who experienced it in both the ‘earlier’ and the ‘long’ phases. One can check that *I_1_+I_2_+I_12_* equals *I(0,180)*, as expected. The proportion *I_1_*, *I_2_*, and *I_12_* are represented in different shades in Figure 1 of the main manuscript.

*Implementation details of propensity score matching*

In propensity score matching, the propensity score was calculated using a logistic regression (implemented by the function LogisticRegression of the scikit-learn package in Python 3.7) including each of the covariates mentioned above. To eliminate the influence of ordering of records, the order of the records in the covariate matrix were randomised before matching.

*Testing proportional hazards*

The assumption that the hazards were proportional when accounting for the two phases was tested using the generalized Schoenfeld approach [11] implemented in the cox.zph function of the survival package (version 3.2.3) in R. If the proportional hazard assumption was found to be violated (i.e. statistical evidence from a score test indicating a non-zero slope in the scaled Schoenfeld residuals over time), then the time-varying HR was assessed using natural cubic splines (in log-time) to the log-cumulative hazard [12]. This was achieved using the generalized survival models of the rstpm2 package (version 1.5.1) in R [13]. As recommended by Royston and Parmar [12], splines with 1, 2, and 3 degrees of freedom were estimated for both the baseline log-cumulative hazard and its cohort dependency and the number of degrees of freedom leading to the lowest Akaike Information Criterion (AIC) was selected. This was achieved on a per-comparison basis so that more complex time dependency (i.e. higher number of degrees of freedom) could be selected for a specific comparison if there was enough evidence in the data to support such complexity.

*Network of clinical features*

The probability of clinical feature *A* occurring in a cohort between two time points (*t_1_* and *t_2_*) can be calculated as:

$$P_{A}\left( t_{1},t_{2} \right)=P_{A}\left( t_{2} \right)-P_{A}\left( t_{1} \right),$$

where $P_{A}\left( t \right)$ is the probability of having had the outcome between the beginning of the follow-up period and time *t* and is simply obtained from the Kaplan-Meier estimator. Similarly, the probability $P_{A\&B}\left( t_{1},t_{2} \right)$of co-occurrence of two clinical features *A* and *B*, can be calculated using the Kaplan-Meier estimator for the occurrence of pairs of features. From those estimates, we can calculate Dice’s coefficient [14] as follows:

$$D_{A,B}\left( t_{1},t_{2} \right)= \frac{P_{A\&B}(t_{1},t_{2})}{\frac{1}{2}\left( P_{A}\left( t_{1},t_{2} \right)+P_{B}\left( t_{1},t_{2} \right) \right)}$$

Dice’s coefficient can be calculated for each pair of clinical features and a network is thereby formed wherein clinical features are nodes, and the connection between them is weighted by the corresponding Dice coefficient. We calculate the degree of each clinical feature in the network as the sum of its Dice’s coefficients with the other 8 clinical features, and the average degree as the mean of all 9 degrees.

Dice’s coefficient (and hence the degree of each node and average degree of the network) can be calculated for any time window (spanning from t_1_ to t_2_). In the primary analysis, we set *t_1_* to 0 and *t_2_* to 180 days to assess the network of clinical features over the whole follow-up period. We then calculate Dice’s coefficients and average degree using a rolling window of 14 days (i.e. from 1 to 15 days, 2 to 16 days, etc.) to assess the evolution of Dice’s coefficients over time. The change in average degree over time is assessed using a linear regression in which the average degree (one observation per time window) is the dependent variable and time is the independent variable (using the *lm* function in R). This results in a coefficient representing the change in average degree per unit time. The 95% confidence interval and p-value for the null hypothesis that this coefficient equals zero was calculated using non-parametric bootstrap as outlined below.

*Non-parametric bootstrap*

The confidence intervals for the value of the Kaplan-Meier curve at each time point, for the hazard ratios, for the average degree of the networks, and for the change in time of the average degree, were estimated from 1000 bootstrap replicates using a first-order normal approximation, and were corrected for bootstrapping sampling bias [15]. The p-value for the hazard ratio was calculated by identifying the widest confidence interval which excluded 1. Bootstrapping was implemented in R using the *boot* package.

*Permutation test*

The null hypothesis that the mean degree of the network of clinical features was equal between cohorts was tested using permutation test. The patients’ ID were randomly permuted 1000 times across both cohorts. For each permutation, the network of clinical features were calculated for each group thereby generated, and the difference (in absolute value) between their mean degree was computed. This effectively generates a null distribution of the difference in mean degree from which a p-value can be calculated.

## F Details on secondary analyses

To assess whether differences in demographics and differences in severity of COVID-19 are associated with differences in occurrence and co-occurrence of long-COVID features, 8 additional cohort studies were performed, each comparing two subgroups of patients diagnosed with COVID-19 as follows:

Female vs. Male

Non-white (defined as all those with a race recorded as Black or African American, Asian, American Indian or Alaska Native, Native Hawaiian or Other Pacific Islander) or White (defined as all those with a race recorded as White)

Patients with age 45 and over vs. age 10-44 (to assess broad differences among the younger and older patients in the cohort)

Patients with age 65 and over vs. age 45-64 (to further assess specific differences among older adults)

Patients with age 22-44 vs. age 10-21 (to further assess specific differences among adolescents and young adults)

Patients requiring vs. not requiring hospitalisation. Hospitalisation was defined as any hospital visit within a time window from 4 days before their COVID-19 diagnosis (taken to be the time it might take between clinical presentation and confirmation) to 2 weeks afterwards.

Patients requiring vs. not requiring intensive treatment unit (ITU) care (within a time window from 4 days before to 2 weeks after the date of their COVID-19 diagnosis)

Patients with vs. without leukocytosis. Leukocytosis was defined as any white cell count (WCC) over 11,000 per microliter recorded between 4 days before and 2 weeks after the diagnosis of COVID-19. Patients *without* leukocytosis were defined as all those who had at least one WCC recorded during the same time window but for whom WCC results were all below 11,000 per microliter. These definitions mean that all patients in the two cohorts had a least one WCC measurement during this time period, suggesting that their COVID-19 illness was considered severe enough to warrant a blood test.

Each of these additional cohort studies were conducted in the exact same way as the primary analysis except that the characteristic of interest was obviously not included as part of the covariates for matching (e.g. we did not match for sex when comparing females and males).

# References

1. Taquet M, Luciano S, Geddes JR, Harrison PJ. Bidirectional associations between COVID-19 and psychiatric disorder: retrospective cohort studies of 62 354 COVID-19 cases in the USA. Lancet Psychiatry. 2021;8: 130–140.

2. Taquet M, Geddes JR, Husain M, Luciano S, Harrison PJ. 6-month neurological and psychiatric outcomes in 236 379 survivors of COVID-19: a retrospective cohort study using electronic health records. Lancet Psychiatry. 2021;8: 416–427.

3. Casey JA, Schwartz BS, Stewart WF, Adler NE. Using Electronic Health Records for Population Health Research: A Review of Methods and Applications. Annu Rev Public Health. 2016;37: 61–81.

4. Cowie MR, Blomster JI, Curtis LH, Duclaux S, Ford I, Fritz F, et al. Electronic health records to facilitate clinical research. Clin Res Cardiol. 2017;106: 1–9.

5. Jetley G, Zhang H. Electronic health records in IS research: Quality issues, essential thresholds and remedial actions. Decis Support Syst. 2019;126: 113137.

6. de Lusignan S, Dorward J, Correa A, Jones N, Akinyemi O, Amirthalingam G, et al. Risk factors for SARS-CoV-2 among patients in the Oxford Royal College of General Practitioners Research and Surveillance Centre primary care network: a cross-sectional study. Lancet Infect Dis. 2020. doi:10.1016/S1473-3099(20)30371-6

7. Zhang J-J, Dong X, Cao Y-Y, Yuan Y-D, Yang Y-B, Yan Y-Q, et al. Clinical characteristics of 140 patients infected with SARS-CoV-2 in Wuhan, China. Allergy. 2020. Available: https://onlinelibrary.wiley.com/doi/abs/10.1111/all.14238

8. Chen N, Zhou M, Dong X, Qu J, Gong F, Han Y, et al. Epidemiological and clinical characteristics of 99 cases of 2019 novel coronavirus pneumonia in Wuhan, China: a descriptive study. Lancet. 2020;395: 507–513.

9. Wang QQ, Kaelber DC, Xu R, Volkow ND. COVID-19 risk and outcomes in patients with substance use disorders: analyses from electronic health records in the United States. Mol Psychiatry. 2021;26: 30–39.

10. Williamson EJ, Walker AJ, Bhaskaran K, Bacon S, Bates C, Morton CE, et al. Factors associated with COVID-19-related death using OpenSAFELY. Nature; 584: 430–436.

11. Grambsch PM, Therneau TM. Proportional hazards tests and diagnostics based on weighted residuals. Biometrika. 1994;81: 515.

12. Royston P, Parmar MKB. Flexible parametric proportional-hazards and proportional-odds models for censored survival data, with application to prognostic modelling and estimation of treatment effects. Statistics in Medicine. 2002. pp. 2175–2197. doi:10.1002/sim.1203

13. Liu X-R, Pawitan Y, Clements M. Parametric and penalized generalized survival models. Stat Methods Med Res. 2018;27: 1531–1546.

14. Dice LR. Measures of the amount of ecologic association between species. Ecology. 1945;26: 297–302.

15. Efron B, Gong G. A leisurely look at the bootstrap, the jackknife, and cross-validation. Am Stat. 1983;37: 36–48.
